# Evolutionary history of the *iroquois/Irx *genes in metazoans

**DOI:** 10.1186/1471-2148-9-74

**Published:** 2009-04-15

**Authors:** Pierre Kerner, Aissam Ikmi, Dario Coen, Michel Vervoort

**Affiliations:** 1 Evolution et Développement des métazoaires, Centre de Génétique Moléculaire – FRE 3144 CNRS, 1, av. de la terrasse, 91198 Gif-sur-Yvette, France; 2 UFR Sciences du Vivant, Université Paris Diderot – Paris 7, 5 rue Marie-Andrée Lagroua Weill-Hallé, 75205 Paris Cedex 13, France; 3 Institut Jacques Monod, UMR 7592 CNRS/Université Paris Diderot – Paris 7, 15, rue Hélène Brion, 75205 Paris Cedex 13, France; 4 Développement, Morphogenèse et Évolution, CNRS UMR 8080, Université Paris-Sud, 91405 Orsay, France; 5 Present address: Stowers Institute for Medical Research, 1000 E 50th St Kansas City, Missouri 64110 USA

## Abstract

**Background:**

The *iroquois *(*iro/Irx*) genes encode transcriptional regulators that belong to the TALE superclass of homeodomain proteins and have key functions during development in both vertebrates and insects. The *Irx *genes occur in one or two genomic clusters containing three genes each within the *Drosophila *and several vertebrate genomes, respectively. The similar genomic organization in *Drosophila *and vertebrates is widely considered as a result of convergent evolution, due to independent tandem gene duplications. In this study, we investigate the evolutionary history of the *Irx *genes at the scale of the whole metazoan kingdom.

**Results:**

We identified *in silico *the putative full complement of *Irx *genes in the sequenced genomes of 36 different species representative of the main metazoan lineages, including non bilaterian species, several arthropods, non vertebrate chordates, and a basal vertebrate, the sea lamprey. We performed extensive phylogenetic analyses of the identified *Irx *genes and defined their genomic organizations. We found that, in most species, there are several *Irx *genes, these genes form two to four gene clusters, and the *Irx *genes are physically linked to a structurally and functionally unrelated gene known as *CG10632 *in *Drosophila*.

**Conclusion:**

Three main conclusions can be drawn from our study. First, an *Irx *cluster composed of two genes, *araucan/caupolican *and *mirror*, is ancestral to the crustaceans+insects clade and has been strongly conserved in this clade. Second, three *Irx *genes were probably present in the last common ancestor of vertebrates and the duplication that has given rise to the six genes organized into two clusters found in most vertebrates, likely occurred in the gnathostome lineage after its separation from sea lampreys. Third, the clustered organization of the *Irx *genes in various evolutionary lineages may represent an exceptional case of convergent evolution or may point to the existence of an *Irx *gene cluster ancestral to bilaterians.

## Background

Gene duplication clearly plays an important role in generating molecular diversity. In some cases, these duplications arise through the duplication of entire chromosomes or large chromosomal regions. In other cases, duplications appear as tandem copies of genes, which form clusters of evolutionarily related genes. Two evolutionary questions are raised by the latter situation. When did these clusters form and what are the evolutionary forces that act to maintain these genes clustered? One of the most intensively studied cases of clustered genes is that of the *Hox *genes [1]. As complete genome sequences become available, we can begin to track the evolutionary history of many other clusters of evolutionarily related genes. One gene family which displays genomic linkage of unknown functionality and origin is the *iroquois *(*iro/Irx*) family.

The *Irx *genes encode transcription factors that are involved in many developmental processes in metazoans [2,3]. The Irx proteins belong to the TALE (three aminoacid loop extension) superclass of homeodomain proteins and are characterized by the presence, in addition to the homeodomain, of two conserved specific domains of unknown functions, named "IRO A" and "IRO box" [3-5]. Within the TALE superclass of homeobox genes, the *Irx *genes appear closely related to the *mohawk *(*Mkx*, also known as *iroquois-like*, *Irxl*) genes which encode proteins with a homeodomain similar to that of the Irx proteins, but lack the Irx-specific domains and harbour other conserved domains (the "MKX A", "MKX B" and "MKX C" domains) [5-7].

Three *Irx *genes – *araucan*, *caupolican*, and *mirror *– have been identified in *Drosophila*, and they form a gene complex (the so-called *iroquois *complex, Iro-C) that is involved, during larval development and metamorphosis, in the formation of sense organs (including the eyes), in the specification of the dorsal part of the adult thorax and in the patterning of the wing veins, as well as in the segmentation of the body during embryonic development [2,3]. Six *Irx *genes, organized into three-gene complexes (IrxA which contains the *Irx1*, *Irx2 *and *Irx4 *genes and IrxB which contains the *Irx3*, *Irx5 *and *Irx6 *genes) have been isolated in mammals and have been shown to have key roles during development, e.g. in neurogenesis and in the patterning of the heart [2,3]. Orthologs of these genes have been found in other vertebrates, such as the zebrafish in which 11 *Irx *genes are organized into four clusters [8,9]. A single *Irx *gene has been identified in the nematode *Caenorhabditis elegans *[4], but its function has not been characterized. *Irx *genes have also been identified in a few other species, such as sponges, but they have not been studied at the functional level [e.g. [5,10-12]].

The presence of several *Irx *genes and their organization into three-gene complexes in both vertebrates and *Drosophila *have raised several questions about the evolutionary history of these genes. First, are the *Drosophila *and vertebrates clusters homolog, already present in the last common ancestor of these species (this ancestor is known as *Urbilateria *as it is the ancestor of all bilaterians, the animals displaying a bilateral symmetry)? Comparisons of the vertebrates and *Drosophila Irx *genes suggest that the vertebrate genes are more similar to one another than to their *Drosophila *counterpart, suggesting that the gene duplications that have given rise to the *Drosophila *and vertebrate complexes occurred independently and therefore that these complexes do not derive from an ancestral cluster present in *Urbilateria *[e.g. [3,13,14]]. However, this conclusion is based on a very small sampling of species and establishing a firmly-based scenario for *Irx *genes evolution in bilaterians would require a broader sampling of the species in which these genes are characterized. Such a study has been recently published and the occurrence of independent duplications has been advocated by the authors of this study [12]. Second, what is the origin of the 2 or 4 complexes observed in vertebrates? Several lines of evidence indicate that the two clusters found in mammals (and other vertebrates such as birds) derive from an ancestral *Irx *cluster as a consequence of a chromosomal duplication event [3,13,14]. Indeed, *Irx1*, *Irx2 *and *Irx4 *are most similar to *Irx3*, *Irx5 *and *Irx6*, respectively, suggesting that *Irx1/Irx3*, *Irx2/Irx5 *and *Irx4/Irx6 *are pairs of paralogs. Furthermore, the organization of the clusters, including the orientation of the transcription of the genes, is very similar and paralogous genes flank the clusters in both mouse and zebrafish. In this latter (and in other teleosts), the two additional clusters that are found, would have been generated by a teleosts-specific genome duplication (whose occurrence is now well documented; e.g. [8,9,15]). It is, however, not clear when the ancestral vertebrate cluster was established and when the suggested chromosomal duplication occurred. Answering these questions requires data from more basal vertebrates and non-vertebrate chordates and non-chordate deuterostomes.

In a previously published study, Irimia et al. [12] reported the existence of more than one Irx gene in several metazoan genomes and these genes were in most cases clustered. A phylogenetic analysis of these genes suggests that these genes were produced by several independent duplications. In this article, we significantly extend this analysis by retrieving and analyzing, at the phylogenetic and genomic levels, the *Irx *genes encoded by several newly-sequenced metazoan genomes. We confirmed that in most species there are several *Irx *genes and that these genes are clustered. We performed multiple phylogenetic analyses of these sequences with up-to-date phylogenetic methods. Taken together, our data suggest two possible alternative evolutionary scenarios for the evolution of *Irx *genes in animals: either several independent tandem duplications events have occurred in the different bilaterian lineages and selective pressures independently acted in each of these lineages to maintain the genes clustered (in agreement with [12]), or a complex of *Irx *genes is ancestral to bilaterians and has been conserved in most species, but differential evolutionary rates have obscured the orthology relationships between genes from the different bilaterian lineages. Both hypotheses are supported by parts of the data and we do not think we can favor one of the scenarios over the other one.

## Results and discussion

### Identification of the Irx and Mkx genes from the fully-sequenced genomes of 32 metazoan species

**Figure 1:**
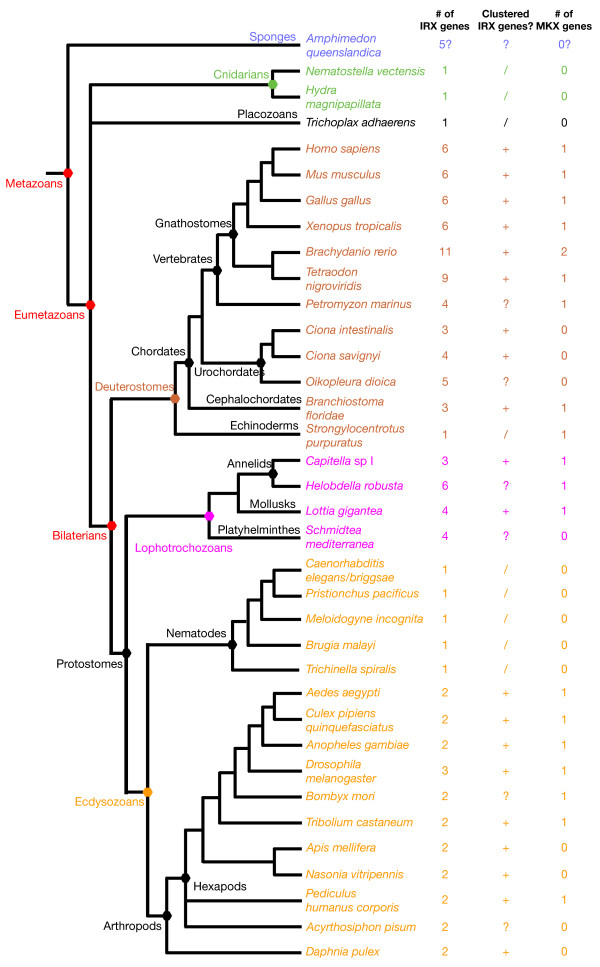
**Phylogenetic relationships between the species used in this study**. The number of *Irx and Mkx *genes in each genome and the potential clustering of the *Irx *genes are indicated. '+' indicates that the *Irx *genes form one or more clusters, '-' indicates that there is no clustering, '?' indicates that the current state of the assembly of the genome of the corresponding species does not allow determination of the existence of clusters, and '/' indicates cases where there is only a single *Irx *gene. The names of representative phylogenetic groups are indicated on the left of the nodes that define these different groups and along some of the terminal branches. The identification of *Irx *genes in some of the indicated species were already reported in other studies and our data are in full agreement with the previously published ones [4,5,9,11-13,37]. The color code for some of the metazoan evolutionary lineages (sponges, cnidarians, placozoans, trochozoans, deuterostomes, and ecdysozoans) will also be used in the next figures.

We used the sequences of *Irx *and *Mkx *genes from *Homo sapiens *and *Drosophila melanogaster *to identify, through similarity searches using BLAST algorithm, the *Irx *and *Mkx *genes encoded in the genomes of several species which provide significant coverage of the main metazoan evolutionary lineages (Figure 1). In most cases, we were able to retrieve the complete homeodomain as well as the additional conserved regions of the Irx and Mkx proteins [5]. Given our extensive searches, we think that the retrieved *Irx *and *Mkx *genes represent the full gene complement for these two families within each analyzed genome. All the identified sequences can be found in Additional file 1. A multiple sequence alignment of the conserved domains of the Irx and Mkx proteins can be found in Additional file 2.

**Figure 2:**
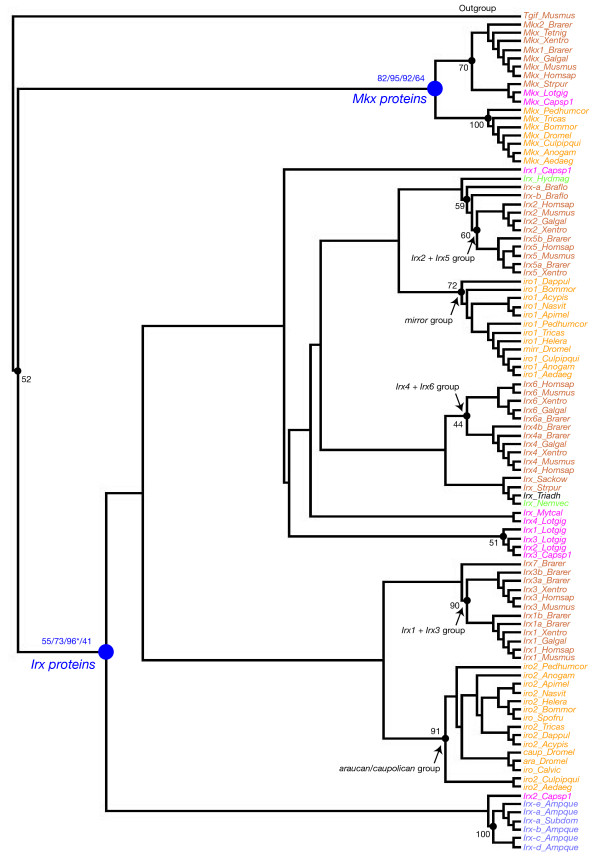
**Phylogenetic analysis of *Irx *and *Mkx *genes**. The represented tree is a maximum-likelihood tree, which has been rooted using the mouse TALE homeodomain TGIF as an outgroup. This tree is based on a multiple alignment that includes the homeodomain plus a few flanking aminoacids of most of the retrieved Irx and Mkx. Similar relationships (with similar statistical supports) were found when we used the entire set of Mkx and Irx sequences. The Irx and Mkx groups are indicated in blue and the associated numbers are their statistical support values obtained with different methods of phylogenetic reconstruction: first number is the bootstrap support in maximum-likelihood analysis (500 bootstrap replicates); second number is the posterior probabilities in Bayesian inference-based analysis; third number is the bootstrap support in neighbour-joining analysis (10,000 bootstrap replicates); fourth number is the bootstrap support in maximum-parsimony analysis (1,000 bootstrap replicates). The asterisk associated with the support in the neighbour-joining analysis indicates that in the neighbour-joining tree, the strongly supported Irx group does not include the sponge genes which are found as outgroup to both Irx and Mkx proteins. Statistical support in the maximum-likelihood analysis for some other internal branches is indicated in black. Only statistical support values >50% are shown except for a few cases.

We used phylogenetic analyses and the presence of the Irx and Mkx specific domains to define the respective complement of *Irx *and *Mkx *genes. A representative phylogenetic tree is shown in Figure 2. This tree is based on a multiple sequence alignment of the region that is conserved between Mkx and Irx proteins, i.e. the homeodomain plus a few flanking amino acids (see Additional file 2). Given the high number of sequences, we excluded from this alignment some sequences to simplify the tree, in particular the more divergent ones, such as those from the leech *Helobdella*. A similar tree topology was obtained using an alignment including all sequences (not shown). We also included the putative *Irx *gene that has been cloned from the sponge *Suberites domuncula *[10] as its affiliation to either the *Irx *or the *Mkx *families was not clear [5]. We found a well-supported monophyletic group that includes the known Mkx proteins together with several newly-identified putative Mkx proteins (Figure 2). This phylogenetic analysis and the presence of Mkx-specific conserved domains in these proteins (Additional file 2) allow their clear-cut identification as Mkx proteins. Another monophyletic group includes the known Irx proteins and a large number of other newly-identified putative Irx proteins (Figure 2). Although this group has poor statistical support, the presence of the Irx-specific conserved domains in most of the newly-identified proteins within this monophyletic group, allowed their safe identification as Irx proteins (Additional files 1 and 2). The proteins from the sponge *Suberites *and *Amphimedon *cluster with the Irx proteins in our phylogenetic analyses supporting the hypothesis that the corresponding sponge genes are *bona fide Irx *genes, as previously suggested by Larroux et al. [11]. However, we have to notice that these proteins lack the Irx-specific domains and are much divergent with respect to bilaterian Irx proteins (as well as to Mkx proteins; Additional file 2).

When reported on the species phylogeny, the numbers of identified *Irx *and *Mkx *genes indicate contrasting trends in the evolution of these two families (Figure 1). First, *bona fide Mkx *genes cannot be found in non-bilaterian species in contrast to *Irx *genes which are found, at least in cnidarians and the placozoan *Trichoplax*, as well as probably in sponges. We are therefore faced with two alternative hypotheses: either the *Mkx *genes are ancestral to metazoans and have been lost in the analysed non-bilaterian species, or the *Mkx *genes may represent an innovation of bilaterians and could be considered as bilaterian-specific divergent *Irx *genes. Second, while *Mkx *genes are found in both protostomes and deuterostomes (and are therefore likely to be already present in *Urbilateria*), several unrelated species (13 out of 36) lack the *Mkx *gene, indicating several independent events of gene loss. In contrast, *Irx *genes are found in all studied species indicating strong evolutionary pressures to conserve these genes. Third, while *Mkx *genes are usually found as a single gene in each species, we found several *Irx *genes in most cases (27 out of 36 metazoan species, 26 out of 32 if we only consider bilaterians). This indicates that the *Irx *(but not the *Mkx*) gene family evolution has been shaped by gene duplications. We further studied these gene duplications by phylogenetic analyses and characterizing the genomic organization of the *Irx *genes.

### Phylogenetic analyses of the Irx genes suggest the occurrence of many independent duplication events in protostomes and deuterostomes

**Figure 3:**
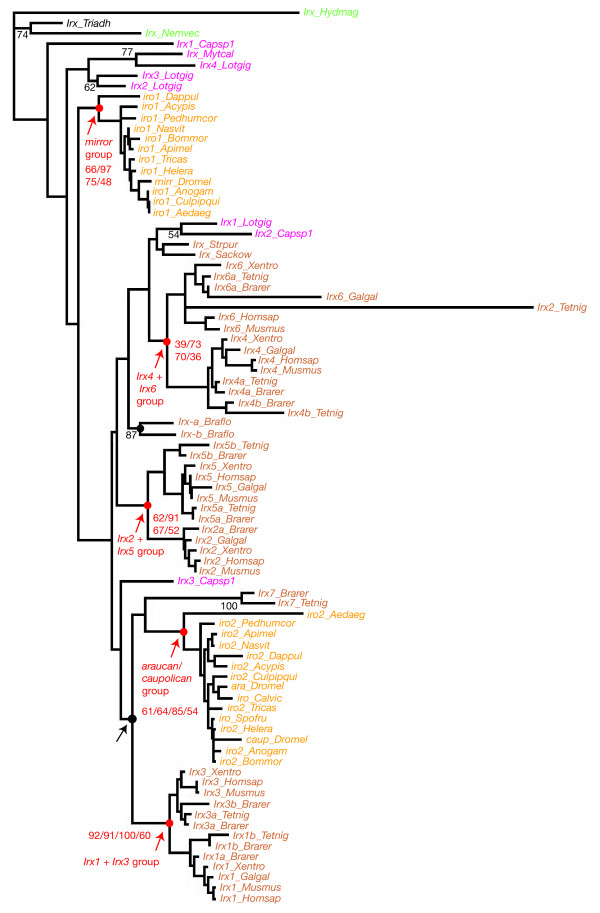
**Phylogenetic analysis of *Irx *genes in metazoans**. The represented tree is a maximum-likelihood tree, which has been rooted using the *Irx *gene from *Hydra *as outgroup (this should be considered as arbitrary rooting). This tree is based on a multiple alignment that includes the Irx conserved domains. The most important monophyletic groups are indicated in red and the associated numbers are their statistical support values obtained with different methods of phylogenetic reconstruction, as described in Figure 2. Statistical support in the maximum-likelihood analysis for some other internal branches is indicated in black. Only statistical support values >50% are shown except for a few cases. Other internal branches (with statistical support <50%) should be considered unreliable. The black arrow points to the group which is discussed in the main text.

We first analysed a large sampling of *Irx *genes representative of the main metazoan lineages (Figure 3). We used a multiple sequence alignment that includes both the homeodomain and the additional conserved domains (Additional file 2). We excluded the most divergent sequences from this alignment, including those from sponges that lack the Irx specific domains. We found several monophyletic groups, most of which were already observed in the phylogenetic tree that includes the *Mkx *genes (Figure 2). These groups (which will be detailed below) include either arthropods ("*mirror*" and "*araucan/caupolican*" groups), or vertebrates ("*Irx4/Irx6*", "*Irx2/Irx5*", and "*Irx1/Irx3*" groups), or lophotrochozoans (one group with an annelid and a mollusc gene). However, we did not find any statistically significant groups that include arthropods and other protostomes, nor protostomes and deuterostomes sequences. For example, while the "*araucan/caupolican*" (arthropod sequences) clusters with the "*Irx1/Irx3*" group (vertebrates sequences) in the ML tree (black arrow in Figure 3), this group has a very week statistical support (10%) and is not identified with the other phylogenetic reconstruction methods (not shown). A similar situation is observed for most of the other groups observed in the tree in Figure 3. This phylogenetic analysis therefore suggests that several independent duplications have occurred in protostomes and deuterostomes and that the presence of several *Irx *genes in many animals mainly represent evolutionary convergences. This result is in concordance with the conclusions of a previously published study based on a more limited set of *Irx *genes and less detailed phylogenetic analyses [12].

We next separately analyzed protostome and deuterostome *Irx *genes, as it allowed us to construct phylogenetic trees based on alignments which include many more aminoacid residues than when all metazoan sequences are considered. We excluded from our analyses the most divergent *Irx *sequences (those from *Helobdella*, *Ciona*, *Oikopleura*, and the different nematode species) in order to maximize the size of the unambiguously aligned portion of the proteins.

### Phylogenetic analyses of the protostome Irx genes suggest the presence of two ancestral Irx genes in arthropods and lophotrochozoans

**Figure 4:**
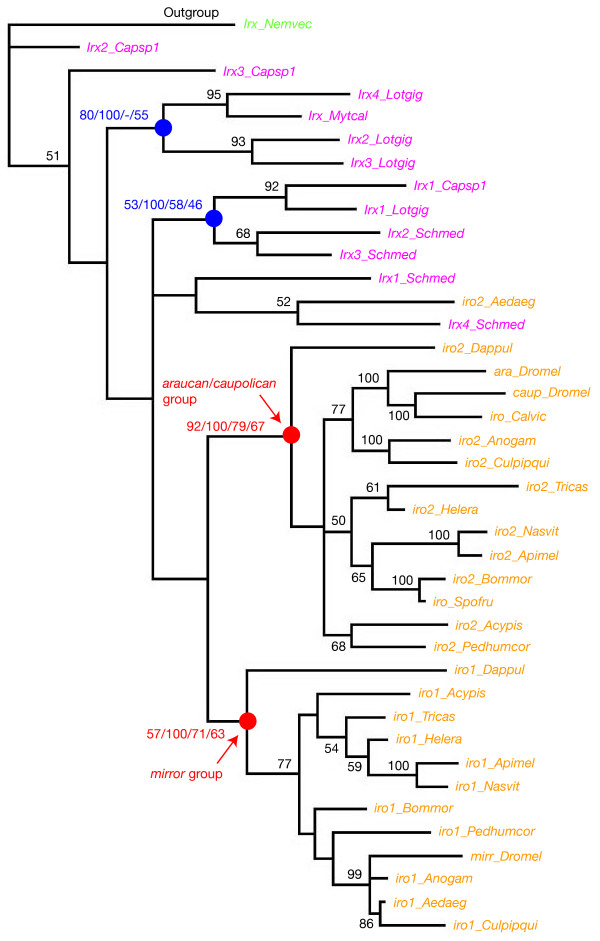
**Phylogenetic analysis of *Irx *genes in protostomes**. The represented tree is a maximum-likelihood tree, which has been rooted using the *Irx *gene from *Nematostella *as outgroup (this should be considered as arbitrary rooting). The most important monophyletic groups are indicated in red and the associated numbers are their statistical support values obtained with different methods of phylogenetic reconstruction, as described in Figure 2. Statistical support in the maximum-likelihood analysis for some other internal branches is indicated in black.

In protostomes, we found four monophyletic groups in the trees constructed by the different phylogenetic methods (Figure 4). Two of these groups only include arthropod sequences: one group includes the *Drosophila mirror *gene and one of the *Irx *gene found in the other analyzed arthropod species; the other includes the *Drosophila araucan *and *caupolican *genes and the other *Irx *gene(s) from the various arthropod species analyzed. To confirm the validity of these two monophyletic groups, we analyzed the arthropod sequences alone (this allowed to construct phylogenetic trees based on an alignment of the full-length proteins) and found strong support for the existence of monophyletic "*mirror*" and "*araucan/caupolican*" groups (Additional file 3). Since each of these two groups includes one gene from *Daphnia pulex *(a crustacean) and one or two genes from every studied insect, the presence of two *Irx *genes represents the ancestral situation for the crustaceans+insects ('pancrustacea') clade. These data therefore show the occurrence of an ancient duplication event in the arthropod lineage. A second duplication happened much more recently in some dipterans, comprised in the brachycera lineage ("flies") that gave rise to the *araucan *and *caupolican *genes found in *Drosophila*. In the other dipteran lineage, the nematocera ("mosquitoes"), a single "*araucan/caupolican*" gene has been retained as seen in the three studied nematocera species, *Aedes*, *Culex*, and *Anopheles *(Figures 1 and 4). Our data therefore confirm the occurrence of two duplication events in the *Irx *gene family in arthropods, as previously suggested [5].

The two other protostome monophyletic groups concern lophotrochozoan sequences (Figure 4): one group includes three *Irx *genes from the limpet *Lottia *and one gene identified in an EST collection of the mussel *Mytilus *and therefore indicates the occurrence of duplications specific to molluscs. The other group includes *Irx *genes from three distantly-related species, the annelid *Capitella *(1 gene), the mollusc *Lottia *(1 gene), and the flatworm *Schmidtea *(2 genes). The other *Irx *genes from *Capitella *(2 genes), *Lottia *(3), and *Schmidtea *(2) do not cluster together (Figure 4). Our interpretation of this phylogenetic tree is that there were two *Irx *genes in the last common ancestor of the three aforementioned lophotrochozoan species and that one of the paralogs in each evolutionary lineage underwent highly divergent evolution (in such a way that these paralogs do not cluster in the phylogenetic trees).

Since an ancestral two gene situation is found for both arthropods and lophotrochozoans, it is therefore conceivable that the presence of two *Irx *genes may be ancestral to protostomes, but that differential evolutionary rates have obscured the orthology relationships between genes from the arthropod and lophotrochozoan lineages. We however have to note that a single *Irx *gene is found in several different nematode species (Figure 1) which belong, together with arthropods, to the ecdysozoans, one of the two main protostome branches. If our hypothesis of an ancestral two gene situation in protostomes is true, we therefore have to consider that one or several *Irx *gene losses have occurred in the nematode lineage. This is not unconceivable as it is known that strongly-conserved genes in bilatarians have been lost in nematodes, for example several *Hox *genes [16], and our study points to the loss of the *Mkx *genes in all the studied nematode species. We can however clearly not exclude that the presence of a single *Irx *gene in nematodes may represent the ancestral state in protostomes and that independent gene duplications occurred in arthropods and lophotrochozoans.

### Phylogenetic analyses of the deuterostome Irx genes indicate the presence of a single Irx cluster of at least 2 genes in the last common ancestor of present-day vertebrates and suggest gene losses in non vertebrate deuterostomes

**Figure 5:**
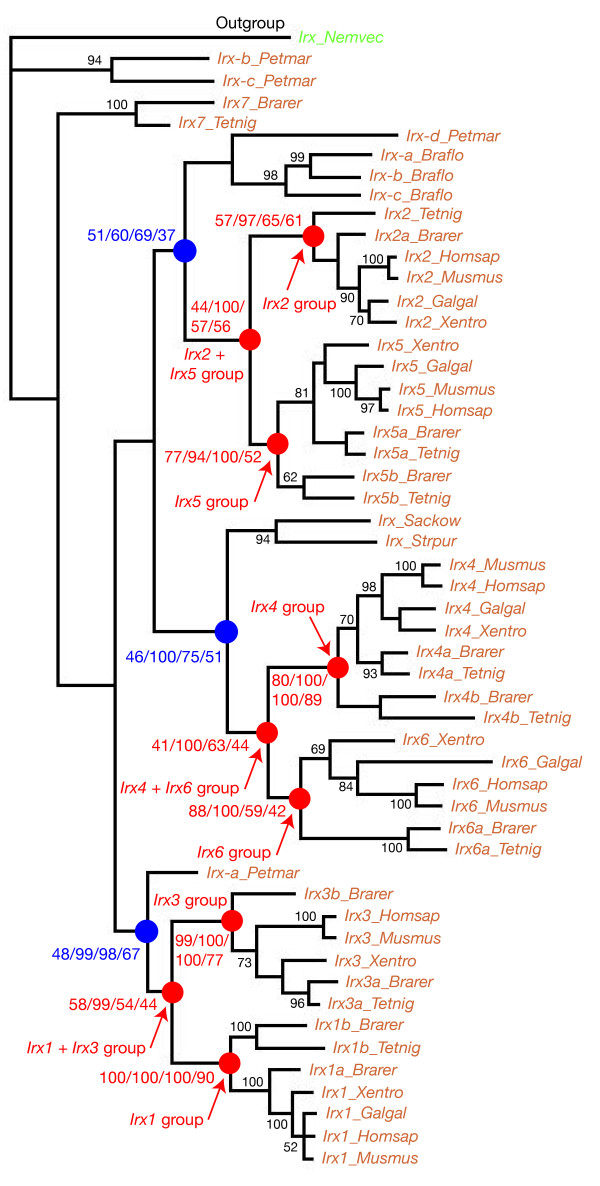
**Phylogenetic analysis of *Irx *genes in deuterostomes**. The represented tree is a maximum-likelihood tree, which has been rooted using the *Irx *gene from *Nematostella *as outgroup (this should be considered as arbitrary rooting). The most important monophyletic groups are indicated in red and blue. The associated numbers are their statistical support values obtained with different methods of phylogenetic reconstruction, as described in Figure 2. Statistical support in the maximum-likelihood analysis for some other internal branches is indicated in black.

We next focused on deuterostome sequences (Figure 5). We found in our phylogenetic trees the 6 previously described groups of *Irx *genes (*Irx1 *to *Irx6*) from mouse, human, *Xenopus*, zebrafish and pufferfish, as well as their association into three pairs of paralogs, *Irx1/Irx3*, *Irx2/Irx5 *and *Irx4/Irx6*. This confirms that the last common ancestor of the aforementioned vertebrate species (they all belong to the osteichthyan lineage of gnathostomes) already owned 6 *Irx *genes which have been produced by the duplication, earlier in vertebrate evolution, of 3 ancestral genes. The inclusion in our analysis of the *Irx *genes from a non gnathostome species, the sea lamprey *Petromyzon*, allowed us to further study the early evolution of *Irx *genes in vertebrates. We found two of the four *Petromyzon *genes to cluster with gnathostome groups, one as outgroup to the *Irx2*/*Irx5 *group and the other as outgroup to the *Irx1*/*Irx3 *group (Figure 5). This indicates that the last common ancestor of the sea lamprey and gnathostomes has one *Irx2*/*Irx5 *and one *Irx1*/*Irx3 *gene. No *Petromyzon *gene clusters with the gnathostome *Irx4/Irx6 *group and the two other *Petromyzon *genes (*Irx-b *and *Irx-c*) strongly cluster together but branch off from the gnathostome genes. We have to mention that for these genes we only retrieved a small part of their coding sequence despite extensive efforts (Additional files 1 and 2) and therefore incomplete sequences were used for the phylogenetic analyses. To our opinion, the most likely interpretation is that *Irx-b *and *Irx-c *derive from an ancestral *Irx4/Irx6 *gene that was independently duplicated in the evolutionary lineage leading to the sea lamprey and in gnathostomes. We think that the position of the *Irx-b *and *Irx-c *sequences at the root of the deuterostome *Irx *tree is due, at least in part, to the fact that partial sequences are used. Taken together, our data therefore suggest that there were three *Irx *genes in the last common ancestor of lampreys and gnathostomes, and that the chromosomal duplication that gave rise to the 6 aforementioned *Irx *groups occurred in the gnathostome lineage, after the split with non gnathostomes, such as sea lampreys (Figure 5). The identication of the full set of *Irx *genes in chondrychthyans would allow further definition of the timing of the duplication event.

We also studied *Irx *genes from urochordates and cephalochordates. Unfortunately, the *Irx *genes from the urochordates (the two *Ciona *species and *Oikopleura*) are very divergent and when included in the phylogenetic analyses, they perturb the overall topology of the trees and do not cluster with vertebrate sequences (not shown). The phylogenetic tree shown in Figure 5 therefore contains only the *Irx *genes from the cephalochordate *Branchiostoma *(amphioxus), as well as the only two *Irx *genes known from non-chordate deuterostomes, the single *Irx *gene encoded by the genome of the echinoderm *Strongylocentrotus *and the single *Irx *gene cloned (other *Irx *genes may exist) in the hemichordate *Saccoglossus *(hemichordates and echinoderms form a monophyletic group – the Ambulacria – within deuterostomes). The 3 *Branchiostoma Irx *genes strongly cluster together (and therefore derive from *Branchiostoma*-specific duplications) and with the *Irx2/Irx5 *group (statistical supports are not strong, but this clustering is found with all methods). Similarly, the Ambulacria *Irx *genes strongly cluster together and with the *Irx4/Irx6 *group. The fact that the *Branchiostoma Irx *genes, on one hand, and the Ambulacria *Irx *genes, on the other hand, cluster with different vertebrate *Irx *gene (*Irx2/Irx5 *and *Irx4/Irx6 *groups, respectively) suggest that there were at least two *Irx *genes in the last common ancestor of the deuterostomes, like in prostostomes. The fact that a third independent group (*Irx1/Irx3*) exists in vertebrates may even suggest an ancestral situation where three *Irx *genes form a cluster in deuterostomes (*Irx1/Irx3*, *Irx2/Irx5 *and *Irx4/Irx6*). In these views, we have to consider that the two or three ancestral genes would have been conserved in vertebrates (and subsequently duplicated), but one or two of them were independently lost in Ambulacria (*Irx4/Irx6 *remained) and cephalochordates (*Irx2/Irx5 *remained and was subsequently duplicated).

### Organization of the Irx genes in clusters is a general rule in bilaterians

**Figure 6:**
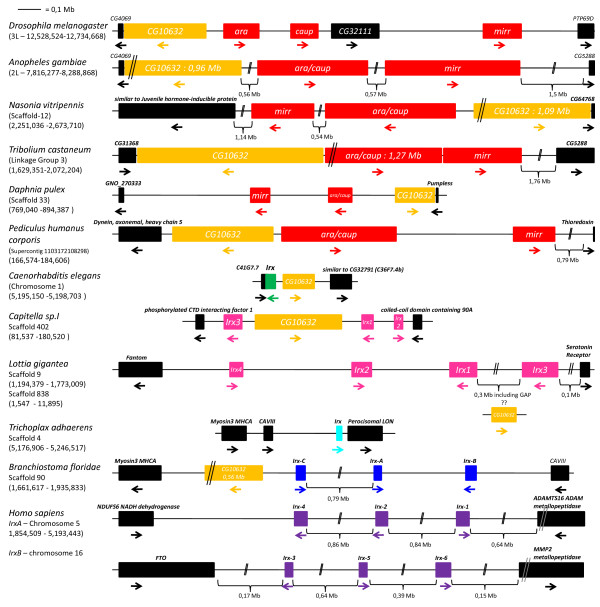
**Genomic organization of selected *Irx *genes**. The portion of the genome that includes the *Irx *gene(s) in several species is schematically depicted. The name of the genes that flank the *Irx *genes are those indicated in the genome browsers of the different species. In the case of *Lottia*, there is a gap in the genomic scaffold between *Irx1 *and *Irx3*, and this region may include the *CG10632 *gene.

As *Irx *genes are clustered in several species [e.g. [3,8,9,12,13]] and more than one *Irx *gene is observed in most bilaterian species, we wondered whether similar clustering may be found in all these species. We found these genes organized into clusters in most species (20 out of 28 bilaterian species; for the others, either there is a single gene, or the current state of the genome assembly does not allow to establish potential clusters due to very small genomic scaffolds; Figures 1 and 6; the data used to construct Figure 6 can be found in Additional file 4). The presence of clusters of *Irx *genes seems therefore to be an almost general rule in bilaterians, as previously suggested [12]. We also confirmed the observation made by Irimia et al. [12] that the *Irx *genes are associated in most bilaterian species except vertebrates, with a structurally and functionally unrelated gene known as *CG10632 *in *Drosophila *(Figure 6). *CG10632 *which encodes a well-conserved protein with Ankyrin repeats (Additional file 5), is found either 5' to the *Irx *cluster or within the cluster depending on the species analyzed (Figure 6). In vertebrates – as well as in the cnidarian *Nematostella *and the placozoan *Trichoplax *– putative orthologs are found (human Ankyrin repeat domain protein 43 and 56, for example), but are not physically linked to the *Irx *genes (not shown). This indicates therefore that (i) a cluster of one (or more) *Irx *and *CG10632 *genes was present in the last common ancestor of bilaterians, (ii) there has been a strong evolutionary pressure to maintain association of the *Irx *and *CG10632 *genes in bilaterians, (iii) this pressure has been relaxed in vertebrates. Further characterization of the *CG10632 *genes in species such as *Drosophila *and *Branchiostoma *is needed to define the mechanistic reasons for this association (such as regulatory region sharing).

These data about the genomic organization of the *Irx *genes can be interpreted in two different ways. The simplest and most parsimonious explanation is that a cluster of at least two *Irx *genes (+*CG10632*) is ancestral to bilaterians and has been conserved in this evolutionary lineage, like what has been observed for other homeobox gene clusters, such as the *Hox*, *ParaHox *and *NK *clusters [1,17]. This hypothesis is supported by our analyses that suggest the presence of at least 2 genes in the last common ancestor of each investigated lineage, lophotrochozoans, arthropods, and deuterostomes. One plausible and parsimonious interpretation of these analyses is that this situation might be ancestral to bilaterians. This view is, however, not supported by the phylogenetic analyses of the *Irx *genes at the scale of the bilaterians, which suggest independent gene duplications in the different bilaterian lineages (see previous sections). The hypothesis of cluster of *Irx *genes already present in the last common ancestor of bilaterians would require that we postulate that the phylogenetic trees do not show the real relationships between the *Irx *genes from the different bilaterian lineages, which might be a consequence of differential rates of evolution in these lineages. This hypothesis is also not supported by the presence of a single *Irx *gene in several different nematode species – we would have to postulate that one or more *Irx *genes have been lost in the nematode lineages.

The second possibility is that the duplications of the *Irx *genes have occurred independently and in all cases there have been pressures to maintain the physical linkage of the duplicated genes. This explanation already proposed by Irimia et al. [12] is in agreement with the phylogenetic analyses, but is faced with one major problem, explaining why in several independent lineages there have been similar pressures to keep the duplicated genes in clusters. Indeed, it is easy to explain that following tandem gene duplications there could be, in some rare cases, molecular events that lead to phenomenon such as shared regulatory regions or global gene regulation, favouring cluster maintenance, while in most other cases, the duplicated genes would be, after some time, dispersed in the genome, as it is observed for most multigenic families. It is much more difficult to understand why, in the case of the *Irx *genes, there would have been systematic events leading to cluster maintenance after numerous instances of gene duplications, unless postulating some particular properties of the *Irx *genomic region that would, by itself, favor the conservation of the physical link between the duplicated genes. The existence of an ancestral cluster of a single *Irx *gene and *CG10632 *may represent such a property, constraining the duplicated *Irx *genes to remain associated with *CG10632 *and therefore with each others. This remains nevertheless to be proven and does not explain everything, as for example why the *CG10632 *gene has never been duplicated while the *Irx *genes would have duplicated so many times (Figure 6, Additional file 5).

## Conclusion

We present here a large-scale phylogenomic analysis of the *Irx *and *Mkx *genes that extends a previously published study on the evolution of *Irx *genes in metazoans. Several main conclusions can be drawn from our study. First, an *Irx *cluster composed of two genes, *araucan/caupolican *and *mirror*, is ancestral to the crustaceans+insects clade and has been strongly conserved in this clade. Second, 3 *Irx *genes organized into a cluster were probably present in the last common ancestor of vertebrates and the duplication that has given rise to the six groups of genes (organized into 2 clusters) found in most vertebrates occurred in the gnathostome lineage after its separation from sea lampreys. Third, several *Irx *genes organized into clusters are found in many different bilaterian species representing various evolutionary lineages. This unexpected feature can be explained in two opposite ways. Our analyses were unable to discriminate between these two possibilities. The first possibility is that there was an ancestral *Irx *cluster composed of two or three genes in *Urbilateria*, and that there have been structural and/or functional constraints that maintained this organization in the various bilaterian lineages. However, the genes constituting these clusters would have a differential evolution which would hide their orthology relationships. Furthermore, additional genes duplications and losses would have also occurred in some lineages. The second possibility is that the different *Irx *clusters have been independently acquired, implying numerous independent tandem duplications and pressures to maintain the physical linkage of duplicated genes in several different lineages. In this view, the *Irx *genes would be specially prone to duplication events and/or retention of functional paralogs over long evolutionary times. With the currently available data, we do not think it is possible to favor one of the explanations over the other one.

## Methods

### Retrieval of the Irx and Mkx sequences

Irx and Mkx gene sequences were retrieved using TBLASTN and BLASTP algorithms [18] on the current assembly and the predicted proteins (if available) of the genomes of the species indicated in Figure 1, using the BLAST servers dedicated to these species (Doe Joint Genome Institute, Baylor College of Medicine, Flybase, Genome Sequencing Center, and Ensembl) or the National Center for Biotechnology Information (NCBI) BLAST server (Genomic BLAST databases) [19-24]. Additional BLAST searches were also performed against the NCBI protein and EST databases in order to identify *Irx *and *Mkx *genes in additional species whose genomes are not completely sequenced. Aminoacid sequences were subsequently predicted using Geneid, Genscan, and TBLASTN against the NCBI nr protein database [18,25,26]. All the sequences we have isolated are available upon request. Species abbreviations used in the present article are: *Acypis *= *Acyrthosiphon pisum *(pea aphid – insect); *Aedaeg *= *Aedes aegypti *(yellow fever mosquito – insect); *Ampque *= *Amphimedon queenslandica *(demosponge); *Anogam *= *Anopheles gambiae (*mosquito – insect); *Apimel *= *Apis mellifera *(honey bee – insect); *Bommor *= *Bombyx mori *(silkworm – insect); *Braflo *= *Branchiostoma floridae *(amphioxus – cephalochordate); *Brarer *= *Brachydanio rerio *(zebrafish – vertebrate); *Caeele *= *Caenorhabditis elegans *(nematode); *Calvic *= *Calliphora vicina *(Blue blowfly – insect); *Capsp1 *= *Capitella sp I *(annelid); *Culpipqui *= *Culex pipiens quinquefasciatus *(mosquito – insect); *Dappul *= *Daphnia pulex *(water flea – crustacean); *Dromel *= *Drosophila melanogaster *(fruitfly – insect); *Galgal *= *Gallus gallus *(chick – vertebrate); *Helera *= *Heliconius erato *(Red Passion Flower butterfly – insect); *Homsap *= *Homo sapiens *(vertebrate); *Hydmag *= *Hydra magnipapillata *(cnidarian); *Lotgig *= *Lottia gigantea *(limpet – mollusk); *Musmus *= *Mus musculus *(mouse – vertebrate); *Mytcal *= *Mytilus californianus *(mussel – mollusk); *Nasvit *= *Nasonia vitripennis *(parasitoid wasp – insect); *Nemvec *= *Nematostella vectensis *(sea anemone – cnidarian); *Pedhumcor *= *Pediculus humanus corporis *(human body lice – insect); *Petmar *= *Petromyzon marinus *(Sea lamprey – vertebrate); *Sackow *= *Saccoglossus kowalevskii *(hemichordate); *Schmed *= *Schmidtea mediterranea *(Planarian – platyhelminthes); *Spofru *= *Spodoptera frugiperda *(fall armyworm – insect); *Strpur *= *Strongylocentrotus purpuratus *(purple sea urchin – echinoderm); *Subdom *= *Suberites domuncula *(demosponge); *Tetnig *= *Tetraodon nigroviridis *(pufferfish – vertebrate); *Triadh *= *Trichoplax adhaerens*; *Tricas *= *Tribolium castaneum *(red flour beetle – insect); *Xentro *= *Xenopus tropicalis *(vertebrate).

### Phylogenetic analyses

Multiple alignments were performed with Clustal W [27] using the ClustalW web server at the Bioinformatics Center of the Kyoto University [28] and they were subsequently manually improved. Handling of the multiple alignments was done using SEAVIEW [29]. Unweighted maximum-parsimony (MP) and neighbour-joining (NJ) reconstructions were performed with the PAUP 4.0 program [30]. NJ analyses were done using the BioNJ algorithm [31] and 10,000 bootstrap replicates. MP analyses were performed with the following settings: heuristic search of over 250 bootstrap replicates; MAXTREES set at 3000, and other parameters set at default values. Maximum likelihood (ML) analyses were performed with PHYML [32]. PHYML analyses were performed using the WAG amino-acid substitution model [33], the frequencies of amino acids being estimated from the data set, and rate heterogeneity across sites being modelled by two rate categories (one constant and eight g-rates). The amino acid substitution model was chosen using ModelGenerator [34]. Statistical support for the different internal branches was assessed by bootstrap resampling (500 bootstrap replicates), as implemented in PHYML [32]. Bayesian inference was performed using the Markov chain Monte Carlo method as implemented in the MRBAYES (version 3) package [35,36]. We used the WAG substitution frequency matrix [33] with among-sites rate variation modelled by means of a discrete g distribution with four equally probable categories. Two independent Markov chains were run, each containing from 1,500,000 to 3,000,000 Monte Carlo steps (depending on the number of steps required to get chain convergence). One out of every 250 trees was saved. The trees obtained in the two runs were meshed and the first 25% of the trees were discarded as 'burnin'. Marginal probabilities at each internal branch were taken as a measure of statistical support. All the alignments and the trees are available upon request. Phylogenetic relationships between the species used in this study (as depicted in Figure 1) are based on [38-42].

## Authors' contributions

PK, AI, and MV retrieved the sequences, made the sequence alignments, and performed the phylogenetic analyses. PK analysed the genomic organization of the *Irx *genes. MV and DC participated in the design and coordination of the study. MV drafted the manuscript and all the authors participated in the editing of the manuscript. All the authors read and approved the final manuscript.

## Supplementary Material

Additional file 1 **List of all the sequences used in our study in fasta format**. The sequence of the proteins are given. For the Irx proteins, we highlighted the homeodomain and Iro box in red and blue, respectively. The name of the newly-identified proteins were underlined. Nucleotide sequences are available on request. Click here for file

Additional file 2 **Multiple alignments of the conserved domains of Irx and Mkx proteins**. These alignments only show the conserved domains of the Mkx and Irx proteins, as defined in [5]. Some of the newly-identified sequences are clearly incomplete, due to gap in the genome sequences and/or difficulties to predict compmete open reading frame from the genomic sequences. Click here for file

Additional file 3 **Phylogenetic analysis of *Irx *genes in arthropods**. The represented tree is a maximum-likelihood tree, based on an alignment of the full-lenght protein sequences and which has been rooted using the *Irx *gene from *Nematostella *as outgroup (this should be considered as arbitrary rooting). The most important monophyletic groups are indicated in red and the associated numbers are their statistical support values obtained with different methods of phylogenetic reconstruction, as described in Figure 2. Statistical support in the maximum-likelihood analysis for some other internal branches is indicated in black. Click here for file

Additional file 4 **Summary of the study of the genomic organization of the *Irx *genes and their physical association with *CG10632 *genes**. This table contains the genomic data that have allowed to construct Figure 6. Click here for file

Additional file 5 **Multiple alignments of sequences of the CG10632 proteins**. This file contains the alignment of the whole sequence of the CG10632 proteins. Click here for file

## References

[B1] Duboule D (2007). The rise and fall of Hox gene clusters. Development.

[B2] Cavodeassi F, Modolell J, Gómez-Skarmeta JL (2001). The *Iroquois *family of genes: from body building to neural patterning. Development.

[B3] Gómez-Skarmeta JL, Modolell J (2002). *Iroquois *genes: genomic organization and function in vertebrate neural development. Curr Opin Genet Dev.

[B4] Bürglin TR (1997). Analysis of TALE superclass homeobox genes (MEIS, PBC, KNOX, Iroquois, TGIF) reveals a novel domain conserved between plants and animals. Nucleic Acids Res.

[B5] Mukherjee K, Bürglin TR (2007). Comprehensive analysis of animal TALE homeobox genes: new conserved motifs and cases of accelerated evolution. J Mol Evol.

[B6] Anderson DM, Arredondo J, Hahn K, Valente G, Martin JF, Wilson-Rawls J, Rawls A (2006). *Mohawk *is a novel homeobox gene expressed in the developing mouse embryo. Dev Dyn.

[B7] Takeuchi JK, Bruneau BG (2007). *Irxl1*, a divergent Iroquois homeobox family transcription factor gene. Gene Expr Patterns.

[B8] Dildrop R, Rüther U (2004). Organization of *Iroquois *genes in fish. Dev Genes Evol.

[B9] Feijóo CG, Manzanares M, de la Calle-Mustienes E, Gómez-Skarmeta JL, Allende ML (2004). The *Irx *gene family in zebrafish: genomic structure, evolution and initial characterization of *irx5b*. Dev Genes Evol.

[B10] Peroviæ S, Schröder HC, Sudek S, Grebenjuk VA, Batel R, Stifaniæ M, Müller IM, Müller WE (2003). Expression of one sponge *Iroquois *homeobox gene in primmorphs from *Suberites domuncula *during canal formation. Evol Dev.

[B11] Larroux C, Luke GN, Koopman P, Rokhsar DS, Shimeld SM, Degnan BM (2008). Genesis and expansion of metazoan transcription factor gene classes. Mol Biol Evol.

[B12] Irimia M, Maeso I, Garcia-Fernàndez J (2008). Convergent evolution of clustering of Iroquois homeobox genes across metazoans. Mol Biol Evol.

[B13] Peters T, Dildrop R, Ausmeier K, Rüther U (2000). Organization of mouse *Iroquois *homeobox genes in two clusters suggests a conserved regulation and function in vertebrate development. Genome Res.

[B14] Bosse A, Stoykova A, Nieselt-Struwe K, Chowdhury K, Copeland NG, Jenkins NA, Gruss P (2000). Identification of a novel mouse *Iroquois *homeobox gene, *Irx5*, and chromosomal localisation of all members of the mouse *Iroquois *gene family. Dev Dyn.

[B15] Taylor JS, Peer Y Van de, Braasch I, Meyer A (2001). Comparative genomics provides evidence for an ancient genome duplication event in fish. Philos Trans R Soc Lond B Biol Sci.

[B16] Aboobaker AA, Blaxter ML (2003). Hox Gene Loss during Dynamic Evolution of the Nematode Cluster. Curr Biol.

[B17] Butts T, Holland PW, Ferrier DE (2008). The Urbilaterian Super-Hox cluster. Trends Genet.

[B18] Altschul SF, Madden TL, Schaffer AA, Zhang J, Zhang Z, Miller W, Lipman DJ (1997). Gapped BLAST and PSIBLAST: a new generation of protein database search programs. Nucleic Acids Res.

[B19] The DOE Joint genome institute genome portal. http://genome.jgi-psf.org/.

[B20] The Human Genome Sequencing Center, Baylor College of Medicine. http://www.hgsc.bcm.tmc.edu/.

[B21] FlyBase: a database for *Drosophila *genetics and molecular biology. http://flybase.org/.

[B22] The Genome Sequencing center – Washington University of Medicine. http://genome.wustl.edu/genome_group_index.cgi.

[B23] The Ensembl Genome Browser. http://www.ensembl.org/index.html.

[B24] The National Center for Biotechnology Information. http://www.ncbi.nlm.nih.gov/.

[B25] Parra G, Blanco E, Guigo R (2000). GeneID in *Drosophila*. Genome Res.

[B26] Burge C, Karlin S (1997). Prediction of complete gene structures in human genomic DNA. J Mol Biol.

[B27] Thompson JD, Higgins JD, Gibson TJ (1994). CLUSTALW: improving the sensitivity of progressive multiple sequence alignment through sequence weighting, position-specific gap penalties and weight matrix choice. Nucleic Acids Res.

[B28] The Bioinformatics Center of the Kyoto University. http://align.genome.jp/.

[B29] Galtier N, Gouy M, Gautier C (1996). SEAVIEW and PHYLO_WIN: two graphic tools for sequence alignment and molecular phylogeny. Comput Appl Biosci.

[B30] Swofford DL (1998). PAUP: Phylogenetic Analysis Using Parsimony (and Other Methods), Version 4.

[B31] Gascuel O (1997). BIONJ: an improved version of the NJ algorithm based on a simple model of sequence data. Mol Biol Evol.

[B32] Guindon S, Gascuel O (2003). A simple, fast, and accurate algorithm to estimate large phylogenies by maximum likelihood. Syst Biol.

[B33] Whelan S, Goldman N (2001). A general empirical model of protein evolution derived from multiple protein families using a maximum-likelihood approach. Mol Biol Evol.

[B34] Keane TM, Creevey CJ, Pentony MM, Naughton TJ, Mclnerney JO (2006). Assessment of methods for amino acid matrix selection and their use on empirical data shows that ad hoc assumptions for choice of matrix are not justified. BMC Evol Biol.

[B35] Huelsenbeck JP, Ronquist F (2001). MRBAYES: Bayesian inference of phylogenetic trees. Bioinformatics.

[B36] Ronquist F, Huelsenbeck JP (2003). MrBayes 3: Bayesian phylogenetic inference under mixed models. Bioinformatics.

[B37] Wada S, Tokuoka M, Shoguchi E, Kobayashi K, Di Gregorio A, Spagnuolo A, Branno M, Kohara Y, Rokhsar D, Levine M, Saiga H, Satoh N, Satou Y (2003). A genomewide survey of developmentally relevant genes in *Ciona intestinalis*. II. Genes for homeobox transcription factors. Dev Genes Evol.

[B38] Adoutte A, Balavoine G, Lartillot N, Lespinet O, Prud'homme B, de Rosa R (2000). The new animal phylogeny: reliability and implications. Proc Natl Acad Sci USA.

[B39] Delsuc F, Brinkmann H, Chourrout D, Philippe H (2006). Tunicates and not cephalochordates are the closest living relatives of vertebrates. Nature.

[B40] Dunn CW, Hejnol A, Matus DQ, Pang K, Browne WE, Smith SA, Seaver E, Rouse GW, Obst M, Edgecombe GD, Sørensen MV, Haddock SH, Schmidt-Rhaesa A, Okusu A, Kristensen RM, Wheeler WC, Martindale MQ, Giribet G (2008). Broad phylogenomic sampling improves resolution of the animal tree of life. Nature.

[B41] Savard J, Tautz D, Richards S, Weinstock GM, Gibbs RA, Werren JH, Tettelin H, Lercher MJ (2006). Phylogenomic analysis reveals bees and wasps (Hymenoptera) at the base of the radiation of Holometabolous insects. Genome Res.

[B42] Blaxter ML, De Ley P, Garey JR, Liu LX, Scheldeman P, Vierstraete A, Vanfleteren JR, Mackey LY, Dorris M, Frisse LM, Vida JT, Thomas WK (1998). A molecular evolutionary framework for the phylum Nematoda. Nature.

