# Supplementary material for: Evolutionary history of the iroquois/Irx genes in metazoans
Source: BMC Evol Biol. 2009 Apr 15;9:74. doi: 10.1186/1471-2148-9-74 (PMC2674049; doi:10.1186/1471-2148-9-74)
Supplement: Additional file 1 — List of all the sequences used in our study in fasta format. The sequence of the proteins are given. For the Irx proteins, we highlighted the homeodomain and Iro box in red and blue, respectively. The name of the newly-identified proteins were underlined. Nucleotide sequences are available on request. [file 1471-2148-9-74-S1.pdf]

IRX PROTEINS

Drosophila melanogaster

>mirr_Dromel
MTVHSNETGICLVMNAVSTHLATSSPNTPPTNAPQSPPSTLPTLACPAAQPLSAPGIAGPGH
SGMVTSTVPPGARSTSPCGPSAGLPALSLVGAPPPGPHSSAPGGAPAPGAGNPPNRCCDTGR
TIYTDPVSGQTICSCQYDMLNYQRLAAAGGVPLGVYPEGMSAYLSGIAADQPPFYANPAGID
LKENLVAGASPWPYPSMYHPYDAAFAGYPFNSYGMDLNGARRKNATRETTSTLKAWLNEHKK
NPYPTKGEKIMLAIITKMTLTQVSTWFANARRRLKKENKMTWEPRNRVDDDDANIDDDDDKN
TEDNDLLDAKDSGVGSTDDKDRSGRLGDMMTDRPGESNNSEWSESRPGSPNGSPDLYDRPGS
MPPGAHPLFHPAALHHHFRPPAGSPPDIAAYHHHQQQLLQQHQQAQQNSLQTAVGGTAKPRI
WSLADMASKDSKDSSSGAKDNHPELPPAHPGFYGHPGQQPSPGKILSPLAARIPNYSPYVRP
DLYRGFYGPAAAHLGAPTQEFLEHQRTFGASLAAHNGPLGMNPLLWKAAVSGAANGHHFAPL
SLTTSGGSSGGQQVAPPPVASPSASSSSSSMGCDVVHIPTSSGQSAAQHMMGPISSNSTASS
SSSHSGKISPGVNVTSLSAKP
>ara_Dromel
MAAYTQFGYGGFPSASQLLPPSVQTTEDASANVNVNVNEALVMTNAPAMSPTGGQDCQGSQP
SGGAGGDASSGALSPNALSQNSNAATVVGAGGGSSAGGGGPADLATGGSLDGNGVGTTPTAG
GAGGGGSCCENGRPIMTDPVSGQTVCSCQYDSARLALSSYSRLPAASVGVYGTPYPSTDQNP
YQSIGVDSSAFYSPLSNPYGLKDTGAGPEMGAWTSAGLQPTTGYYSYDPMSAYGGLLVSNSS
YGASYDLAARRKNATRESTATLKAWLNEHKKNPYPTKGEKIMLAIITKMTLTQVSTWFANAR
RRLKKENKMTWEPKNRTDDDDDALVSDDEKDKEDLEPSKGSQGSVSLAKDETKEEEDAIDED
QKCLGQANILRAGFGYPSAGSGSGGYPGGGGSSSGHPGGYHPYHHQHPAYYQAGQQGGMLPF
HGENSKLQTDLGDPKNQLGRDCGVPIPATKPKIWSLADTVGCKTPPPAYMGHQSMPLQQQQQ
QQQQQQQAQHQYPPSEAGRDQQLFNGAAAPYLRPHTTAYGGFLGATTQQLHTTNNSIPYSNM
PPQQQQPQQQQQQLQQGGTIHTTGSSSGPIIPLQFHNRHPQQQQQLQQQSQSTASQRAMGFL
EAQPDTPPQTPPNMKVLSGALSLLPTATQVPMTATCRSSNAFGFPASGYPMNFSARLGEYSP
RDDYSSGNSSSSSSSSPQLQRNEAMFKPLFKKFTN
>caup_Dromel
MAAYAQFGYAGYPTANQLTTANTDSQSGHGGGSPLSGTNEASLSPSGGSTATGLTAGPLSPG
AVSQSSHHAGHKGLSTSPAEDVVGGDVPVGLSSAAQDLPSRGSCCENGRPIITDPVSGQTVC
SCQYDPARLAIGGYSRMALPSGGVGVGVYGGPYPSNEQNPYPSIGVDNSAFYAPLSNPYGIK
DTSPSTEMSAWTSASLQSTTGYYSYDPTLAAYGYGPNYDLAARRKNATRESTATLKAWLSEH
KKNPYPTKGEKIMLAIITKMTLTQVSTWFANARRRLKKENKMTWEPKNKTEDDDDGMMSDDE
KEKDAGDGGKLSTEAFDPGNQLIKSELGKAEKEVDSSGDQKLDLDREPHNLVAMRGLAPYAT
PPGAHPMHAAYSSYAQSHNTHTHPHPQQMQHHQQQQQQQQNQQQLQHHQMDQPYYHPGGYGQ
EESGEFAAQKNPLSRDCGIPVPASKPKIWSVADTAACKTPPPTAAYLGQNFYPPSSADQQLP
HQPLQQHQQQQLQQLQQQQQHHHHPHHHHPHHSMELGSPLSMMSSYAGGSPYSRIPTAYTEA
MGMHLPSSSSSSSSTGKLPPTHIHPAPQRVGFPEIQPDTPPQTPPTMKLNSSGGSSSSSGSS
HSSSMHSVTPVTVASMVNILYSNTDSGYGHGHSHGHGHGHGHGLGHGHGLGHGHGHMGVTSN
AYLTEGGRSGS

Anopheles gambiae

>irx1_Anogam
MIETVIGERRSSGFAVAGEEKTMSHFPFRGSPSAQSTTPTVPSAPSSPPTAPVAMAVTTVPP
SARSASPCSATSGPPTSGPGGAAGSGGSSGGRCCDTGRPIFTDPISGQTVCSCQYDLISYQR
LASAGIAGPGGVPLSMYSAPYSPETMAAYFPAIGADQAPFYANPAAAGIDLKENLAAGGAPW
PYPSVYHPYDAAFGYPFNSYGMDLNGARRKNATRETTSTLKAWLNEHKKNPYPTKGEKIMLA
IITKMTLTQVSTWFANARRRLKKENKMTWEPRNRVEDDDVNLDDDDDDNKSLKDDKEILDSK
DSGTGSSEDGDRPSRLDMLDRPGGSEWNGSRNGSGPNSPDIYDRGVPGHHHHPLLHPSNFPP
HLRPAISTPPDVNHTPSGSTNPSTKPRIWSLADMASKESKDSDSSSPTAGGYPSPGKMPVTP

LAGRGLNPLHHHPYMRPDFYRNLYGHHLAPGSPEVSLLESYQRTFGATIGAHNGLPVSISPL
ITKASGSGPPFAPLSLTTANHHLHQQQHPHTAPPPGASPASSTSSNGPENLTPTSVSLPSGT
GPNEVKLINVLANSAKP
>irx2_Anogam
MQGGSSLGSSAVLSLLVSGGQTTTASSQSPISATNAPSLSPGGSATGAPSGALSPGAVSQTS
QNNASLGGGGGGGGGGGGGGGGGPTTSCCENGRPIMTDPVSGQTVCSCQYDSARLALSGYSR
IPTGSVGVYGTPYPSTEQNPYPSIGVDSSAFYSPLSNPYALKDSGPTSDMTAWTSAGLQPTT
GYYPYDPTLAAYGYGAGYDLAARRKNATRESTATLKAWLNEHKKNPYPTKGEKIMLAIITKM
TLTQVSTWFANARRRLKKENKMTWEPKNKTDDDDDAMVSDDEKDKDDLDPDKGGRDHKGKRT
EDSSRNVMLFLLASLHCAHRARDGRIGRACIVLLAAVGVSGQFLRHNGQPNGKTAEQHRNDT
RTQCQSCPHSCLHGKSVSSGDMDHSKNQTGSDCGVPIPASKPKIWSLADTAACKTPPPHHLP
LHLQHHHHQHHPHQQQQQQLQHPHQHHPQQQQQSHPVQGLHGSWMTGTSVDYHPNQSQQAAH
HLQQQHLQQQSSQQTQHLLQQQQQQHHVQHQGNSNGANSMMMIGDAGSPMSMSMGNTTTNNS
SSSNTPPNSQGMGGSMGNGMMSSFGSTPYSRYGGFLAGAQHYNTTLSSSSNSSNSSNTSNHS
STTGPVLNHPTTPTLQQQQQHQGNNLQQQQQQQQSQSMNSTPSQQQQQSMGFPEVQTDTPPQ
TPPNMKLPSMAANLLTTPTTGTCYSSTNGSTPNTASGNGSSSNNNNNNNNPSTANNNAGGSS
SGYASSPDSMSQQGTGGYYRMQQQQQEQPDGFKPFFKK

Aedes aegypti
>irx1_Aedaeg
MIETVTNSERRSSIPGGEKTMSHFPFRGSPTTQSTTPSAPSAPSSPPAAPVAMAVTTTVPPG
ARSTSPCSATSGPPTSGSGRCCDTGRPIFTDPISGQTVCSCQYDLINYQRLASAGIAGPGGV
PLSMYSAPYSPETMAAYFPAIGADQAPFYANPAAAGIDLKENLAAGGAPWPYPSVYHPYDAA
FGYPFNSYGMDLNGARRKNATRETTSTLKAWLNEHKKNPYPTKGEKIMLAIITKMTLTQVST
WFANARRRLKKENKMTWEPRNRVEDDDVNLDDDDDDNKSLKDDKEILDSKDSGTGSSEDGDR
PSRLDMLDRPGNSEWNGSRNGSGPNSPDIYDRPIHGHPLLHPSNFPPHLRSTIGTPPEVNHT
PSGSTNPSKPRIWSLADMASKENKDSDSSSPTAGGYPSPGKIIAPLAGRGINPLHHHPYMRP
EFYRNLYGPHLTPGSPEVSLLESYQRTFGATLAHNGIPVGMNPLISKASGSGPPFAPLSLTT
SSNPTTAPQPGASPASSTSSGPENLTPSSPANEKLISVTNASKP
>irx2_Aedaeg
MEISLTSLGIESIIVPATRYTNVKMATLAKKLSVNNCRSAEKNTKLRSRLCPTKLIITFLAT
LLWSIVVYASCRCREVHRVAISECECSPTVNRTEAVFILGIRLCDFPSSPDGCRARRLSSSC
VASTCAQNDVKEESCRSEHFLKEFVLISDWSYREAYEYGDSNDWDRGSNNTARRKNATREST
ATLKAWLNEHKKNPYPTKGEKIMLAIITKMTLTQVSTWFANARRRLKKENKMTWEPKNKTDD
DDDAMVSDDEKDKDDMDPDKSRDHKVQGHPENHCIFKCGKERGRGSLFFRVVSVTASALFWS
YFVASSMCEQVNVSRRLFFRIPYFRLPNFRGEKKGSTTLRDSSLPVASSHVSFRLVGRIAYP
LCELLIQFEYYEIQEDNLRRSSHISSWILMFDRQWLVASAFMARKGTMDHGRLAGIFN

Nasonia vitripennis
>irx1_Nasvit
MSQFSFRGSPSLQCPVTVSSSLTSSSASPVSGLLSSASATPGSTTSGGTTTAHHQAPQQPHH
PLGVRMAVTSAVVRPPGGSPTAVASVSPASQTSGGPPTAPQPASSGPTPGRCCDTGRPIFTD
PITGQTVCSCQYELLGGYQRLGALPTAALSMYSAPYAAAAAAAASEGMAAYFPGLGAEQAPF
YTPTAAGLDLKENLGAGAAAAWPYPSVYHPYDAAFASYPFNGYGMDLNGARRKNATRETTST
LKAWLNEHKKNPYPTKGEKIMLAIITKMTLTQVSTWFANARRRLKKENKMTWEPRNRVEDED
NNNDDDDSGRKSVDEKDRLDSKDSGTGSSEDGDRGTSDRHRLDLLHGGGVGGPGGAGRAESE
WSESRADSGPDSPECLYDQREPPSRHHHPTLQLQHHPAFMAAAAAASPHHHARLMQRHPSPE
NTSPGAHHLHHPGVIPSSTSTSATTASATTKPRIWSLADMASKDGDQPSPNSNILGSPYNSN
SGGNGGGKIVSPLASRLPPHHPLHPAMHPGSQFVRPHPDFYRNFYGGPHLGSGDMSLLETYQ
RTLGGLGGVMPPTSAPGILASSSASSTSANLKSHFSINGSGAGAGPGGPSVLLTTASSGVSP
SASSTASSNGSDQSPGGGLAPSELKSPGRV

>irx2_Nasvit
MSEDCWAEHVGLLQDVPGATDQQQHHPQPPPPVPDFLMLPHSHHHELLVSGGQPTTATSPSM
SSAGALSPGALSPSSTATTATTTGGGSSTTTGGASTPVGAAGGGTTTAAASATSSGPGCCEN
GRPMMTDPVTGQTVCSCQYDSAARLALGAYPRLGPGATSYSSYPTPTPSGSDQGPYPSIGMD
SSAFYSPLGNPYGLKDATGMGMTADMGAAWGTAALQPAATGYYPYDPTLAAYGYGAGYDLAA
RRKNATRESTATLKAWLNEHKKNPYPTKGEKIMLAIITKMTLTQVSTWFANARRRLKKENKM
TWEPKNKTDDDDDAVLTDSEDNKDKDDMGSDNRQDRVGDDARRGLDDNEPMRHVKAEHMQHD
KDLDDEDDLDLEDDPRRVGEHPYHHSMHHHHHHHHHQGYGPDDHLKDESLKSDCNAAGVPIP
ATKPKIWSLADTAACKTPPPPSHPHHHQYQLHHQHQQQQHSPGWEVVGVVEPTWAPPAVGGL
SGFALPSSASMSPSSAATAPYSSTGARYGGFLSSSSGGQLHYNPNSSSSSSASSSAAAGFPE
VGTDTPPQTPPNMKVTTATPNGVIQGPPAGYCPGGNSNTPSNGPVGSANTGHYVAAATAPSH
AAATVGGYMQQSQANGGVGAGAGGFSTRLQNSPHKDFAPASAQNSILHQHQTTASLPPTEAT
TAFKPFYKGTQSMGSGFVSPV

Tribolium castaneum
>irx1_Tricas
MSQFGFRASPNTQSAVSSGPSQPSPPPSSMAVTSSAPTPGSPPAPSQRCCDTGRPLFTDPLT
GQSVCSCQYELLGYQRLAGGVPGLPALSMYSAPYPEGMAAYFPALGADQAPFYTSTAAGLEL
KENLAAGAATWPYPSVYHPYDTAFAGYPFNGYGMDLNGARRKNATRETTSTLKAWLNEHKKN
PYPTKGEKIMLAIITKMTLTQVSTWFANARRRLKKENKMTWEPRNRVEDEDNNNDDDDHKST
DGKDILDSKDSGTASSEDGDRPPHSRLAPDTASEWSESRPDSGPDSPECYERPPHPAFLPPR
SSGSPPQISASVSSKPRIWSLADMASKESDGPPPSTASSLYSTAASRLRLPPPGLHTAPYAR
PHDFYRSLYGPGMAGSGSPDASLLETYSRTLAGQSVLTKANLATATSIASNGPLGLTTSSRL
SPSSTSSLSSGSETPLKPVALNKA
>irx2_Tricas
MEDGWESHVSPLVMGVGMGDMQGMGEMQTAASCSGHGQEMPPEMNMPHHQELVASHPELNPG
IHQEIEQELGREMGRNDMRTHQDAMGGHDDYFAHQLLVSGQPTTNSGSSPASGGGSLSPGGS
AGVPGTLSPGGTSSTGPTQCCESGRPLVTDPVTGQSVCSCQYDPARLAALQYPRSVGVYGAP
YPSTDQNPYPSIGVESSAFYSPLSNPYALKDAGGTAADMSAWTSAGLQPTTGYYPYDPALAA
YGYGAGYDLAARRKNATRESTATLKAWLNEHKKNPYPTKGEKIMLAIITKMTLTQVSTWFAN
ARRRLKKENKMTWEPKNKTDDDDDALVSDSDDKEKDEEDKRDDTDMHGRIKSERNGKDLDDD
DMNDDDRKDDLLGNNMHHILGNTFGLKPEMKTSDCGVPLPPSKPKIWSLADTAACKTPPPPP
PQQQQWLGSNGFALPSSRYAGSGFLPSHCQQGFPEVQTDTPPQTPPSMKLPSVAGNLPCIGT
AAATAANSGYSQSGFLGGFGRLQSPHRPPPPQAQMTAPTTDSAAFKPFYKRWMVAGGPPSPE
PMGKVQQIQIVWSKGASSGGHFLPTTGGRHGNSSKNATVVVQFTSKTWNTVFTDQMGNGLGE
TGGRSLVLFNPPPARPPEGKNAPQEGTEKEDSSETPTAAPSHCPTPLHPLGRLAKIRPKEAQ
ADRNYHLVHRRYGSGATGFFATRTRGAS

Apis mellifera
>irx1_Apimel
MAKTQFEPGSWCPFPVTNIPRCTAWGVAMIHRSRCTAFMPDRMSVGRIDIVQGDKVCRSSVC
NVDRDKDSVRLCSGCVPHSKGFNESVVNDNEITHGTTCPVTVSSSLTSSSASPVSGAILSAG
GSSLVSVASTATNHPLGVAAGLSNARMAVTSAVVRPPGSPTTSVSPSQGHPPPTTGGPTPTG
RCCDTGRPIFTDPLTGQTVCSCQYELIGGYQRLGALPTAALSMYSAPYAAAAAAAASEGMAA
YFPSLGAEQAPFYTPTAAGLDLKAENLGAGAAAAWPYPSVYHPYDAAFASYPFNGYGMDLNG
ARRKNATRETTSTLKAWLNDHKKNPYPTKGEKIMLAIITKMTLTQVSTWFANARRRLKKENK
MTWEPRNRVEDEDNNNEDDDSGRKSVDEKDRLDSKDSGTGSSEDGERPAHRMDLLGTSGGST
GVQGRTESEWSESRADSGPDSPECLYDQREPPRHPLQLQHPAYLASSHGRLLRHPSPESTPP
SSHHVPPSTTTASSTGSGVTTKPRIWSLADMASKDGEQQSPIPTTMTGLSSPYGGSGGAGGG
VAGGGGGGGKLMSPLASRLPPHHPLAMHSGAQFVRHHPDFYRNLYGASHLGSGDMSLLETYS
RTLGGLSGVMPPSTAPSILTSSASAVSASGKPFSINGGSTAPGGAVLLTTATSGLSPSSSST

ASSGGSDQSPHPSLSSTQELKSPGRV
>irx2_Apimel
MDISEDCWEHVGLLQEVSAAEPPPPPPAPDFLIQQQELLVSGGQPTTATSPAMSSGGALSPG
ALSPSSTATTTGAAAAAASGGASTPVAGATGPGCCENGRPMMTDPVTGQSVCSCQYDSAARL
ALGAYPRLAPTATSYSSYPTPTPSTTDQGPYPSIGMDSSAFYPPLGNPYGLKDTTGMGMTAD
MGAAWGTAALQPAATGYYPYDPTLAAYGYGAGYDLAARRKNATRESTATLKAWLNEHKKNPY
PTKGEKIMLAIITKMTLTQVSTWFANARRRLKKENKMTWEPKNKTDDDDDAVLTDSEDNKEK
DDLAGDNQGDRVGEEARRGLEENEPMRHVKAEHLQHDKDLDEEDDLDLEEDPRRGEHPFHHA
MQHHHHHHQAYPGEDHLKDEGIVKTDCSAAGVPIPATKPKIWSLADTAACKTPPPPTHPHHQ
QYHHLHHHQQHYGQQQQQQQQQHQHHHMPSQGHHHHSQQPWLGGAGGGGNLSSFALPSSASM
SPSAAATAPYSSAATRYGGFLSSSSGGQLHYNPNSSSGSSSSASSSAAAGFPEVGTDTPPQT
PPNMKVATPNGVIQAPTAGYCTGNGNGNAASNGNPNPYGQSHPAAAGYLSTSSGSASSGSSF
SSRLQASPHKDYSPAGQNSILHQHQTTASLPPTEATTAFKPFYKGSQSMGSGFVSPV

Bombyx mori
>irx1_Bommor
MDLNGARRKNATRETTSTLKAWLNEHKKNPYPTKGEKIMLAIITKMTLTQVSTWFANARRRL
KKENKMTWEPRNQDGKDSGTGSSEDGDRPQQRLDLLGQRTESEWSESRADSGPESPEPYERP
IHPAYQHLPSRAPPGSTPASAKPRIWSLADMASKDGESPAPPSSASAFYQSAAAAAAAARLA
HPYGRPELYRGLYPPTHPADVALLEYSRSLALANPAPAPPAPSPSSSSTSSVAEPPPPPRA
>irx2_Bommor
MAQFLHQLPPMLPAFGLLNGARDAGVSGLLDNVRPPAWQSSSTSLCFHSNPYALKESGGEMS
AWTSAGLQPSGGYYPYDPTLAAYGYGAGYDLAARRKNATRESTATLKAWLNEHKKNPYPTKG
EKIMLAIITKMTLTQVSTWFANARRRLKKENKMTWEPKNKADDDDDTMLSDEEKDKDDQDDK
LKSHKVKWAAPRPKFHITRLPFVGRTGCHAEVAFGLSDELPYFMFLPKDLSSSWIVSFMRIF
RRKVNRFDSISRNRYVECNHKIQTRIRHARTRCEHNINPKRVNGKRVERTTAAIDVPIRSDL
TKTFAPIKFPHILFIIAPPAIGAELIHITWNRCIHRQCVSKDLFCHVPSGFGMNSPPLCFPS
AMTCPSSNEACGEYFMEGSGLALPLALLTSMSDGDHLPSGGPYARLPTKAIKNVRHETLDNF
KGSTTKIHKREKRIHQRTSCYYFHDSSLRYVFVVTSINRRFAYSGIGPDEERKGDEMLQGMH
HQLLSYGIKEESKRTSDCGVPIPASKPKIWSLADTAACKTPPPAAQPWPQHSYGPPERAAPI
DGSSNGFALPATAAASPASGSYGRYGGFPGSQYSGQHPCVHPAAFPDVQTDTPPQTPPNMKV
PSVANPLGSGGGSGYCFPRHQQSPQRDPYHNPHHAPTNHQPNQPHHNEGSAAFKPFYKR

Pediculus humanus corporis
>irx1_Pedhumcor
MDLNGARRKNATRETTSTLKAWLNEHKKNPYPTKGEKIMLAIITKMTLTQVSTWFANARRRL
KKENKMTWEPRNRVEEEDNNNEESGRKSADNGKDPDSKDSGTGSSENGERPNHRMELLDRPC
GPVDTASEWSESRADSGPDSPECLFERQQQLLHPAYQHLQHPASRLHVPSSSSPSGPPKPRI
WSLADMASKENDIHSSPSTTSAFYATAAGKLISPLSRGAMPPLSVPPGSYPRPPPHEFYRSL
YGPAAHLAGSAASESVSLLETYSRTFGGGLEGGGPPAAGPGGQLSIVLSKVAAAAAATSGNP
TFNLNGTRPTQSVATGASSASSSTSSSASDHQQPLLPNKQIPDHLKTPAGKP
>irx2_Pedhumcor
XYSAGYDLAARRKNATRESTATLKAWLNEHKKNPYPTKGEKIMLAIITKMTLTQVSTWFANA
RRRLKKENKMTWEPKNKTDDDDDAIVSDTDDKDKDDLSYDSEKVKDRLREVDKDSMHHVKME
NRIIDKDLDDDDETDEDDRKPDGLTLTHHHIHHHHNHMHHSIMGGMKEEMKIQDCGIPIPAT
KPKIWSLADTAACKTPPPSQQQSWLMPNNGSGSGPMMGLNSFALPSATSSSAAAAAASYSRY
SGFLGHYNGNNHSTMGNYNTPSGFPEVQTDTPPQTPPNMKLPSVANNIINSNSCFGSNNNNT
TSHHHNQQHQHQHQQQQQQQQPQSTSYHHNVFVNNFNRMQSNQQQQQQQQQLSPQKDRHNDY
QHQQQQTMRTTTGNPQHQHPVNPNEGTTAFKPFFKK

Acyrthosiphon pisum

>irx1_Acypis
TTSLVQPLPQPPHTSSSSSSSPPPLQLSLTAARRHTSVSPQPPSSTAAGHNSSPPPPHQSRS
TVGGSTTASAALPPSSTAPSSVGGSPTTAVATVSTSTAAAAAAAPPRCCDTGRPIFTDPITG
QTVCSCQYDLLSYQRLAASGVPALSMYTAPYPEGMAAYFPALGSDQPPFYSAAAAGLDLKEN
LAAGATWPYHSVYHPYDAAFGYPFNGYGMDLNGARRKNATRETTSTLKAWLNEHKKNPYPTK
GEKIMLAIITKMTLTQVSTWFANARRRLKKENKMTWEPRNRVEDEDNNNEDDRKSPDPKDLD
CKDSGTGSSEDGDRPGANHTGSEWSESRADSGPDSPELFDRPPPPHFLHHPPPYHHGHYPGA
PHHPPPSHLGHHHHHHHPSAAPPHHRFASVSPPTTGGVGGGLLPSQSSAPKPRIWSLADMAS
KDTTTTTTAAAAVETSTPSTPPPPPSPHQHQRSPHHQQLSPPSVGGASSAGHRRPPAVPVPS
LPAGYARHEQLYRSFYEDPAAAAYHARAFGGGGGIVFQPSAAHQHHLHHHHQQQQLHSAAAA
VAEPSPASSSSDAESVVAVQHAERA
>irx2_Acypis
SAMTAESVSPPLAAGGGGGGLSPDVMGAAGCGTCCTAATVGGVTGDRNGGSESAVAVCSCHL
AAAASLPPHYASHRSTPATTAVYAPYPSTDQNPYPSIDTSSFYPHLSNPYGLKDSTGSSDMW
SSASLQPSTGYYHYDSTLAAYGYSAGYDLAARRKNATRESTATLKSWLNEHKKNPYPTKGEK
IMLAIITKMTLTQVSTWFANARRRLKKENKMTWEPKNKTDDDDDDDAGSSDCEDKDKDDMLM
GDEKHCNKDVRKDSHGDHMMGQGVMSDGDDDRKPMLQHLYHPDQMHHMKVHHHHVMQDDHHQ
DCGIPLPPTKPKIWSLADTAASKTPPPGHQWPNVISPSLASPGFTMPNTSSLSPSGSSYSRY
GANGFYGSGYNGGNSNSSSSSAQQMAGYPDVQTDTPPQTPPNMKLPLSASSGQQQQQQFGGH
QGTYNGYHHQQQQQHQQQQHNSYHHRQHVPPIQQQQQHDVVRSAQEVQQQQQQQQQQQYHQQ
HVALSQHNNNNNNNSNTNNNNHQMAVVANDASTAFKPFYKTPSQQPHQQPHQQQHQQQQHRQM
NGYVSPV*

Culex pipiens quinquefasciatus
>irx1_Culpipqui
MENLERSRLSYCSPMSSNGSTLEKTTFNDFSYIIHLASPFEAMLAPSVGYPTVVLLERRKLR
KLLRLLEYIRTGNKLVTPEGNMLLFRLVVSAPSLSPTRLLSEPYHDQNRMAPKALHQAPQLT
PPRSYGMDLNGARRKNATRETTSTLKAWLNEHKKNPYPTKGEKIMLAIITKMTLTQVSTWFA
NARRRLKKENKMTWEPRNRVEDDDVNLDDDDDDNKSLKDDKEILDSKDSGTGSSEDGDRPSR
LDMLDRPGHSDWNGSRNGSGPNSPDIYDRPMPGHPLLHPSHFPVHLRSTIGAHPDVSHTPSG
STNPSKPRIWSLADMASKENKDSDSSHSTGYPSPGKIIAPLAGRGLNPLHHHPYMRPEFYRN
LYGPHLTPGSPEVSLLESYQRTFGATLAHNGIPVGINPLISKASGSGPPFAPLSLTTSGNPH
VAPQPGASPASSTSSGPENLTPSSPAAEKLISVANSSKP
>irx2_Culpipqui
MQSSLSRMRETTSQFVQVVHDTHTPCHGGCLDGGNSSTTPYYRQTTTPATKSETARRKNATR
ESTATLKAWLNEHKKNPYPTKGEKIMLAIITKMTLTQVSTWFANARRRLKKENKMTWEPKNK
TDDDDDAMVSDDEKDKDDMDPDKSRDHKDMHHHHVKAEHDKEDDDDDLTDDERKPENLMGHP
GHLGQHHPYYHHSMLGGHHHHAGLKSELDHPKNQTGSDCGVPIPASKPKIWSLADTAACKTP
PPHSALHSSWMTGGYHPGQHHQNQQGQVNQQQQQHQHPHHLQQHPASGMMMGGESSPMSMNM
ANTAHSQGMGTMGSGMMNNFVNTPYSRHGGFLTGAQHYNTTHNNNTNSNTSTGQVTDATTPT
LQQQQQPPQQQQQHPLQGMHQQHQQQQLQQSQQPMNSNNSQSMGFPEVQTDTPPQTPPNMKL
PSVAGNLLTVSTTPTTGTCFNNGQNSAGSINNNNNNLTPNNNNLNQANGYASSPESMGQSSQ
QGQNNYYRMQSNSPQKSQQDYSQQMLHQPDGFKPFYKK

Daphnia pulex
>irx1_Dappul
DDRSLRLMAASASAAAVSTTTPIVLSSPRTPPTTLTASSGCCCDGSRPLVPDPLTGQSICAC
QLESGSATSTAAMLLGLPRLSGSLAGSTTTTTTSASPGATSALGLGLSLYHPYHQHQQQQQQ
QQQQHDLVHQHQQAIAAAHQQQQQQPFLTPDQSSFYAAANAGHDLKDSALAFNGAAAAAAAA
AGWPYPSMYYPYDPAMAAYPFGGYGMDLNGSRRKNATRETTATLKAWLSEHKKNPYPTKGEK
IMLAIITKMTLTQVSTWFANARRRLKKENKMTWEPKNRNDDGDDDDDGDSSDAEPSKDHHKR

SSSDGRDGVLSSADSNAAHHQRAWNGIGSAGSGHHLVMDDHHHQQLHRLHQHHRHHRDSEDN
IDVMLDSDEEELDLLSSGGDSACLSPPADHHHHLPGSHHLGNHLGNHHHLGLGVASTGVPHS
SSSSTTTTSTNSSKPKIWSLADMAGSQPSHQPPPASGSPHTTAAAALIQQQQQQQQQQQQQQ
QAAAAAAVAAAAVFPLPFGPLSQAFGLRSPHSSFLPPNNNNNTTPGGGKIFSSFSPRLPPTS
PYLDAVGGSPYGRPTAAAAAAGVYRYPPPPPAGNPASSTSPFGGAMRSSAGAAAAAAAGSAG
EATSMALAYNQLMSSMAAHFQQQQHLQQQHHHGLVAGSNASAAAAAAAAGKMSGSTSSATSP
GVLENLEKLAALAAGASSSSSSATNRNDMKSSVAGN
>irx2_Dappul
LLMSTGSPSGGGTASSSSSSSSSSSSAAALAALPGAASPGSGGDSVNATDGPATTATATTTT
NANNNSSNHSASAGSGSASPCCDSGRPLVTDPVTGQTVCSCQYERLQALQNGLAAGYGPAAA
AAAARLAAVSSAVTGVTGLHGVYSAAYPSSDQNPYPSLGMDSTTAFYSSLTNPYSLKEAGTG
GGDVSAWSTAPGLHQSAQGYYPYDPALAAYGYGASYDLAARRKNATRESTATLKAWLSEHKK
NPYPTKGEKIMLAIITKMTLTQVSTWFANARRRLKKENKMTWEPKNRNDDDDDDDCGESGRS
DDESSSPVHGNKQQHQSGTHNKKMKHSKIDKDDGDDKSDCDDDSRGSVSPHSIGSNSLNHLH
PHHHHHHHHLVHPSHHQHQHHHGGSGDVSVSVSVSNLMNNTYHHHAQQMAGYAAAHQHGMMD
MHQQHQQQLSSPTSSSAAKPKIWSLADTAACKTPPPPPGQHQIAGNSSGGSSGSSGSSMMAA
AMMHPAAAAAAAAAAAAHHHHHHLAAVGPANHHSWFSGVNGPMSSMATMTGAGSSAMMNGMS
NMAAMNAKMAAGFGSYGRYPSLTGYGNSAGYSSASAGSASAAYNGYTDAVQTDTPPQTPPNL
KLNANSLGQHQYHPYVSPQQQQQQQQQQQQRLSSMPLPPTMSGLSASAVDPSSEMPSPGSAS
ARSSQQQHSNLPMAAPSTTPSASSTSSAMMTSVAQPQQQHGSPGSGGESSSSTAFRPVYRTS
VEWTRGQQQSGAFPTDDDTLDTHQINSVPLMK

Calliphora vicina
>irx_Calvic
MAAYTQFGYGSYPSANQLLGGNVSQTSAAALLSSHNAPSPLSGTHENSLSPSGNSSASSSSS
VSSSGGGTTNGPLSPGAMSQTSTNHAGSVLTSNTTNATLAHALVNGPEGTTAAAATASSVLA
AAAVAAAAGGSSANHSPSGGGGVGSGSGGGCCENGRPIMTDPVSGQTVCSCQYDSARLALTN
YSRMPTGGVGVYGTAYPSTEQNPYPSIGVDSSAFYTPLSNPYSLKESTTGSDMSAWSTAGLQ
PTTGYYSYDPTFAYGYGASYDLASRRKNATRESTATLKAWLNEHKKNPYPTKGEKIMLAIIT
KMTLTQVSTWFANARRRLKKENKMTWEPKNRTEDDDDDAMLSDDEKELEKTDKAGAGGNMYG
QQMLKEHDQLKNNHHNVKHDLKDDEEDRKPENLTASRQLENYNTASLYGTTTGHHTNAYHPY
QSQQQSQSQSQSQSQQLQQHHSQSLYYQQQQQQQQQQGYLANPASTIRDECGIPIPATKPKI
WSVADTAANKTPPPNQAAYMSNSQQQQLAAQSYYANNLQINNHLANTSPLSMMSSYVNAAAS
SPYSRLNTAYPAASNYAATAGQMNNNSNTSANMMHLTQQYSAQKGHSQGFPEIQPDTPPNMK
HANSAM

Spodoptera frugiperda
>irx_Spofru
VDSSAFYSPLSNPYALKEGGGEMSAWTSAGLQPSGGYYPYDPTLAAYGYGAGYDLAARRKNA
TRESTATLKAWLNEHKKNPYPTKGEKIMLAIITKMTLTQVSTWFANARRRLKKENKMTWEPK
NKTDDDDDTMLSDEEKDKDEQEEKIKTHKDEERKGDEMLQGMHHQMLGYGIKEESKRGSSDC
GVPIPASKPKIWSLADTAACKTPXXAAQPWPQHAYGPPERAAPSDGGSQWVALPATA

Heliconius erato
>irx1_Helera
HEGPTYQDPITGQTVCSCQYDFLNYQRLASGVPLSMYSAPYPDAATAAGMAAYFPALAADQP
PFYANTAAGIELKENLAASAASWPYPTVYHPYDAAFAGYPFNGYGMDLNGARRKNATRETTS
TLKAWLNEHKKNPYPTKGEKIMLAIITKMTLTQVSTWFANARRRLKKENKMTWEPRNRVDDD
DNNNDDDDHKSNDGKDALDGKDSGTGSSEDGERPQQRLDLLGQRTESEWSESRADSG
>irx2_Helera
HEETACSCQYDGARLALTSYPRLSSAAVGVYGAPYPSTDQNPYPSIGVDSSAFYSPLSNPYA

LKEGSGEMSAWTSAGLQPSGGYYPYDPTLAAYGYGAGYDLAARRKNATTESTATLKAWLNEH
KKNPYPTKGEKIMLAIITKMTLTQVSTWFANARRRLKKENKMTWEPKNKTDDDDNTMLSDEE
KEQDDKIKPSKDDERKGDELLQGMHSQLLGYGIKEESKRGTSDCGVPIPASKPKIWSLADTA
ACK

Capitella Sp I

>irx1_Capsp1
MSFPTLGYSCSPPGSSSSLGSPQLLLPGALPSSAASSCEASAEPSSLSRSSADQEALTPSPA
PHLAPASLPPTMYSAASSLLSYGPAAARMLSDSVYTSAYAAQGYMPFGADPSAFYSPLVTGG
DNSEAWRSLSASTPGWPYDPSNMTGYPYGASYGGLDVRRKNATRESTSTLKAWLYEHIKNPY
PTKGEKIMLAIITKMTLTQVSTWFANARRRLKKENKMQWSPRNRAGDDDDDKNSDDDDDDDK
GDSNNNDDAKRLTEEDEDVVVDGVFTLYECIFDANDFSSSTQSLACPLAAIATVEQRDLWEL
DIRGKAQLSMRNRRGGRNCCNITSNDDSLLSTPPRADLMNGSSLLHPHQRCFDLTLGHKDVS
SSLLSRPTPTQPCAARLQGLAASSSSSSSSSAHLSSLTSPLANSEFGANPHKLQAEDKPRI
WSLADVATSSTPPSVKRPSPPPSLAGLHGYDNRSSSHLITEAREIISSAITQHIIHHRSTHR
YDTPADVTTQNFNNLRTPDRVGPDRVMTPELHDCAVKLSDFADDDSEATLTDGWAKYALKIK
RNCPALIVTTIGSPLIKATFPCVRKEVRKKYGSRDQVEKHSSSITQLTCLQRMIDTFTRELQ
ISGQHTITTFEQRNFTDRGLSKTAADTPRRQGDKPALPVFNEPRTWEISPRRKTRYHGCQWQ
RRRYNGG
>irx2_Capsp1
MKAGMSLSPSMCALPPYDIHGSYQYLSQAYASLDPRRKNATKESTNTLKAWLYEHKKNPYPT
KGEKIMLAILTRMTLTQVSTWFANARRRLKKERGGWTGEDDDDEEEEEVEVEKEEEADEEEA
RLRGDTPNPTAPSPRLSVVPITPPPSPPTTGQDRLSAPSKPHTIDHLPDRNQPAEPQKKPSI
WSIADMVQSSSSTSGQEKRREATPPNVTVSPTWSARDFSQHPYAHPYAMRFPHSVSSTPDHT
PLAPFRHPGLIIQPSNQLR
>irx3_Capsp1
MAFPQFGYPNIGASLPGNQNSLLLAAASAVNAPSSGASSRHTEAPNRDAPASSTPSSTAAQC
PCYPPGLIGSYPRMPGGLPEPNPAAAAAAATAALYGGAYPPGYLSLGAGEAVMNSMCGDLKS
TPELGLVPGSPYFPAYELAGFPYHNGFALDPNARRKNATRESTTTLKAWLNEHRKNPYPTKG
EKIMLAIITKMTLTQVSTWFANARRRLKKDNKVTWSPRNRGEDDDDDDDDENGRDKDEKDEE
FDVDIENDDDAEDSPLPRSSNKAEMSPLPSSPPPVAYSTHSAFTPINHLRSPPKQPLRVSTA
TPNKDSASSSPSSRSTKIWSLAEVASSKEHKTETNTPAFVTRGPTQQTFAPNQKAPHAAGFS
AFRPLLHGVPPQSLAGGPIQWPGSQAGGPHPAYSYALSQHYLAMTMARPGFIPASFHHPQDS
KLAGVR

Helobdella robusta

>irx1_Helrob
MKCGSRYGSLDVRRKNATRESTSTLKAWLYEHIKNPYPTKGEKIMLAIITKMTLTQVSTWFA
NARRRLKKENKMQWSPRNRTGEDDDDDDDDDKHSDNDDITTQNGEADMRNKSLDNDDDDDMM
MTRESDNTFDDDIRCADEEDDEDIVVDAGIDDLPVPDVVPVTNKTISNNNNCNQNNNSKVSS
SPSYHHNQSEYSRRSRSVVVSPMNVDNELQQIQQHHKQHFNILQHQQSLHHHLSQRSSQHDP
GNTSNTNNNRNNNDDSNNHKTNRENSPKFTQICHNFHPHFNRPSPFHMNNIHHHPSFHPSIT
FPQYPQPSFSHHHHSSPSFYPMFLTNKRKFGDKMEDEGHTLNKNPNNINNNDHNKNNNNNNS
NNVDHSNSNATNNKSKDGSYTDDAVRSKETEVDDDDDENSKVDIKMDSDPSSETTSITTTTT
TATKAAATTTSNFPPSISRPVKQDNITSECKKPKPKIWSIADVATSDDNKQRNNNKNTENVD
DGDKEAPDGDLNSPVQQPHQHLNAPYNMHLYSTLSQLYHPYFSQQHRQRMQVRFQQQLLPNN
YHQQFQQQQQ
>irx2_Helrob
MESSCLFSKSKFTSYLDNANISNTDINNTIYNDINTNNNINNYNNINNDNVNVNKFDPNILT
SYNLWQNYATTKLPYNWWAISQQQQQHQQQQQQTTRSYCDQQQQQHQRQQQQIPTTTKPTPT
TCFSSCKIPTYNPPPSSRLKKEKKVYWLPRNRATRESTSTLKNWLNEHIRNPYPSKGEKIML

```
AIITKMTLTQVSSAIKVSTWFANARRRLKKEKKVYWLPRNRADRSNEKIYNDEDNFACCHHC
SSSNDDDDESDDDDNDDNNNNSNINNNSSSNNILNTLTTTTKNASIKDFSLQISYINDGSHI
SNNINPHNNTITSNNINYSNNFNGNNNNISTDADDARNINSISDDDISANQPCYNNKIDSIN
FNPHTSNLTSDKKDSDDKCDDSDDDDDDDDADDKNSNNKKQRPDECSSSHSSSASTKYFLKN
KPDYNLSYNYFYANNFSAPNNINGNINYVDIKLLTADINSRIFSKLTPSSNDSSSKDNINTC
DISTSIYNSSTLSSCKYNGLTRNNVNNIKTRNTVNGNINEAHMNNSNNINNNYYNNNNRFII
DGTKNINNNITNTDDINSYDVNNLSYGANNNNNINYSINNTDYNNKNIFLQSVSTTFDNLKP
KIWSLAQVAENEQQEHFLGNEFATRELLSLPYHQLNYQQPNYLQQQPQQHQQQQQQPQILNN
HFYYNENRYSANNEHNSPIEIDNKNLNSTAEVENNINNIDNNISNNGNKINNIRDIFLNNNF
IQTLANNNTSIGQADINNNNNISKNNINEDDDDDDDDDDKIIDENANEDNDNDGEYDNDDINS
SWSPSFSSSSSSLSSATCSDKMDPKFF
>irx3_Helrob
MDNKSIINNNDGHNNNNNNNNNGNDIIDNDNNNNNNSCDVIVNNLQPYFYPIKPNFLPSLPH
NYNNHYIANVINNSNNNYNNNYNSNNINFNNYIQNNSITPSIINAYTPNNHIQNNNLPNNYS
QNNNYNYISDPTKRKTVDRESSATLKTWLASHGKNPYPSKHEKIALAIATKMSLTQISTWFA
NARRRLKKETNNNNNNNNNNNNSNNCSSSSSNSGRIIDNCENKSGDDDEDSNNNNNNSNDINY
NNNNNNNNNNNYTICYDDDVNETVTSTKEDHVITVASSSFSSSFSSPSSLPTLSPSSPAAAV
PSSSSSSSSSSSSSSLSPSISSSSSSKKGNNNNYDNNNNNNNNNNNKNNNNNISDSADCKNRE
IKIWSISQLLSND
>irx4_Helrob
LNQYIQKKNNRDENNLTQLNENELNYKKKTSLQAWLYEHIKNPYPTKGEKIMLAIITKMTTQ
VSTWFANARRRLKKENKMQWSPRNRTGDDDDDKSINSDSDDNNNHNSHNNNSTSTNNNSSSS
NICSNNTSSSSMSNIGNIKDYRINSSGSSSKINIGSSSSNNNNNNNNNNNNNSNNNNNNNNTNN
NSGKIYGDNDGKKHRDDDETIVVDNIVGDDILMSVSDRRKYTGTSLNRMAGSDMTSYLACNS
ISANKLNNSVMTNNNNNHNNNNNNNNNNNNNNNNNNNNSLYRFSYLSSSSSIFLNNNSNNNSQL
QHQQHNSSSTSSSSLQQQQQQNNPLTTTSTPQQQQQHFNPQLPSCANNNNNLTSPSSSLY
DSSPPLSAYNLFSSQPTPHNFYHSSPFISNPYINNPHNNNINNNNNNNSGFSQMFQSRDYNQ
YSLSRANFHHYNNNINNNSNSNKHSSTSNNNSTNAYVSDDDDNKNNSLNSGSGSCNYGNVSS
LLDGGLKPRIWSLADVATSAGGDQVEATSVSGYSIDENIYSSNNNNDDNNTDNSNRNNDVID
NNDINNNNNNNNNNNMNNGNNINNNNNSISMFNLSLNNQHHHYHFPTNHNYSHYFYSSPHVTS
IFPSSSSQSSSSSTSSSASSFVMKQQGK
>irx5_Helrob
RSYDVRRKNATRESTTTLKAWLYKHIKNPYPTKGEKIMLAIITKMTLTQVSTWFANARRRLK
KENKMQWSPRQRNDDDNDDDDDKNSLMSADDNDDDDEDDKVKKRSEAEAADDDNDDEDEEDD
EEEEEEEEINVDAGQLGEYILRCVANKLTNNKQIMLSHDCFIIKNTYKADSTNNKINKTSIE
NMFPDYLQQQRHHHQQQQHHHHQQQQQHQHNNIQTDDKLKPLTTTLSTEKPTMTSESENGS
SLPEAKKDKNKFRIWSLVDLATTTKSDSNDNDDVSDDDGAKTHKPKNINNNNDDDDDDDNNS
INNNKTSTDTNNSNTTTTNNENFNMSPPTSNMDIHPWMHRGIPASWVFQPKIAMAIYRQMMN
FHIQQQQHHHQQQQHNNNNNNNNKQQQPKH
>irx6_Helrob
CILQNLFYHYITNIIIIFIIFFVIIIIIIIIIPVIIIISYGVIDVRRKNATRESTTTLKSWLY
EHMKNPYPTKGEKIMLAIITKMTLTQVSTWFANARRRLKKVSREKINEMKMVWSPSKRTNNN
GDDDDNDDDDDDDDDDDDDADVSIGQNIDDDVNIDVHNNNNNNNNNINFNNNSSSSNNNTNYND
DDENDNNNNKNYDTSYAIAINDNNNNNNNNNNNNNNSNNNNNNNNNYRNFSNNNNEKADDEDDD
DDVYNVLKVKRRKRFLKEFSDTRYFKRGYIINNNNNHNNNNNSSNNIPDENSRNIDNNIQNI
KNNFNYINNNDDYHHEFNNRNNNDNSNNDLEINNNNNNNNNNNNNNNNNNNKRICDENDDDVEM
NCDDNNNIINKNNNNNNNKKSISSDKNNSSNNNYNNNNNNDDDSWTLCKDKSTKCSPDYDCIY
FLQHQQKHQHQKHQQQHQQQHQQHLQQQQQQKQQNVYHKSNSNNSLGYNKNEDANDDVGSDY
DDDDDDDDNNNNNNNDNNNNNKKIIISRSVKLKFDDDDDDDEEEDVTGEINGNYDNNDLNKN
NNNKNNNNNNNNNNNYDCSNYDDNNETNDINNNATEPTNYISDENNLNSGELFTKTTTTTLEI
SQSSSSSSSSSAAAAAVAAATATLLPSLSKPLPLTSSSTSSSSSSLSSNKKDTLPSTKSPNL
```

SSSSLSRSSSLLTSSASHSLFHNHQQHYHHYQHRHSIHHYHHNQMRLKPQNQHRFVDENNNN
NNNKRLRIWSIDDVIGNRF

Lottia gigantea
>irx1_Lotgig
XVPALGDSVYPTSAYASQGYMPLGADPSAFYPPLNGHYDIKDAGESWRGINQPAPCYPYDTP
MSSFPYSNAYGSVDLNSATRRKNATRENTTTLKAWLYEHKKNPYPTKGEKIMLAIITKMTLT
QVSTWFANARRRLKKENKMTWSPRNRSGDDNDDDDDDDGDKSKGDSDDDKEKDEVNLKEECK
SPVGTPSFSVTHNDHSSLFHEDTNKPINGGISCSGNLSLASSSLVGTAMGAAQSGIRTQNLG
ATPMDTKRPDSLCSNSNDSGLSDVNCNYSDVHNLENGMRPKIWSLAHVATSKAGFLPPPMGR
GLVSNDQNHDMKELSNNQLWGDMGVSNNRQNTYSSMSSCSLNNSGMMRQEPSAFTPNINPLS
SPNSNPVPPNINPYSQLYCPPRVPLD
>irx2_Lotgig
MSYNLTFPPNNTQIPAQQVTSPSTPTTPRACCENGRPVMTDPTTGQTICSCQYGSALLNYPR
LPGLTSSLFGSTAYGNTTGQSYVGLGTEGSAFYSPLGGNSTGQTQDAWRSLAQPAFSYDPSM
GLYPYGPGYGGLDLNARRKNATRESTTTLKAWLNEHIKNPYPTKGEKIMLAIITKMTLTQVS
TWFANARRRLKKENRMTWSPRNRGEDDDSDDEKENEDDEKSKLGDKKEKKINGDGSEIRDEE
IDVESENLDKNKKKVNDNLFLGDCVSPVPSVGEDSRSRLSPPVSRDSDSSFSESLNPSDCDD
QQKPKIWSVSDFLQTTPQKLGKPESLLLDSNGVPKIGSGMTSSFTPVSASAGLNGLFSSYPS
SLAYSSHSYPYPISTSGVGKLMSRSPATRFNPYNPAGRQPVANGFNAPRDFNVVREGE
>irx3_Lotgig
MTTAPSAPPSGRACCENGRPILTDPHTGQTICSCQYGAALLNYPRVPGLAESVYSASAAYAA
AASQGYVSLGAEGSAFYSPLAGGPYELKDGPEAWRALAQPGFPYDPTMIYPYGAGFGGLDLN
SRRKNATRENTTTLKAWLYEHRKNPYPTKGEKIMLAIITKMTLTQVSTWFANARRRLKKENK
MTWSPRNRSEDGSDGLDNIDVDSEGGKDDKDGRSTPGLGDLGSGRSTPAGVDDLDTLDGKDA
FINVDDEDSDDHGYHADRLDSDEEVLRRMKGKSDSEKLCDSASVTDNHSPNKTENRDSNVQS
GGNSTGTSSQQNTEQSPRPKIWSISEFLHKTSTTTGTTSSRPAAISTHARPPHTSGLFYIPG
APNGWNGARYHGVHPYSMSLSHSTISYPYSITSHTANKSPLPSTATLRTIDSLKEGEKLMRP
NVLFSPARDLDGIRPNX
>irx4_Lotgig
MFRAPFGLGGFPAGITADGVSVLTQNCGLSGIRADRPESWKALPSTMSPHPYDPSLAFHPYN
TFYAGLDLNSAARRKAATRETTSPLKAWLSEHRKNPYPTKAEKIMLAIITKMTLTQVSTWFA
NARRRLKKENKLCWDSADDKDDDDEDDIKNIDDESRRGFDSDREIDVEGLEKRLSGDEDSDN
QLDLSDISDTENEPTDNKNFKLQSRLLPSSQLRIPDNILVNKPVNTSNNNGVSKPKIWSISQ
IIDKDTPKPVPQQPVFTHYNGLHHTGSRFPSFVDPYGARLQMHIPGYNQEKLPSRPTLDTPP
DTPPFSISTKEIISPIKLNAATPEHEEALNLKTERPETQQTSX

Schmidtea mediterranea
>irx1_Schmed
MSSGKEGRPTWNSALLLEEFRLFDSTMNTVQISLSGGIPDQINPIFNYSSLQTQQNNDPSSL
LNLYNPAYAAAAQSRLLSSLHPSTSPNHSNINSTDTSTVSSITSAEQPPAPTFGPDSISPNT
NIPFYSSPQTFTNNNNHLCCDFGSAWRSAAHMMAADHNPYATYDNSAWANPYGYIGNPTLDLN
SARRKNATRESTSTLKAWLQEHIKNPYPTKGEKIMLAIITKMTLTQVSTWFANARRRLKKEN
KMTWSPKNRSDDATNSDQETDTKCKETDSDDGLKTDCQLDTLSDQDDLAMKSSFPSKLNDVK
PKIWSLAHTATAGLFSMPLQSYPGHCTDGSAEDPQNNSNINQYPANIGMPLISSHPLYQQGF
FHHIDLPNQLNGCQDNKQYSHLYHQQNPNNFFNHSQKRSEML
>irx2_Schmed
MSKTITSHTSTSCSGTIRKLSDDTAMSKLASIVEPIFHSPTMNRHLTQTSWPPGMHSNIHPS
NMFGGLMNRINSSPPLRQWINVSNQNHQVPSSAAFATQLPPARMINSESPLNASNNQMLMDH
WETWRQIHQATINNNLSPHFLALCQSSGITFNNNENDNVSNLIYKNIYVSFSIRYPPDFPNA
PRRKNATRETTSILKAWLNQHKKNPYPTKGEKIMLALITKMTLTQVSTWFANARRRLKKENK

MTWTARTRHEDDIDETGDDDEDDDDEEDNENDDDISKSDSRFESELRKNDESFLSKSEKSDT
SADETSKSNKIWSIAEIADDNYSGSNSRQHSNFSVLPTPMNLFRLPTGLNQYSDHLKLLQSM
TMTPFPSNIPVPIHHTYRNILKSWSVLNLTNLNSDASSWQPPLNLTNSKLSNKDPNKDYSTI
SETHKEIDRFSDQSSPVSLNKDS
>irx3_Schmed
MNSHPRRKNATRETTSILKAWLNEHKKNPYPTKGEKIMLAVITKMTLTQVSTWFANARRRLK
KENKMTWSPNQKSEDEADDLEDTDSSDIRSRLQDDPASLTKNYSKELKNEFLSKLSAINNLS
ADISNPKINSNVTSKSTKPKIWSIADITSDNVEFSQDKSSSNSLASSNEKDCQIYSSDPLKN
ITTQNPAVQFL
>irx4_Schmed
MCLPFRLLDCVQTQATGYKYNVLFVKQNGNLMNCLKNHEEELRDRNLFSTYYNCSRYGGIPP
EHIILANKVTGFLAMNDPNMDIRSQDVLENWRQFHQAALANNLNPITLAHLHTINHADVNQI
QVKKNATRENSSTLKAWLQEHKDNPYPSKGEKILLAILTQMSLTQVSTWFANARRRLKKENK
IGWIANGKSDNPDSTSDSELSELFTREYEKPNSNQKITKKNHIWSIIDMISEPSDCSHEDPA
T

Mytilus californianus
>irx_Mytcal
MAFAHQYTHAGTASMPGSQVMLSTKSHSPTAPSTMEQARALLHESGLPLLHFLPRMNGLPES
LYGQGPLTSNGLGVEPSAFYPMGSPPGHLTGSPEWKGIPGQSPVVYPLDPMLACHPYASFYG
GLDLNNAARRKNATRETTSALKAWLYEHRKNPYPTKGEKIMLAIITKMTLTQVSTWFANARR
RLKKENMTGWNGKDRDLDDSDFETDGDERTNENEGQYLLPIG

Mus musculus
>IRX1_Musmus
MSFPQLGYPQYLSAAGPGAYGGERPGVLAAAAAAAAAASSGRPGTAELGAGAGAAAVTSVLG
MYAAAGPYAGAPNYSAFLPYAADLSLFSQMGSQYELKDNPGVHPATFAAHTTPAYYPYGQFQ
YGDPGRPKNATRESTSTLKAWLNEHRKNPYPTKGEKIMLAIITKMTLTQVSTWFANARRRLK
KENKVTWGARTKDQEDGALFGSDTEGDPEKAEDDEEIDLESIDIDQIDERDGDQSNEDEEDK
AEAPRARVRPPASARDQSSPLSAAETLKSQDSPLGLVKEVSEPGSTRLLSPGAAAVGLQGAP
HSKPKIWSLAETATSPDGAPKASPPPPSSHASAHGPPSGSPLQHPAFLPSHGLYTCHIGKFS
NWTNGAFLAQGSLLNMRSFLGVSAPHAAPHGPHLTAPPPPQPPVQVATGVLHGEKASARSSP
ALPERDLVTRPDSPPQQLKSPFQPVRDNSLAPQEGTPRILAALPSA
>IRX2_Musmus
MSYPQGYLYQAPGSLALYSCPAYGASALAAPRSEELARSASGSAFSPYPGSAAFTAQAATGF
GSPLQYSADAAAAAAAGFPSYVGSPYDTHTTGMTGAISYHPYGSAAYPYQLNDPAYRKNATR
DATATLKAWLNEHRKNPYPTKGEKIMLAIITKMTLTQVSTWFANARRRLKKENKMTWAPRNK
SEDEDEDEGDASRSKEESSDKAQDGTETSAEDEGISLHVDSLTDHSCSAESDGEKLPCRAGD
ALCESGSECKDKFEDLEDEEDEEDECERDLAPPKPVTSSPLTGVEAPLLSPAPEAAPRGGSG
GKTPLGSRTSPGAPPPASKPKLWSLAEIATSDLKQPSLGPGCGPPGLPAAAAPASTGAPPGG
SPYSASPLLGRHLYYTSPFYGNYTNYGNLNAALQGQGLLRYNTAASSPGETLHAMPKAASDT
GKAGSHSLESHYRPPGGGYEPKKDTSEGCAVVGAGVQTYL
>IRX3_Musmus
MSFPQLGYQYIRPLYPPERPGAAGGGGGGSSAGGRSGPGAGASELAASGSLSNVLSSVYGAP
YAAAAAAAAAAQGYGAFLPYATELPIFPQLGAQYELKDSPGVQHPATAAAFPHPHPAFYPYG
QYQFGDPSRPKNATRESTSTLKAWLNEHRKNPYPTKGEKIMLAIITKMTLTQVSTWFANARR
RLKKENKMTWAPRSRTDEEGNAYGSEREEEDEEEDEEESKRELEMEEEELAGRGGGHGGRGA
GRRRRDEEIDLENLDSAAAGSELTLAGAAHRNGDFGLGPISDCKTSDSDDSSEGLEDRPLSV
LSLAPPPPPVARAPASPPSPPSSLDPCAPAPAPSSALQKPKIWSLAETATSPDNPRRSPPGA
GGSPPGAAVAPPTLQLSPAAAAAAAAAHRLVSAPLGKFPAWTNRPFPGPPAGPRPHPLSMLG
SAPQHLLGLPGAAGHPAAAAAAYARPAEPESGTDRCSALEVEKKLLKTAFQPVRRPQNRLD

AALVLSALSSS
>IRX4_Musmus
MSYPQFGYPYSSAPQFLMTTNSLSTCCESGGRTLADSGPAASAQAPVYCPVYESRLLATARH
ELNSAAALGVYGSPYGSSQGYGNYVTYGSEASAFYSLNSFESKDGTGSSHAGLPPTAAAAYY
PYEPALSQYPYDRYGTVDSGTRRKNATRETTSTLKAWLQEHRKNPYPTKGEKIMLAIITKMT
LTQVSTWFANARRRLKKENKMTWPPRNKCADEKRPYGEGEEEEAGEEESREEPLKSAKSEGH
AGKDDKELELSDLEDFDPLDAETSECELKTPFQSLDSGPERIPASSDGPGTGKEASTTLRMP
LGTAGGAVMDGDLERARNCLRSTVVVPDSGAEGGPPACEAKLTFAQAGAPPNLETKPRIWSL
AHTATAAAATALSQTEFPSCMLKRQGPTGVSATTPASSPAVTAPSGALDRHQDSPVTSLRNW
VDGVFHDPILRHSTLNQAWATAKGALLDPGPLGRNLGAGTNVLTTPLACSFPPTVPQDVPPA
GASRELLATPKAGGKPFCT
>IRX5_Musmus
MSYPQGYLYQPSASLALYSCPAYSTSVISGPRTDELGRSSSGSAFSPYAGSTAFTAPSPGYN
SHLQYGADPAAAAAAAFSYVGSPYDHTPGMAGSLGYHPYAAPLGSYPYGDPAYRKNATRDAT
ATLKAWLNEHRKNPYPTKGEKIMLAIITKMTLTQVSTWFANARRRLKKENKMTWTPRNRSED
EEEEENIDLEKNDEDEPQKPEDKGDLEGPESGGAEQKATAGCERLQGPLSPAGKETEGSLSD
SDFKESSSEGRHDELPRPPRAGESSPAGPATARLAEDAGPHYPASVPAPGPHPSAGELPPGS
GGSSVIHSPPPPPPPPPAVLAKPKLWSLAEIATSSDKVKDGGGGSEGSPCPPCPGPMGGQTL
GGSRASPAPAPARSPSAQCPFPGGTVLSRPLYYTAPFYPGYTNYGSFGHLHGHPGPGPSPTA
GPGSHFNGLNQTVLNRADVLAKDPKMLRSQSQLDLCKDSPYELKKGMSDI
>IRX6_Musmus
MAFSPFGHPYGSTSQFLVSASSSATCCETAPRPVSDVASASTSASTLCCTPYDSRLLGSARP
ELGAALGIYGAPYAAAQSYPGYLTYGPEPPTLCGALNPQYEFKDAAGSFAPSLTQPGAYYPY
ETTLGQYQYDRYGGVELSSAGRRKNATRESTSALKAWLHEHRKNPYPTKGEKIMLAIITKMT
LTQVSTWFANARRRLKKENKMTWAPKNKGGEERKADSGEDSLGCLNGDTKDATASQEARGLR
LSDLEDLEEEEEEEEEAEEEAAVSAARRLADFQKSTQPLPAPCAAAQERCLESRECGLGLPRF
SFTEAPQSGEADFITAEPGGPTMILHYPSGHKPRIWSLAHTAAASAVESAPSTPPRAQSPEC
HMIPRQPSSIRRLLVPRDSEGEEDSPAAKAFGNSTFTLQGLPLNCAPYPRRREPEVRFQYPS
GAEAG

Homo sapiens
>IRX5_Homsap
MSYPQGYLYQPSASLALYSCPAYSTSVISGPRTDELGRSSSGSAFSPYAGSTAFTAPSPGYN
SHLQYGADPAAAAAAAFSSYVGSPYDHTPGMAGSLGYHPYAAPLGSYPYGDPAYRKNATRDA
TATLKAWLNEHRKNPYPTKGEKIMLAIITKMTLTQVSTWFANARRRLKKENKMTWTPRNRSE
DEEEEENIDLEKNDEDEPQKPEDKGDPEGPEAGGAEQKAASGCERLQGPPTPAGKETEGSLS
DSDFKEPPSEGRLDALQGPPRTGGPSPAGPAAARLAEDPAPHYPAGAPAPGPHPAAGEVPPG
PGGPSVIHSPPPPPPPAVLAKPKLWSLAEIATSSDKVKDGGGNEGSPCPPCPGPIAGQALG
GSRASPAPAPSRSPSAQCPFPGGTVLSRPLYYTAPFYPGYTNYGSFGHLHGHPGPGPGPTTG
PGSHFNGLNQTVLNRADALAKDPKMLRSQSQLDLCKDSPYELKKGMSDI
>IRX6_Homsap
MSFPHFGHPYRGASQFLASASSSTTCCESTQRSVSDVASGSTPAPALCCAPYDSRLLGSARP
ELGAALGIYGAPYAAAAAAQSYPGYLPYSPEPPSLYGALNPQYEFKEAAGSFTSSLAQPGAY
YPYERTLGQYQYERYGAVELSGAGRRKNATRETTSTLKAWLNEHRKNPYPTKGEKIMLAIIT
KMTLTQVSTWFANARRRLKKENKMTWAPKNKGGEERKAEGGEEDSLGCLTADTKEVTASQEA
RGLRLSDLEDLEEEEEEEEEAEDEEVVATAGDRLTEFRKGAQSLPGPCAAAREGRLERRECG
LAAPRFSFNDPSGSEEADFLSAETGSPRLTMHYPCLEKPRIWSLAHTATASAVEGAPPARPR
PRSPECRMIPGQPPASARRLSVPRDSACDESSCIPKAFGNPKFALQGLPLNCAPCPRRSEPV
VQCQYPSGAEAG
>IRX3_Homsap
MSFPQLGYQYIRPLYPSERPGAAGGSGGSAGARGGLGAGASELNASGSLSNVLSSVYGAPYA

AAAAAAAAQGYGAFLPYAAELPIFPQLGAQYELKDSPGVQHPAAAAAFPHPHPAFYPYGQYQ
FGDPSRPKNATRESTSTLKAWLNEHRKNPYPTKGEKIMLAIITKMTLTQVSTWFANARRRLK
KENKMTWAPRSRTDEEGNAYGSEREEEDEEEDEEDGKRELELEEEELGGEEEDTGGEGLADD
DEDEEIDLENLDGAATEPELSLAGAARRDGDLGLGPISDSKNSDSEDSSEGLEDRPLPVLSL
APAPPPVAVASPSLPSPPVSLDPCAPAPAPASALQKPKIWSLAETATSPDNPRRSPPGAGGS
PPGAAVAPSALQLSPAAAAAAAHRLVSAPLGKFPAWTNRPFPGPPPGPRLHPLSLLGSAPPH
LLGLPGAAGHPAAAAAFARPAEPEGGTDRCSALEVEKKLLKTAFQPVPRRPQNHLDAALVLS
ALSSS
>IRX4_Homsap
MSYPQFGYPYSSAPQFLMATNSLSTCCESGGRTLADSGPAASAQAPVYCPVYESRLLATARH
ELNSAAALGVYGGPYGGSQGYGNYVTYGSEASAFYSLNSFDSKDGSGSAHGGLAPAAAAYYP
YEPALGQYPYDRYGTMDSGTRRKNATRETTSTLKAWLQEHRKNPYPTKGEKIMLAIITKMTL
TQVSTWFANARRRLKKENKMTWPPRNKCADEKRPYAEGEEEEGGEEEAREEPLKSSKNAEPV
GKEEKELELSDLDDFDPLEAEPPACELKPPFHSLDGGLERVPAAPDGPVKEASGALRMSLAA
GGGAALDEDLERARSCLRSAAAGPEPLPGAEGGPQVCEAKLGFVPAGASAGLEAKPRIWSLA
HTATAAAAAATSLSQTEFPSCMLKRQGPAAPAAVSSAPATSPSVALPHSGALDRHQDSPVTS
LRNWVDGVFHDPILRHSTLNQAWATAKGALLDPGPLGRSLGAGANVLTAPLARAFPPAVPQD
APAAGAARELLALPKAGGKPFCA
>IRX2_Homsap
MSYPQGYLYQAPGSLALYSCPAYGASALAAPRSEELARSASGSAFSPYPGSAAFTAQAATGF
GSPLQYSADAAAAAAGFPSYMGAPYDAHTTGMTGAISYHPYGSAAYPYQLNDPAYRKNATRD
ATATLKAWLNEHRKNPYPTKGEKIMLAIITKMTLTQVSTWFANARRRLKKENKMTWAPRNKS
EDEDEDEGDATRSKDESPDKAQEGTETSAEDEGISLHVDSLTDHSCSAESDGEKLPCRAGDP
LCESGSECKDKYDDLEDDEDDDEEGERGLAPPKPVTSSPLTGLEAPLLSPPPEAAPRGGRKT
PQGSRTSPGAPPPASKPKLWSLAEIATSDLKQPSLGPGCGPPGLPAAAAPASTGAPPGGSPY
PASPLLGRPLYYTSPFYGNYTNYGNLNAALQGQGLLRYNSAAAAPGEALHTAPKAASDAGKA
GAHPLESHYRSPGGGYEPKKDASEGCTVVGGGVQPYL
>IRX1_Homsap
MSFPQLGYPQYLSAAGPGAYGGERPGVLAAAAAAAAAASSGRPGAAELGGGAGAAAVTSVLG
MYAAAGPYAGAPNYSAFLPYAADLSLFSQMGSQYELKDNPGVHPATFAAHTAPAYYPYGQFQ
YGDPGRPKNATRESTSTLKAWLNEHRKNPYPTKGEKIMLAIITKMTLTQVSTWFANARRRLK
KENKVTWGARSKDQEDGALFGSDTEGDPEKAEDDEEIDLESIDIDKIDEHDGDQSNEDDEDK
AEAPHAPAAPSALARDQGSPLAAADVLKPQDSPLGLAKEAPEPGSTRLLSPGAAAGGLQGAP
HGKPKIWSLAETATSPDGAPKASPPPPAGHPGAHGPSAGAPLQHPAFLPSHGLYTCHIGKFS
NWTNSAFLAQGSLLNMRSFLGVGAPHAAPHGPHLPAPPPPQPPVAIAPGALNGDKASVRSSP
TLPERDLVPRPDSPAQQLKSPFQPVRDNSLAPQEGTPRILAALPSA

Gallus gallus
>IRX1_Galgal
MSFPQLGYPQYLSASQAVYGSERPGVLAAAAAAAAAAAAASGRPAGAELGSGSAAVTSVLGM
YAGPYSAPNYSAFLPYTADLGLFSQMGSQYELKDNPGVHPATFAAHTAPGYYPYGQFQYGDP
GRPKNATRESTSTLKAWLNEHRKNPYPTKGEKIMLAIITKMTLTQVSTWFANARRRLKKENK
VTWGSRSKDQEDANLFGSDNEGDPEKTEDDEEIDLESIDIDKIDENDGEQSNEEEEEKPELL
RQSSEEERLEKDKELSLSGSEGLKPKDALAMVKEASDNSTRIISPGGQSNLQMPSHSKPKIW
SLAETATSPDGALKSSPPPPPPAQVTHTLPQIQHPAFLPSHGLYTCQIGKFHNWTNGAFLTQ
SSLLNVRSFLGVNHHHATHHNHHLQAQQQPSVLTATLGALSSEKPSERTSPKHIENVPRTDS
PPQPLKSPFQPVRDNSLPQQEGTPRILTALPSA
>IRX2_Galgal
MSYPQGYLYQPPGSLALYSCPAYGASALAAPRSEELARSSSGSAFSPYPGSAAFTAQAAATG
FTSPLQYSTDPATGFPSYMGSPYDAHTTGMTGAISYHPYGSPAYPYQLNDPAYRKNATRDAT
ATLKAWLQEHRKNPYPTKGEKIMLAIITKMTLTQVSTWFANARRRLKKENKMTWAPRNKSED

EDDDDGDGARSKEESPDKIPESNETSAEDEGISLQVDSLTDHSCSAESDGEKLPCRAGDPLC
ESGSECKDKYEDIEEEEEDEDDDEEDIEEDDGGGGERDPPAKPATSSPLAAVEAPLLGHPHA
DAARSASKAALGGRASPGPPTPASKPKLWSLAEIATSDLKSQNLGQGCPPAALASATPASAP
HSAAYSPSSLLGRHIYYTSPFYSNYTNYGNFNALQSQGILRYNSAAVASNEGLSQTVLNASS
VHKQSSDSLKTITNQLEQHYRPSSYDSKKDPTEVCTVGVQPYL
>IRX3_Galgal (incomplète)
MSFPQLGYHYIRPLYPFYCPGSGGSRGGAELAPSGTLSNDADLLHSDSKTALEGSEGFEDLP
GSHERYLGPAAAAAAEPLRLRHHHLRHHRHHRRPPCELPSAGPEPPPPAPPPPPPPPPHLSP
PSSASSSSASSPTDAASAGTVPKPKIWSLAETATSPDNPRKSPGSPPAAAPQPLPLPPPPPH
RLVSSCPLGKFPNWTNRAFPAASPHHHAAPHPLALLNTPHLLGLGAAPAAAAAFPRPAEQAP
SAEPPGADRSIALEVKKKLINTAFQPVQKRPQNQLDAAMVLSALSSS
>IRX4_Galgal
MSYPQFGYPYSSAPQFLMSTNSLTPCCESGGRTLPESGPAAPAQTPVYCPVYESRLLATARH
ELNSAAALGVYGGPYGGPQGYGNYVTYGTEAPAFYSLNTLEAKDGGGSAHAGISPAAAYYPY
EHSLSQYQYDRYGAMDGGTRRKNATRETTSTLKAWLQEHRKNPYPTKGEKIMLAIITKMTLT
QVSTWFANARRRLKKENKMTWPPRNKCSDEKRPYEEEEEEEEECSQEDAMKSEKAEEPTGKE
EKELELSDLEDLDAAESESSECEMRRPFPHPHPHPLPGGGPPPRAAEPPAAAAEEEEEEAAE
RARGCLKPAAEECEAALLGARPRGCEAKLCFPQGQPLLEAKPRIWSLAHTATSLNQAEYPSC
MLKRSGGSAAAAVSAPVSVMDRHQDSPVTNLRNWVDGVFHDPLFRHSTLNQALSNTTVSWAT
TKGAILETGALGRSVGNGANVLKGQLANLAHQDSSKEFLAFPKAGSKMFCS
>IRX6_Galgal
MSFSQFGYPYSTTSQVRLKQIARSSHSERRLENRCFLPPLPFHRSLGRATNTHARFVPSCRA
PRAPLPAEQPSDPAPRGAPHRRPAPRPGLGGTVRAPQARRADIFTALSIVELTIIATSCLEI
DANGSRGAGRGKPRGSFAILESDRCLRVPPAPLRASPLPAPGAPPPPPAPAAPGLPRGRSAR
PVSPPQFLVPGSPGASCCEPAPRSGPDASSAPAAASLCCAPYESRLLAPGRAELNAALGMYG
APYAAGQGYGNYLPYSAEPAAIYTALNPQXGIKEGAGTLHSGTRRKNATRETTSTLKTWLYE
HRKNPYPTKGEKIMLAIITKMTLTQVSTWFANARRRLKKENKMTWSPKNKAGEERKEDGTRH
DGEYGAGSDGRGKSILNIEYTSLCLPKVPKAAEGTPGGADVGPGAAEGQRGRAQAGWERGAL
RSRGGPERRGSSALCRSDPQLRVLARRLARSSCATGGDGCRPEERSTTRLHPDSCGGALRGR
NARSRPREAKPRSPPGSAPSGGGGSAGPGSFGMVLRQARRSSERDADYPVRPPRTPPQSVTG
KIPRIDGEQIPGAEHGEGERDPRAPEERTVPVPLRPATTARSEERMLPASPGRSQCRRPGPA
VAAHTPLRPLKGRAALANELFSETIKGSAGRGPMKGRLCAVETARLGPAPPGFLRPRGDPPC
GTARRGQRHSPGGSSGLQPRRWGPLSRSSCPRAAAVSKSDGLLRGGRRGGESCRKVLWGDVK
EGSSTATGWEGGDS
>IRX5_Galgal
MSYPQGYLYQPSASLALYSCPAYSTSVISGPRTDELGRSSSGSAFSPYAGSTAFTAPSPGYN
SHLQYGTDPAAAAAAAFTSYVGSPYEHPPAMAGSLGYHPYAAPLGSYPYGDPAYRKNATRDA
TATLKAWLNEHRKNPYPSKAPAAASVIHSPQQAALAKPKLWSLAEIATSADRAREAGGETAA
PGPAVLGGGGPPRPSPPRPRSPSAQCPFPNGAVLPRPLYYTAPFYPGYTNYGSFGALHGHPA
GGPAAPGAAFNGLNQTVLSRAEGLGKEPKAARSQPQAELGKDSPYELKKGMSSI

Xenopus tropicalis

>IRX2_Xentro
MSYPQGYLYQPPGSLALYSCPAYGASALAAPRSEELARSSSGSAFSPYPGSAAFTAQAATGF
SSPLQYSSDPAGFPSYMGSPYDAHTTGMTGALSYHPYGSAAYPYQLNDPAYRKNATRDATAT
LKAWLQEHRKNPYPTKGEKIMLAIITKMTLTQVSTWFANARRRLKKENKMTWAPRNKSEDED
DDEGDGERVKEEQSEKAQDCNETSAEDEGISLHVDSLTDHSCSADSDGEKLPCRATDHLCES
GSESKEKYDDDEDEEEGDEEDRVLPVKPATSSPLTGVEAPILNHQQDGSPRNSNKTSLDNGM
SPSSQTPASKPKLWSLAEIATSDHKHSNLGSVLSSATSSAAHNPSYPSSSLLGRHIYYTSPF
YSNYTNYGNFNALQSQGILRYSSAAVTANEGLNQTVLSTSSMHKHTSDSVRTASNQLDQHYR
PTNFESKKDPSEVCTVGVQPYP

>IRX1_Xentro
MSFPQLGYPQYLSAGQAAVYGGERPGVLAAAAAAAAAAAAAGSGRPTGADLGSSSTAAVTSV
LGMYASPYSAPNYSAFLPYTTDLTLFSQMGSQYELKDNPGVHPATFAAHTTPGYYPYGQFQY
GDPGRPKNATRESTSTLKAWLNEHRKNPYPTKGEKIMLAIITKMTLTQVSTWFANARRRLKK
ENKVTWGARSKEDDNIFGSDTEGDHEKNEDDEEIDLESIDIDKIDDNDGEQSNEEEDEKLEH
LRQGEKESFKKESEVMIPSSDGLKSKDSLSLGKESSDTSNTRIVSPGGQGNIQVPPHNKPKI
WSLAETATSPDGALKSSPPPSQANHTSPPIQHPAFLPSHGLYTCQIGKFHNWTNGAFLTQSS
LINMRSLLGVNPHHVAHHNHHHLQAHQQAPLLATNLSSLSSDKTPERTSPKHSDRENLPRTE
SPPQLKPSFQAVRENTLSQQEGTSRILTALPSA
>IRX3_Xentro
MSFPQLGYQYIRPLYPSDRQNVGGTRSGTELSPAGTLSNVLSSVYGTPYAAAAAAQAYGAFL
PYSAELPIFPQLGSQYEMKDSPGVQHAAFSHPHAAFYPYGQYQFGDPSRPKNATRESTSTLK
AWLNEHRKNPYPTKGEKIMLAIITKMTLTQVSTWFANARRRLKKENKMTWAPRSRTDEEGNA
YGSDHEEDKHEDDEEIDLENIDTEDIESKEDLDDPDTDIHSDSKTDARSDSEASDGFEDLNA
PEDRLLKSVVGQRQVLNEEPQDKCALSSDAKASQPACEQIKLDRIPSSPPLENNIPAAHKPK
IWSLAETATTPDNPRRSPNTGGSVNTQNLIAQHRLIASPGSRFQGWTGRAFSAQQLSLLNSA
HFLQGLSVSHTALGSGTASFPKAAEPKHSTDSLTDRSSTVDIEKKIPVLNTAFQPVQRRSQN
QLDAAMILSALSSS
>IRX5_Xentro
MSYPQGYLYQPSASLALYSCPAYSTTVISGPRTDELGRSPSGSAFSPYAGSTAFTASSAGFN
SPLQYSGDPAAAFTSYVGSPYDHSAGMAGSLGYHPYAAPLGTYPYGDPAYRKNATRDATATL
KAWLNEHRKNPYPTKGEKIMLAIITKMTLTQVSTWFANARRRLKKENKMTWTPRNRSEDEED
DENIDLEKNEEDDPRKLEEKGDQDGDAGDQKRSPSAVDFDRLEGEVRQGKELDQTRSDSEQN
EVEERNDLLSKNSAPPTSPLCPPDQSPQAQEDQNLHRHTVHNHHHQSIQQLHHHSHQSHPLD
LVHRNALVQHGPVTNNATSVIHSPPASTSKPKLWSLAEIATSSDKVKESSDAAAVAGTTPAQ
SMVVSASSPSRSPSAQCHFPSNTVLSRPLYYSNPFYPGYTNYGTFSHLHSHHGPSTTSPTVN
STHHFSGINQTVLNRAEALAKECKLRSQSQADLNKDTPYEMKKGMSSI
>IRX4_Xentro
MSYPQFGYPYSSTPQFLMTTNSLSTCCESSGRSLSDSAAAASAQTPVYCPVYESRLLATARH
ELNSAAALGVYGSPYTSTQGYGNYVTYGADASAFYSLNAFESKDGTGSAHAGIPQTAAYYPY
EHTLSQYQYDRYGTMDGSTRRKNATRETTSTLKAWLQEHRKNPYPTKGEKIMLAIITKMTLT
QVSTWFANARRRLKKENKMTWPPRNKCSDEKRPYDEEEEEEEEEDSQKATIKNEKKIVDEEV
AREDKALDLSDLEDFDAIESESSECELKQPFHHQPQDGHQLRQRDCVNDHCKDVILKMPITP
AANQELDRTKICHKPGVDQCEQEVLRGRQRGGESKACFQQQQILDSKPRIWSLAHTATSLNQ
TEYPSCMLKHQGLSSPSSSSSSSAVSTPVCVIDRRQDSPVTSLRNWVDGVFHDPLFRHSTLN
QALTNTTVSWATTKGTLIDSGSLGRSVGNPTNAIKGQLPNIPHDTNKEFIAFQKSGSKMFCS
>IRX6_Xentro
MSFPQFGYPYSSNSQFLVSTNPSTTCYDSAPRSLPDVPVVSTQTPSIYCPPYENRLLVSSRT
DLSLGVYNTTYATASTAYASYIPYNADPTTLYPTLTPQYEMKDSTSSLHPGIAQPAAYYPYE
HSLGQYQYDRFGAVDISCSSRRKNATRENTSTLKTWLYEHRKNPYPTKGEKIMLAIITKMTL
TQVSTWFANARRRLKKENKMTWSPKNKAMDEKKDEKQEDYNSGCEDQENSDHLVTYGTTGKP
RIWSLAHTAASSIAVEIENDKKSLSPKCVFVKKIHSGSGYSICKEMKALQHIKPQIQTDNDC
EVPPPTTRMFRSSS

Brachydanio rerio
>IRX1a_BRARER
MSFPQLGYPQYLSASQAVYGGDRPGVLTPSSRAGSAEIGGSASAAAAAMSSVLGMYPYAHNY
SAFLPYTSADLALFSQMGSQYDLKDSPGVHPASFAAHTSPAFYPYGQFQYGDPARPKNATRE
STSTLKAWLNEHRKNPYPTKGEKIMLAIITKMTLTQVSTWFANARRRLKKENKVTWGARSKD
DENIFGSDNEGDAEKNEDEEEIDLESIDIDKIDDNDGDQSNEEDEDKRAELESLQEKRHASK
EPSDGNNNTTRVLSPGRQGGFQVPVSNKPKIWSLAETATSPDSAQKPTSPSVPVSHPAFLPS

HGLYTCQIGKFHNWTNGAFLGQNSLLNVRSFLGVNQHNHPAQQQQQQTGPASLQVLSAGNTD
KMPEDLSPKHIDRENVLRNESPTQPLKSSFRPLHDSPRNQQESTQRVLTALSSA
>IRX1b_BRARER
MSFPQLGYPQFLNASQEVYGAERTGVMPSSPREGSSDNSANPATAAAAITPMLGMYANPWTV
QNYSAFLPYNSAELALLSQMGSQYELKDNPGAHPPGFAVHAAPGFYPYGQYQYGDPARAKSA
TRETTSTLKAWLQEHKKNPYPTKGEKIMLAIITKMTLTQVSTWFANARRRLKKENKVTWGRS
AEDRDGRIFDSDNEDDADKNDDEEEIDLETIDMDKAEEPRVDQNKEAANNPHAIENIETPRI
LSSPALKNTDSPMSSGNREQNVVKSVGEVSPGGAVCQRPGNSKPKIWSLAETATSPDNTQKL
SIPSTPAGLRASPSTHPAFLSTNGIYTCQIGKLHNWASAAFLSANSLMGVRSLLSQNSNFPN
HSPVTNPEKKPSSASGSPCLDSDNSSDSFSPKHNDQENVQRIESPTLASRSPFPVIHDRSHH
ETSQRALKTIS
>IRX2a_BRARER
MSYPQGYLYQPPGSLALYSCPLAAPRSEDLARSSSGSAFSPYPGSAAFTASANGFSSPLPYS
TDPATGFPSYMGSPYDAHTTGMAGAISYHPYGSPGYPYQLNDPAYRKNATRDATATLKAWLQ
EHRKNPYPTKGEKIMLAIITKMTLTQVSTWFANARRRLKKENKMTWAPRNKSEDEDEDDGDG
ERKDERTDKNMDNSEASAEDEGISLHVDALTDHSCSVESDGEKVTCRTGDLVCDSGAEIQDK
CEATDLGEERQRGASPKPVTSSPLTGVEAPLLTHHHRENSTNKTCLDGQNQTVKPKLWSLAE
IATSDPKQQNCPPGVGLLTSSAPGASPAGAVYPATSILGRPLYYTSPFYSNYTNYGNFSPLQ
GQGILRYNSAAEGLLHKQTGEPLIKTNPNQTEAHFRASNAESKKDPSEVFTVRSQSYLSS
>IRX3a_BRARER
MSFPQLGYQYIRPIYSQDRQGIGSARAGTDLSPSGALSNVLSTMYGSPFAAAQSYGAFLPYS
NDLSIFNQLGAQYELKDSPGVQHPGFAHHHPAFYPYGQYQFGDPSRPKNATRESTSTLKAWL
SEHRKNPYPTKGEKIMLAIITKMTLTQVSTWFANARRRLKKENKMTWTPRSRTDEEGNVYNS
DHEGDDGDKREDEEEIDLENIDTENIENKDDLDEQDELHSDLKLDGRSDSEISDGYEDLQGP
EQRLFKAMVKDGKEIHGDRAEHFHHHSHHHHLHHNSLEQANGEPVKINQAIINSPPSENNPP
PAPKPKTWSLAETATTPDNPRKSPLMNGTSAASATQTIITPHRLLSCPVGKIQSWTNRGFTA
HQLALLNSNHYLGLSNQASANGLALYSRQAEDRSQNSESTVTERSSALEAEKKLLKTAFHPV
QRRPQNQLEAAMVLSALSSS
>IRX3b_BRARER
MSLPQLGYKYIRPLYSTERRGGAEISVTGSGSLSSALSGVYGAPFASTAQGYSAFLPYSSDL
SVLNQLSSQYEFKDSPGLQHAGFPHAAFYPYGHQYQFGDPSRPKNATRESTSTLKAWLSEHR
KNPYPTKGEKIMLAIITKMTLTQVSTWFANARRRLKKENKMTWVPKTRTDEDGNVYTSDNED
AEKRDEDEEIDLENIDTEDIEDKQDCDYQDDDKSTPKGSDSEEYDDARAEKRIIEDEEQIKK
SPAEEQEPSNNISPALKPKIWSLAETATTPDSPKKTWIQRNCDAQTVRNPLHVQNWTKMALS
AHQMAFTSHYLGLKHQSITNIHVKHAEQRTHSL
>IRX4b_BRARER
MSFTQFGYSYPAAPQLLMSSNAPSTCFESSGSLLDSGVPTSPQNSLYCPVYESRLVASSRHE
LIPTAVYGNSCSKNHVYDSCSTYGPDSAACYPLGKLNEKDGVQTGHSRVTQSSAYYPYDYPI
GQYSYDRYGYGSVDVGTRRKNATRETTSTLKAWLQEHKKNPYPTKGEKIMLAIITKMTLTQV
STWFANARRRLKKENKMTWSPRNKNSDEKECDDDQEDLDEAQEEPIKTEQDFDDNHGKDDTD
QLHSDLDDFDLVESDGSECESKPSFVVHVHSETSDHPETHFKDAFHESVTELTRGVHKFSED
RLRAPAEDHQMAKFYLQQGQKTIETKPKIWSLAQTATSLNQVDYSSCMHKRTACPSSNCDSD
SEMTKRKQESPVATLRNWVDGVFHDPLFRHNNLNQTFSNSTELWTDAISSQNNYHEHSGTPI
TSSHA
>IRX4a_BRARER
MSYPQFGYPYSSAPQFLMTTNSLTSCCESSTRSIPDSSAQTPVYCPVYESRLLATARHELSS
AAALGVYGSPYTGGQGYGNYVTYGADASAFYSLGTFDAKDGSASAHAGITPAAAYYPYDPTL
GQYQYDRYGSMEGGTRRKNATRETTSTLKAWLQEHRKNPYPTKGEKIMLAIITKMTLTQVST
WFANARRRLKKENKMTWPPRNKGSDEKKYDDDEDGSPEEQIKSETNDDESKDREDKDLQLSD
LEDFDPLESESPECELKHRFQISAHMETKDPQKLSIPGLLEEAKSCLKSPEDCELELRHSKA
CFQQQQQQSHPILDSKPRIWSLAQTATSLNPAEFPSCMLRCPASPSPAGGPDRQQDSPVTTL

RNWVDGVFHDPLFRHSSLNQTNTTVSWTNKGSILETGALGRSVADSGKQEAGKDTMTFPKGV
NKIFCS
>IRX5a_BRARER
MAYPQGYLYQPSASLALYSCPAYSASVISGPRTEELGRSSSGSAFAPYAGSTAFTSASPGYN
SHLPYSADAAAAATFTSYVSSPYDHTTGMAGSIGYHPYAAPLGSYPYGDPAYRKNATRDATA
TLKAWLNEHRKNPYPTKGEKIMLAIITKMTLTQVSTWFANARRRLKKENKMTWTPRNRSEDE
EEDENIDLEKNDDDEPNKPADKGDSTDTEADHKLINPGEIPCDRFKDETHSKDLDPPLTDSE
LKEAEERTDLLAEQAKPTTSSPSVLQRGSDLITQEKPSEPGHAASTGNSNVTSVIHSPPSAP
KPKLWSLAEIATSSDRCKGSSRLHRPAGLSQSTVIASVASPTRSSPQCPLPNNTVLSRPIYY
TSPFYPGYTNYSTFGHLHSSHGTNTSSTAHFNGLSQTVLNRAEALVRESKVRSQTQVDLCKD
SPYELKKGMSNI
>IRX5b_BRARER
MAFPQGYLYQSSLSLYSCPPYGSTGITAPRTDDLGRSSSGSAFSPYASTAFTNTTVAFSSLQ
YSPESAAPFTSYGGSPYDPSPGMTGSIGYHPYAGPLGPYPFGDPAYRKNATRDATATLKAWL
NEHRKNPYPTKGEKIMLAIITKMTLTQVSTWFANARRRLKKENKMTWTPRTRSEDEDEEDSI
DLEKNDEDDEPMKTAESTKDSETRAEDHDDSTDSVIIDCGEEEENRTESPVPTTSSPQDELS
ESTIKPLSGNCKPTSVIPSPNPKPKLWSLAEIATSDKGREEVSHTSRAGSPQCTFPARHLYY
ASPFFVGFSNYGAFGHLNGCGGGNSTHLNGINQAVLHRTRDSKLTLEMCKELTFEHKRANI
>IRX6a_BRARER
MYGSPYAAAAAAAGQNYANYFPYSADPSAIYSSLNPQYEIKEGSGSLHSTITQPATYYPYDH
SLGQYQYDRYGTVDFNGSTRRKNATRETTSTLKTWLYEHRKNPYPTKGEKIMLAIITKMTLT
QVSTWFANARRRLKKENKMTWSPKNKAGDDRKEDLDSKDSKDEQDLQFSDLDDMEDEDCDKL
DSDCEKSGQDDLPTSSPPKRDCNPDIPLHSNFPSFPCGLKSLGPLNPDYLDHLGSKPQQQQP
SPQSTSINTVALSHFESSEKPRIWSLARTAAAGVVLGTQHVGDVRTGPVDCQMQGVRLPTVG
AVQCGELKGLQDPTNLSNTESLFQEGLQGIHKAYSSGSFKTLQLHSSSYPGLTESCQYSSME
GFPSAGKTETESSELSDTCPTIQEAKTTAFRPVMKR
>IRX7_BRARER
MPASSTGFANFFMDRNINMPAGYQLLGCPPGMQQPPPHLVGMAGMPLPFSGIPGYSFIPYPH
PGHMAHISNSYDLKAASPYHQALLGRGGPYYTPYRPIPADDPSRVTKVATRESTSALKAWLS
EHLKNPYPTKGEKIMLAIVTKMSLTQVSTWFANARRRLKKENRVSWASKGKSDEEDEEEEGE
SEEEGSLRKGTEDEDEIDPQTVDDEDEEDVVVSKSVEHRLAGGLEQQKESDKSDTLTKRESY
KQIADVQKPKIWSLAETATSDIVKKPSDLKHLQTSPDFGQWWAGWPSRDSYSPVNLSTHDLL
KQSQ

Tetraodon nigroviridis

>CAF96727_Tetnig = IRX4a
MSYPQFGYSYSSAPQFLMATNSLTTCCESTGRSIADSGVTGSGQTPVYCPVYESRLLATARH
ELSSAAALGVYGSPYPGGQGYGNYVTYGTDASAFYSLGTFETKDATASAHAGITQATAYYPY
DPTLGQYQYDRYGSMDGGTRRKNATRETTSTLKAWLQEHRKNPYPTKGEKIMLAIITKMTLT
QVSTWFANARRRLKKENKMTWPPRNKGSEEKRYDEDEDGSQEEQIKSENNEDETRSRPDKDL
QLSDLDDFDTLESESSECELKHRYHMNAHMSTTNDCPAGHLIKDSSLKIPIPALEQDLSKSC
LKTSAEDFQTVSAQPAKGCYNQQQQHQMLDGKPRIWSLAQTATSLNQTEYPSCMLRCQPPS
SSLSPPPAATSPVTGLDDRQDSPVTTLRNWVDGVFHDPLFRHGTLSQA
>CAG05430_Tetnig = irx5a
MAYPQGYLYQPSASLALYSCPAYSTGVISGPRTEELGRSSSGSAFAPYAGSTAFTSASPGYN
SHLPYSADAAAAATFTSYVSSPYDHTTGMAGSIGYHPYATPLGTYPYGDPAYRKNATRDATA
TLKAWLNEHRKNPYPTKGEKIMLAIITKMTLTQVSTWFANARRRLKKENKMTWTPRNRSEDE
EEDENIDLEKNDDDEPNKPTDKGDSTDTEADHKLVNPGDMGCDRFKEDTHGKDTDPLLSDSE
FKEQEERTTELLPDSAKATTSSPPAVPRGNQAVAQQDKPSDLSHAPSTVTSNVTSVIHSPPS
APKPKLWSLAEIATSSDRCKSSSDASPSCPGLPQSAVMGTNPSPSRSSPQCPLPNSTVLSRP
LYYTSPFYPGYTNYGGSFGHLHSTHGSVTTGSTAHFNGLNQTVLNRAEALVRESQRVRGQ

>CAF99496_Tetnig
MSFPQLGYPQFLGASHEVYGGERPASAREGGTEGAVNSPAAAASVGSMLGMYGSPWSPHNYS
AFLPYSGATDLALISQMSSQYELKDSPGSHPASLPVHAAQGFYPYGQYPYGDPSRAKAATRE
TTSTLKAWLQEHQKNPYPTKGEKIMLAIITRMTLTQVSTWFANARRRLKKENKVTWGRSAED
RDGRIFSSDNEDEHGKNGSDDEDEEEIDLETVDVEKPEDQRTGEQSTGKEKRETEPGAKEQA
SEQKGLDSSRTLSAEGLRGIEASVSLSRSPVVKLDNSTGRQECQRPQQSKPKIWSLAETATA
PDSSYKLPPASHAHHPAFASAGHPALIPGHGIYTCQIGKLHNWANAAFLNANSLLNMRSLLG
EDSENRNDSLKSPFKMITDRCFTRLSISDVIGVDTENQPGSHRSNLRKLLPPPASRDCQ
>CAG03859_Tetnig
MPAGYQIPVLGCPPGLQPQQQLAAMAAGLPVTYSGLQGYNFVPYAHHRPVTHMNGFDVKGVS
PYHHALLARGAAFFPPYRHGAVEDPGRVAKVATRESTGALKAWLNEHLKNPYPTKGEKIMLA
IITKMSLTQVSTWFANARRRLKKENRVSWASKGKSDEEDEDEEQEGESDEEEEGALGALDKR
NQDEPEREDGSERGESAQESSAPVESRHRQPELGEETKKSDSDGPAVGTKENSANQKPKIWS
LAETATSEPVRRAPDSSPG
>CAG14759_Tetnig
MSFPQLGYQYIRPIYPPERQGIAGNARSGTELSPSGALSNVLSSMYGSPFAAAAQGYGAFLP
YSNDISIFNQLGAQYELKDSPGVQHPGFAHHHPAFYPYGQYQFGDPSRPKNATRESTSTLKA
WLSEHRKNPYPTKGEKIMLAIITKMTLTQVSTWFANARRRLKKENKMTWTPRNRTDEEGNVY
SSDHEGEEGDKREDEEEIDLENIDTENIESKDDLDDQDDLHSDIKLDGRSDSEVSDGYEDLQ
GPEQRFLKAVGKEGKEVERGGEHFHSHQHHHHASLDTKAPQPNGEQLKLNAVSAGSPPSENN
PVPAQKPKIWSLAETATAPDNPRKSPQMNGSSPAGPAAQTIIGPHRLISSCPVGKIQNWTNR
AFS
>GSCT00025552001_Tetnig = irx5b
MWWDSPRAAAAAAAAAAAAALGSFAGAGYDPSSGISGSVDYHPFGPLGPYPFGDPTYRKNAT
RDATATLKAWLSEHRKNPYPTKGEKIMLAIITKMTLTQVSTWFANARRRLKKENKMTWTPRT
RSEDEEDDLDLERSQDEEEPLKMDEGPERRSRS
>GSTENP00013878001_Tetnig = irx2
MHRDGRWRGRALGQPMAASSALLSPFQLRAVSPDSNRAMSYPQGYLYQPPGSLALYSCPAYG
ASALAAPRSEDLARSSSGSAFSPYPGSAAFSASAGAGFSSPLSYTADPTAGFPPYMSSPYDA
HATGMAGALSYHPYGSPGYPYQLNDPAYRKNATRDATATLKAWLQEHRKNPYPTKGEKIMLA
IITKMTLTQVSTWFANARRRLKKENKMTNYTNYGNFSPLQGQGILRYTNSSGVNLAAAAAAA
AANESLGVSQQALEVSTNTKSRPGSPLVKNNPNQIVTVEQQQQHFRPAHLEGKKGT
>GIDT00019750001_Tetnig = IRX6a
MEGEEEELKEGGRKRMGRSELNAALGMYSSPYAAAAAASQNYANYFPYSTDPSIYSTLTATY
YPYDHSLGQYQYDRYGTVDFNSTARRKNATRETTSTLKTWLYEHRKNPYPTKGEKIMLAIIT
KMTLTQVSTWFANARRRLKKENKMTWSPKNKAGEDRKDDLKSEQDCANKDSVDCKEEKDLRL
SDLDDMDEDDCDKLDNDCEKVAVGDEELQRAMAASGGSQKKDCSPDLQLNLTNNFHSFPCAI
KSVTTLPPLPSDFLDPVASKAP
>GSTENP00017591001_Tetnig = irx4b
MAYSPLGYSYSTTPQFLMTSNSLAGCLEPGTPPSHSALRSTSQLAPAIGVYSSPYPKSQGYY
NTSEATALYSRGTLDLKDRAPSGPVTPQPSAYYPYEYPFGQYPYERYSYSCADGASRRKNAT
RETTSTLKAWLQEHQKNPYPTKGEKIMLAIITRMTLTQVSTWFANARRRLKKENKVTWSPRA
GKSSDDRGCEEDSDDAEKETSNDKDIPDQQCTDLQSDLEDFDLLESDGSDCEPRPQFFSEEG
GASKVTPLSHGHVTETLTKKDGLSPECPKLTPVHQQNSSFYPSPGLQNAEPKPKIWSIAQTA
VSLEAEYPPCMLSSTGSSSPAYASNMSLTKGDGQQESPVSTLREWMDGVFHGPPLQHH

Petromyzon marinus
>Irx-a_Petmar
SLRSKGSHYEIKDSPGLQHSSFPYPTAAYYSPYGGFQYTDPSRPKNATRESTATLKAWLNEH
RKNPYPTKGEKIMLAIITKMTLTQVSTWFANARRRLKKENKVTWAPRSRSDEEGHSCGSDAE
VDRKEEDEEEEDEEDEAVGEEEAEEEDDDDDEEIDLENIDIEKCEEEEDSGDLSRDERDGAM

EAELKMREAVKGQTAPGMRTSPASCLSSEAPTGSKPKIWSLAETATSPDS
>Irx-b_Petmar
GAQYEQLKECPAMSHPVFAPHPALHHLPPHYYSPYGPQGPTGGPHGAAVVGSDSGGRLGSKA
ATRESTSTLKAWLSEHRKNPYPTKGEKIMLALVTKMTLTQVSTWFANARRRLKKESRGGGGG
GGGGGAGGSVAGEVGCWGPPGGGLGGGGGGGGGGGGGGDSFHNRGDARTDSEEEVDLQSVEV
ARRRRPLATSPSGSAEVDVVDEDDAAADGDCARDVDGLVE
>Irx-c_Petmar
MHCWLRTSSPYDAHPLTYHPYGGASHPTLGPVGYPYGAGGCGVADPAFRKNATRDATATLKA
WLNEHRKNPYPTKGEKIMLAIITRMTLTQVSTWFANARRRLKKENKMTWVTKARSDDELEDD
DNDGSGGKELGDSSERSDRDGDLILNVSEPASESQDDRVHVIAVPGDGGGGGGGASKCDAAS
AHRAGPGCDMSDGDDERLFPTSATGGIGGATVVLVTPPPGSQQQPASAPDRPSLVQHTSAAM
NASPSILTTSPVAGTEAPRLGRGSPHHNHHHHHHQQQQAAMQAAGIPCMLSAAAAAAAAAAA
AASPSSTSSTQAHTYYTCASPAYFSRYGVAGYAANFGALPGAGGGGVVGGGGGGAGSGGGGG
LRGLPAPPGNGLVYQTVFKSGHRVPPPAYLFAGYGGGGVGTGGGGAEVRILETQETHDGKEM
IALDCKREQAVSLNIAVPHTHDARHAVHYSMQYIPVLRECNTVINYSRGVLW
>Irx-d_Petmar
MAMYKYGAAEANVTRRKNATRDATATLKAWLNEHRKNPYPTKGEKIMLAIITRMTLTQISTW
FANARRRLKKDNKMTWTPLGSHSRDEKQPECDSSHADQHEDNGDDSRLMDEGDEGRGDDHRR
IFSPATMSKRSVSPTGRHSSSNSSSSSDSSESNLALTVQQHAPPIPNSCRHHHSDGDDDKQH
LAGHSNGGHCGVTGVGGLIDGATWEGRAEQKPPPADKPKIWSLAHTATAQMGEQSRWSPRPG
SVSALPYFVPPPPPPPPQLAVESGGNPSTGVSPVEGLRCWASEAYQGGFSAAGDLLWGGDGGE
RGSSFWTDGGGLRFKLAAERGTDGAGRS

Ciona savignyi
>irx-a_Ciosav
MKCKRIRNKRQDVSGHFASTFKFAIDYNLRKRKGLACNFASYPKFNDQPKAVSIVLKGALLR
QNRSSANQSEAGVEHQPDSYRLWTTDDNTKRFFLIALWGLELLIENHSFLAWSEWSRIDEYS
NINTSIEANVKVLITGVIDRRWGVRLYDTLSNKCPINAVGRAALSGSSETKAKKRIRRTVVT
ISRTERMLIGIKCRVSPYTDLLFKRNVELQSKLCKSDLLLNGGTVVGTSPFIMHRIPHDNKS
PAFPVPPIKRLPSNFTVNFAPEAKLRCRSDGQHDIVLGKSTQHYLPRVDFNLKTPLKMDSRK
AVGIEQCSLHGELKRILLVQVAVQTSHVLLRFVLWRKELDYKASENNGLFRRKRDRPHPLGL
GPNENSKPGYFGDSTVSPSPLVVSVSSTTHHAGDPTSQIYSAGEGCFIPGRMQSELPGLNMY
AAPTGSSALSSPANYGGVPAAVGSAPGLVPPHYGSNGMDHSPYYSSLAPTYDMKPGGGDMWG
GSYGQAPPAYYAYESNFGGQYPYDRYCGVDMNDGVRRKNATRESTNTLKAWLQEHKKNPYPT
KGEKIMLAIITKMTLTQVSTWFANARRRLKKENKMTWVPKNRSNENSGSTGEKNIDGDEDDV
LSQNQDSDGKVDADHSLDSDDSTEREVTGTTEHDITNTPYSRDSGVFGGEKSDDLNVSDQPE
DNLAQKDFTNSYQPANDASFERPSLAAAETVQNRYAANDAARIWSPINENGNASSYSNKCAE
NPADSQLNLPSSFRASYNSDSFSSMSRQQQTSSVVSPALMSCAELPGRVVECRYPSVQNWVD
GVYNTTIPATPETETAACHATSVERRNSMDGRIDERLHGVSSPLPDYHHQFQCKQLSTHNS
HLSTHDNDQYNYHTRRAPMDNGRSPVNNIPGQVAPPSYQMFGFNNQIQPFNTSDYYHDERLS
QSTGDARIGYYNPQRTTEPRQNMTVQANRLQSSNMYHPGETKASMLSSPSSASGSSPASRQL
CQGESSVATRHIPSSYHDGSGMVVSASNPSAFHGNYYHMSGDQSDRVHTHIANNSSANNYML
GTSALDQRNMEASQGYQNVLNNQGPYSRDLVAIIIYRLTITPTYRINVTYHERVNTLSYETR
IKSKYIACNNTPAMCT
>irx-b_Ciosav
MLVAQPLDVGLNKTGAHARRISTYSTGEEFFVPGQTQADYSGVTMFPSQTVPPSRGAVSNYC
SGPSGIRPGMPPTEYGSPGVNHTPYYSSNAATFESTIDTVDTWPSRNFNHQRYTPYNYGSHY
SYDGYSQEMNDPSGNRKSATRESTNTLKAWLQEHKKNPYPTKGEKIMLAIITKMTLTQVSTW
FANARRRLKKENKMTWVPKNRSNETTSSDKKVTTDTNDETASDTDDFDINPDTNDFHKMANQ
LSDLTNTKSISTEPHNLSAQPSGTSLVSSPRQSSSIYQSLAGTATHTQSLYCSKSIKSESNW
SPINENGNASSYSNKCVENPADSQLNLPSSFRASYNSDNFSSMSRQQQTSSVVSPALMSCAE

LPGRVVECRYPSVQNWVDGVYNTTIPATPETETAACHATSVERRNSMDGRIDERLHGVSSPL
PDYHHQQFQCKQYTNEPMQSYNGYQSSDNFTNNIQTYHRPMHNQATPYPLPDQSINSVHYVN
QQRVPLQHASAHINSDFSYNATSLQVTPSSASGSSPASRQLCQGESSVATRHIPSSYHDGSG
MVVSASNSSAFHGNYYNMSGDHGTGSNLINTCSPKYNLPMDFPVQQDNLYSTVLCVHTSFTR
IFDVKSAPLIALLNSRCANAKLASCD
>irx-c_Ciosav
MYIKTCIDSPGPPVQNVEASSINSGRNSNQSSLCSDIASRCNCRINNGRFITKKQGQSTVLY
NFKIRTCDELVTKIVSITNRLLEFVDRWPNLRSRTRKGTNRREDSASTQSHLSYISDDKSSG
TRSSDHDGNAKIVDAARSTLAEQRSPGNGIHFPADDRGIPGRPADLTSALFGLPYPVPAPAG
LNPPIYSSLPPGIDAATLHSTLSRIDPKELSAWGNPMAPTHLYPYDQALMYQHSGYGLSLAD
GARRKNATRETTSTLKAWLNEHRKNPYPTKGEKIMLAIISKMTLTQVSTWFANARRRLKKEN
RMTWSPRNRCGEDNERESEDEEQERVGEGNKSYHIASCPYAGVFSRVCGSKYVCARSLSREL
NTANYNRAPREYSCPCKYRVTSLASWRSASVFNVSSTCSSTNSRLYKEGIEKCDLIDKSPID
AMGTLNNLRFVRFRCRCAWFETGVAVSELYGAINREKRDVVGREISDAHYPVNEDGNSPDEM
RHQDRRDNEPNVARSNPTVGPSDLAYQRHHRAHQQQMFLRHLASLPPHLRSVDPFRQFQLQN
QRLAVQGLPRMHPRGFEQPDQQRRSSLSSPSASASSYDRSPSPVNQQRNFRPPPPLNQIGLP
SKSPTRRFRFSPDRVPRDEADDSAKKTEKKPKIWSPVSLTESNSTSQDAPPSPRQREEAPNG
KSPPNPMAVAQAWMEEYQRQLATRNPQLLASTLASIAAKRGVSGGFPFLPYPTALATSRDSP
TKTSRDDDESLTSDTEGEANMPINLSKNGERRNHSSDDATMVIDDVEMKPIMQSIVAS
>irx-d_Ciosav
MSLHGRWIDAFGAIGGGEIRLNISLGGTREKHTTTQSGVFMCQAPCSARYKHSRMVALFPLI
IDGVYTNVCIVFIFAVRIGKYISERWRYRALRVEFAVDYYFNSASQIWNIFTREMAGKSSGS
DNKTVATITFGESREKRSGRDAVSKRERRIGSLTTPKTGATINSSGEIREMDARLVAARIPG
FPMYQPPYGAPAFPGYLPYNPAAAAAVAAATDPSAFYPTMGPGENADVWRAGLSQAAAAAYY
DPSMAAHYGGYGYGPMGLSDGARRKNATRETTSTLKAWLNEHRKNPYPTKGEKIMLAIITKM
TLTQVSTWFANARRRLKKENKMTWSPRNRCGEDDDDDDVTDDVGNHGNTSRSSSPVGGADDR
DFGRLVEMGGDLSDGGFSDAREDRRAKEEEKRKSPPSLEVESATVISSNTVSIPTLTRDLHQ
QSELTYPHLCKRVPESRSRLTFKFQAAKRRPTSTAGQFNHAAAASLIAASRGHPLPPHVMAA
LNEQAQRHYQHALQQMPNYPFHGERALSTSSNASPSPSASSTSPPTSSKPAETSTSSTPPKP
KIWSIADVATSRDSPKHTPHHPKKAPSLSPPTPHCENKDIPNVQQWVGGMLQHKLAMHPGNP
RLMGLSPFAPRCKSSPKRDKAATFVPPVLRHHRCSARKIRQRFAQVAELRQNQYYVRKITSV
TRRTNGNNARKSRFRFGVNRCVNARLHQNLAKYLERIRGELELDTNRTAGWREKLKSGGAGL
KHIRCEAATSAHMRSWPEVRAAGWPGSNCRRMIRLHPMTNQPHPINSFEVSDLHRFIRCRRR
IAEAKAAQLIQDHVSTFIRVVFYFKRRPLVSQNLDALLCFSMRERYRKVMGRRKCYYDVDTQ
PNNDLVDGTKLYRCL

Ciona intestinalis
>Irx_c_Cioint
MQSELPGLNMYAGPGGSSALTSPANYGGVPAAVGSAPGLVPPHYGSTGMDHSPYYSSLAPAY
DMKPGGTDVWGGSYGQAPPGYYPYENTFGSQYPYDRYCGVDMNDGVRRKNATRESTNTLKAW
LQEHKKNPYPTKGEKIMLAIITKMTLTQVSTWFANARRRLKKENKMTWVPKNRSNENASSTD
EKKIDPDDDDSNSQNQDCDDKIDADQSLDSDDSAEREVTSTNDNDVTNTPYSRDSGVYGGEK
SEDLNVSDQNDQQLGQGKDFSSTYQQPSDVGFERPAMPTDTVQRYGPTDASRIWSPINDIGS
GSSYPTKCPDNVGDSHLNLPPSFRGSYSDDQFSAISRPQQTPSAVSPSMISGVDLPGRIGEC
RYPSVQNWVDGVYHSAVPPTPDPDSTTCHPISAERRDSIDGRIEVDRLQGVSPSSPHFQHQQ
FPCKQLPTQNPHLTVNDNEHYTYHERRVPTDNVGSPGNLHGQVAPPSYQMFGFNSQLQGLGT
GDYYHDERMAAQSSAEARLGYYNPQRSADPRQDMNSQQQQGRIHNVYHPGDPKPSVLASPSS
ASGSSPASRQLCPGEQSLASRLVPSSYHANPGMSVSATNPSTFHGQYYQMSGDLTKRGHSHT
SNTPSNNYMMVCGNGVDQRNMDATQGFQNPLNTQHNSYNRSPLPSNSPHTELIMSPRDGDEL
REMQAAETLTNLSTR
>Irx-a_Cioint

MSFLPFPVAFSSNPSMTSRTSLSEAQATISDPGRSGLTEQRSPDNGVHFPAEARGIPGRPTD
IASALFGLPYTAQVAHGRLVPPVYPALPPGIDSAALQSTLSRMDPKDFPSWGNPLHPGPFYP
YDPTLMYQHSGYGPMGLSDGARRKNATRETTSTLKAWLNEHRKNPYPTKGEKIMLAIISKMT
LTQVSTWFANARRRLKKENRMTWSPRNRCGEEENERESDEEERGEMREEGNSPNEMRRTEPR
DSDNSNPGTNLSGSNSDLAYQRHQRAHQQQMFLRHLASLPPHLRPVDPFRHFQLHNQRLATQ
GMHLLPGNSRSNDHVGALKHDQQRRSSLSSPSASASSYDRSPSPVNQPRNFRPPPPLNQITS
PSKSPSHRFRFSPEPTERGSDEENKKTEARDSKKEKIWSPVSLTETTDKPRSITSRDSPPTP
RHTIESPVGKSPSNPLAVAQAWMEEYQRHLATRNPQLLASTLASIAAKRGSSGGFPFLPFQS
GPQSAVSVTSRMESMETSRDDDSLTSDTEAGPNLPMNLSKNKDRGNHSNDDVPLVIDDVERK
PLMRSIVAS
>Irx-b_Cioint
MDARLVAARLPGFPMYQPPYATPTFPGYLHYNPAAAAAVAAATDPAAFYPPIGPGENADAWR
AGLSQAAAAAYYDPSMAAHYGGCYGYGPMGLSDGARRKNATRETTSTLKAWLNEHRKNPYPT
KGEKIMLAIITKMTLTQVSTWFANARRRLKKENKMTWSPRNRCGEDDDEDDLADDAGNHGNT
SRSSSPVGGAEDVDMRRGDFGRFEASGGDLSDGAFSDGRDERRAGKEEDRRKSPSSLVSRSL
SNDESRSRLANGHFVKHGIHPYSNPRSSPASRPNISPPIQARPGLPFPPNMPPMQAPSSQAP
PHFHGSQFNPAAAASLIAASRGHPLPPHVMAALNEQAQRHYQQALQHMPNYPFHGERALSTS
TNASPSPSTSSRVSSTSPSTSLKPASSAETSTASTPPKPKIWSIADVATSRDSSKDISNRSK
ICHKSHSLSPPSSQSDDKDMSNVRSWVGGMLHHKLAAAMHPTNPQLMGLSPSSQGSKVPSNG
ARYPQSFPRFADIVAAQQEKANAAMREMGKPHHSDDVIIMSERSPTIHETPMEALQARRNSS
HSPVSPQQSDEH

Oikopleura dioica
>Irx-a_Oikdio
GAAGSSDVWTGAGVAAPGAYGSAAAAGYSASHYPGAEAYGSAFGAQYPYGAYNDMSDGVRRK
NATRESTNTLKAWLNEHKKNPYPTKGEKIMLAIITKMTLTQVSTWFANARRRLKKENKMTWV
PKNRATDGEDDESNGLGEENEKEFSIKRRLFDFCEFSQKGGSEDETSLGAPIVRLDDNPAVS
AVQHLHISDLLETQSPSR
>Irx-b_Oikdio
MISTLLNVNYGHCLRPVFTNSGGTQLRRPRGFRRLTQIMSVGSRYSERRKCPIDLDKMGMGV
PQVNPMMAAQYGYAQLMQAQSTRKNATRESTQQLKAWLKDHQKNPYPTKGEKIMLALVSGMS
LTQVSTWFANARRRLKKENKWCPEGGSEDNDGSDSGSVTAEDSASSIEQRVPVISVTDESGY
SSSDRDTGSPPACTVPVAPPALFSSSLLPSFAAAVPLPRYMPRSLETSPVAPQKRKAGSLWS
IADITGSS
>Irx-d_Oikdio
RKQCTLQPTALCRSSDDASVRLQIFRYGSAGRFPSNGHRKNATRENTAPLKKWLEEHMRNPY
PTKAEKITLAIISSMSLTQVSTWFANARRRLKKENRVTWETPRETTGSDEHETEELARETDS
RNENATCPSQVSSPPRRRSPVMSTTMAASAHPSTMPSLAGLRPGLLGVAIHNSVPSASIYQQ
LLMNHILRPVTSMPPLTVCAPTISNSNSSNAKYPETSSQPKRIWSVAEEIERARTESPGSEG
DSDRR
>Irx-c_Oikdio
MICYSNGQTNIILSAESGDELAAIGNGYRSPSTSIHNIDSESGATVVEFNLRSIFKIKPSNL
EIMNPHSLGLTSSLLSGLDQRLLQQQQLLQNPFRLGTHGLDPALIARAQLTSQLPRLPQLP
QVIDPMLAAQYGYSQLLHGAGARKNATRESTAPLKNWLKEHQKNPYPTKGEKVYLALISGMT
LTQVSTWFANARRRLKKENKWSPSSGFEDDDSSTSPNKPSSVTDEESNAVKRVSEDHIESGY
SSHHSLSDQSSEASPERADSPSVSEPAPIRVSAISAHIPVSPPSSLPRPKIWSLAEMTTSSD
EEEELNVN
>Irx-e_Oikdio
MNFGEQINRLHLQSRVPTALSDVRVPAILPGSPLYPSSGFLPTGYPFGGLAFHPQSTIQRRQ
TARESTGPLKAWLQEHPRNPYPTKAEKVMLALISGMSLTQVSTWFANARRRLKKESGNKEDT
SDDSIDGKDDTADESVISVVDIEPTVHVTPSLARERESEQEEATESKEDEPVQEDRSSSSTP

PPTSEISNSTFKPKIWRIVDQIQNDSNV

Branchiostoma floridae

>Irx-a_Braflo
MSSMSYAQFGYTYPSSSPLLMSSHAASCCEAGRPIMSDPHTGQAVCSCQYEGRVAGLVPPRV
GEMSLSSVYGSPYAGQGYIAAFGGDPSAFYSPLGNPFEAMKEGAEAWRANLQQPTAYYPYDP
TLAGGAPYPYGSYAAMDGARRKNATRETTATLKAWLMEHRKNPYPTKGEKIMLAIITKMTLT
QVSTWFANARRRLKKENKMTWSPRNRSGDSEGGEGSDIEGDEDTEREGRDGDRHGSDKDVED
VDLDDIDVDDDKVFEDSTTPGEESKDNEYYERKTGSSSDISPPPHRTIPSESTEDNTREIQR
QRETQASSPSTNSPPPVAPKPKIWSLADTATSSSGPTARPDAPQNSRDHRTITQLVLPAQTV
RQAVRLCSQLSAVVDPVRIGVAQQSSEPDLIRTAFRPVQKRVDEADLPGEEPGLSRRSTRFG
PVPTRPWPWEWARRAPRQRDPGVMARDPCAYFTAAGWIIIGSKLTGDGGCGWSPEPVHHTAG
RSIWVAMVTEEADDLHSRSIFLWDSPSAVAIVTEDADGLRNGFIILLDAGVAIVTKDADERH
RRSIILKDALSAVAIVMEDADGLHSRSILLRDAPSGVVMDRRPTDASQFAHQDKESTGSLQE
KARVLDVLSLVSKFLFLIH
>Irx-b_Braflo
MSYPHFGYSLPSSSQLLMSSHAASCCEAGRPIMSDPHTGQAVCSCQYEGRVAGLVPPRVGEM
SLSSVYGSPYAGQGYIASFGADPSAFYSPLANPYDPSAGLQQPTAYYPYDPATLAAGAQYHP
YGGYGPMDGTPRKNATRDATSTLKAWLNEHKKNPYPTKGEKIMLAIITKMTLTQVSTWFANA
RRRLKKENKMTWSPRNKSGDERREDGSDSENENDDEDGREGGEDKNQEDDRGLEGDRDNQEL
DVGNPCEENRQEDNASGSVSDYRPPPQSSSPADSLGSISAAPRPASPEPSPAANSVPVPPIP
SENKPKIWSLADTATSSSPGLRSEPPRSLPTGAIPTIPGQFPGHRPFDSVANLRHWVNGFSQ
PIRTHAMSSSQPGAATSVFSSGQLPTGVSLAGLPVRTLSATAPTGVHAFLGREHAFPGVART
HSPATNEVSNPTNARYREGPYSEGHPTAFKPVQKSHNQGVKCVQTTGKRRLPGRAGRNNPSQ
CGVHLRTSVTFTPGRPRSPPFSSRLTDRPVVSVHNILSSTCLDLPPPGGAC
>Irx-c Braflo
MIVTVIRTLSFLHKKHAFIYRSHVILRLTLPHGRTRHNKNLYSQAKKTTPLCPRATPGRRSQ
NPDPNTVIFSPSTRHPTFVAQTFIIFIIDAVAKEPAPGVGQLLARRADKATSLRRQICEASL
LMPARASHAPRETNSPQMAVGRPPLARHQKGLLINEEPHIIATLELSKAGELLDRIQETNGL
ARTLSGMSYAQFGYPYPSSSQLLMSSHAASCCEAGRPIMSDPHTGQAVCSCQYEGRVAGLVP
PRVGEMSLSSVYGSPYAGQGYIASFGADPSAFYSPLANPYDPSAGLQQPTAYYPYDPATLAA
GAQYPYGSYGAMDGTRRKNATRDATSTLKAWLNEHRKNPYPTKGEKIMLAIITKMTLTQVST
WFANARRRLKKENKMTWSPRNRCGDERREDGSDNEDDDDDEDRKDREGNSDDDNHGGLEGDR
DNQELDVGNPCEENRQEDNASGSVSDYRPPPQSSSPADSLGSISAAPRPASPEPSAAANSVP
VPPIPSENKPKIWSLADTATCSSPAPRPDIIKTTLQGPVHMQFSHHRPFDSAVTNLRQWVDG
VFHRPIGSLPAGPMGPLPVASQASASATVSGMHPSSNPHTTPPGFVSSQERTPALPIALRTH
ASPAKDAPVVESAFQGFGMIMIEWLRMTTTSPGIDRQNHNERANVSX

Leucoraja erinacea

>irx_Leueri
MSFPQLGYQYIAAARPLYPDRQSLSSSRTAGELTASGAASTVLTMYGSPYAAQGYGAFLPYA
ADLPMFPQLGAQYELKESPNVQHPGFPHHHAAYYPYGQYQFGDPSRPKNATRESTSTLKAWL
NEHRKNPYPTKGEKIMLAIITKMTLTQVSTWFANARRRLKKEKQMTWTPRNRTDEEGNSYGS
E

Hydra magnipapilata

>irx_Hydmag
XLSSSYNKYNNESCSILDYALLASARRKNATKETTSVLKSWLNEHRKNPYPSKSEKVMLAIM
TKMTLTQVSTWFANARRRLKKENKMTWSPRKKSNSKNSLKEGSSCSSEPIPEEMDIDIKEQI
FSAQDCNINTNEENSGKNCIETPFSVAQDTKIQRPSVICVLESPTFDQIKNRFSRDPSPVDH
LQKWVDGCSQEKPDDLVCIKDLTPPLTPIDNRVTTFTSQKLFEAFDAAKINKNNDTQ

Nematostella vectensis
>irx-Nemvec
MTSYCEIDGYPFKRLRLVKIDVDFELCLMLHGAGNSCCDSVRQVHDGMPGHSVCPCQFDRVL
QHRVASVPVSVLPRPAFGEAGFSLGSGTGLSAFYSPLARACDMKDSTQPWRDSWYLSQASRA
PFELHGFDPHTHPACTRFAERFSPIDLAAAGARRKNATRETTSTLKAWLFEHRKNPYPTKGE
KIMLAILTKMTLTQVSTWFANARRRLKKENKMTWSPRNRCGEKKDGVDGSDIDDDGDLDITS
DDEEKPSENHDEKVHIQENETENNEKISVIDEAEDKKESDSTRDRESPPREDAKRPNIITSL
VDTTNQTHSLPKSIPSAILISTAQNHVLDSPVKSLRKWVDGCFYTAPVASYMESSGVAGKPL
PSTPKQRTSTEIPVITSAPSSQSAEKPERRTSSESKETRPRSPVEKVKEEEPAKASFRPPEP
SDYGHILSRRGKGEGIKNVLKGADSVNLKVKPTFYIGLVKENGVFPQQRNLSEVYPPKQNLI
ANLFLAERLTDPIEFITHVNSKRPCACARKTARHSELSPCEHPAIANTLLLRSPCYCDHPAI
ANTLLLRTPCYCEHPAIAITLLLRTPCYCEHPAIANTLLLRSTCYCEHPAIANSLLLRFSCY
CDHPAIAITLLLRSPCYCEHPAIAITLLLRSPYHCEHPAIAITLLLRTPCHCEHPAIANILL
LRSPCYCDHPAIANTLLLRSPCYCEHLDIANTLLLRTPCYCEHPAIANTLPLRSPCHCDHPA
IAITLLLRSPRYYGHKYNARGRK

Suberites domuncula
>irx-a_Subdom
MASSPNYNRSENGGLNHLPAASLPLEENLVARGSPSHIENGIPGEHTGRAVVNGQSPSPQRS
TCSGPRKPRNPAVLVLWIEEHSANPYPTKAEKNFLAHYAGMTTRQLNDWFANARRNIKKIGY
ETWKEKHSAYSACFSMLRPPTADGSRFTRLAQFPMEIAGGLNGVGPCGSDGLKLASINYGRK
DAPLPQQAAAPTLSVPPNTCGNINSRPFPYLLSPQSSLMNVGGTHSQTANTLRQVAMNPAAY
YEVQHPSAGASIRPVQPASGISLPPSGISFRPMGGANVSLFSSTGRPGSMPPQFTPSPISIA
TPWTDNAAVSDASVVKQERGSVINKPHSPPVQLHVSPLLVSTHVQRPPSPKDSDTTSNNSPH
PATDLRPNFASPTTAESTWAATLPLASSSQHLFEDSISGGVGTQLGN

Amphimedon queenslandica
>Irxa_Ampque
MSDQHSLAGHSHHHLGSSSLKLSSSSSPSSSSLATGGSGSSGSLSGYSAAGSITRRMRNTAV
LVKWIEDHQSNPYPTKAEKQYLAYYSGMNMTQLSTWFANARRRIKKIGMKTWLEGRSTFSLD
YFPTPRASSIGSLDSFTAGSYHGLSSSPYASAAAATYGALTNGLHSSYPSQQNTLLSGGSGM
TSHLTNGLGGTSYDSSTLQPTSSTTPPLSGFYGTRSMSLGMPALPVGQWPSTPILSSPPVSF
HSRPSSGNTAPLHSPLTAQVYSADTSLRTHSAQSIDVGDSSYSSLQQHSPDQMLPECASTEP
MLSDHKSGVTLQN
>Irxb_Ampque
MRTKCSFENKIRERSTMFFTILMDDLAEEEEEKERSMEGLEGSVQLASSRRRRRDATHLIEW
LDLHQGNPYPTRVEKEQLVVISGMNFKQLNDWFANARRNIRKVGFDRWRKNKVTVGGIGGYG
VHQKHSYVLPEDNGGGGKGRRKEKRSKGGGEDGGKGDIDFNLSPPNVISTRGMQPYYYSRSP
HSLVSPHQIQLPAPRPPPPPPSPRARLDCKTAGCGQLPGLSSCPVLHHHPFSHQVQQHVPHP
IDIGYPLGMAQSTSLSWSSLGLPPYAVGDSWLPHPVQSTTSSLQQQQQQCGYSWKECLKDED
KDSLS
>Irxc_Ampque
MILKGPGRVGASMIGEERGSIQRVTTLFIMSIRINQRETTHHHQQGSKLRNGISLNMSETKS
SSQGSHSVADGSKASQEKGSSSPGSWRNTDVLALWITEHLQLPYPGKVEKQYLCFYSNMSMK
QVSTYFANARRTIQKKQKNIKRRLSKGSSLFNLFEIITNPKKKINKNNPGSEM
>Irxd_Ampque
EKGSSSPGSWRNTDVFALWITEHQQFPYPSKVEKQYLCYYSNMSMKQVSTYFANSRRNIKRR
L
>Irxe_Ampque
RNTYVLEKWMQEHMTRLYPTAEEKQCLAHFSRMNTKQNIAWFSHIGSHISH

Saccoglossus kowalevskii
>irx_Sackov
MSYPLGYPYPTSSQILMSSVSHSGTSCCESGRPIMTDPHTGQTVCSCQYDASRAAIMHSRVA
GLPLMYGSSYTAEQGYVPTFGTDPSAFYSPLNTTYDLKGSAEGGWSATPYQPTACYPYDPAY
QYYGDRYGGVDINGAARRKNATRETTSTLKAWLYEHRKNPYPTKGEKIMLAIITKMTLTQVS
TWFANARRRLKKENKMTWSPRNRCGDDRKYDSGDEDGDNENDDDKIDIKDDDEILSDRFDGL
DTDKEXDDKDNSDDEDQDHLGGKAETRHSSANEDSLSLSPSPLLSQNIGTNSSLLTPATSTT
SQQQQPPQPPTIKPKIWSLASLAGTSSSPTSPEMSRRSPLLCHQPSASTFHQPHLNTEPNVS
SLQHWVDGVFHHHHHHHHQQSHLTYSSTSRTSAATSVLSYTAQNSLLGNEHSALRSPPGSSN
MNGDHLPPGGPCRDLAMIAHNSAEAAKYSVAALHPSRDDDLREKSVVHPNDS

Caenorhabditis elegans
>irx_Caeele
MIPIANQVKMEEPTTPQTTTPAPPTSAPDALRGILPPNLLGQFHFHPAMAQVLAAQAQAAAA
AQQATLAGFPGVPMFPHGIPADLKPEMLLGGGPGPMPMFFSDAHRLYHPYGLDGIKKRNATR
EATAPLKDWLHSHRKNPYPSKADKVMLAVGTGMTLTQVSTWFANARRRLKKENKMTWSPQNR
RGDGCDDDEDDDDDDMNRPSSSTSINSERKGESLFGKPHLASPTPSGGSGGSDELTTPKKEL
VSPIPNDDSESPKRKPKMWSIADVTSDDDSSKKDSPPSSDENLLTGSLFQNAMKKQMEVMAR
LDPTLFRHTMHPFMFQLLAQQAAQGQQLALLAPQTTPPNEAGEVSR

Caenorhabditis briggsae
>irx_CaeBri
LKVEEPSTPSTTTPAPPSADALRGIIPSNILSQFPLHPAAMAQFLSAQAAAAQQPNLVFPGV
PMFPPGIPADFKPELLMAAGGPGPMPFMFGDAHRLYHPYGLDGMKKRNATREATAPLKDWLH
AHRKNPYPSKNDKLMLAVGTGMTLTQVSTWFANARRRLKKENKMTWSPQNRRGDGCDDDEDE
DDDDMNRPSSSTSINSERKGESLFGKPHLASPTPSGGSGGSDDLQTPKKEQNSIPDDSESPK
RKPKMWSIADVTTSDDDSSKKDSPPSSDENMTSNSLLIQSAMKKQMEMFSRIDPNIFRMNPF
MFQLIAQQAAQSQQISLLPSQTTPPNEAGE

Pristionchus pacificus
>irx_Pripac
FHPYGMDGVRRKNATRETTAPLKGWLSDHRKNPYPTKAEKVMLAFVTKMTLTQVSTWFANAR
RRLKKENKMTWSPRNRPGEDDDDLADLERPGSSKSDLSDDHNESNNNENNQSPNGVEDSPRK
PKSLWSIAD

Brugia malayi
>irx_Brumal
MKPEMIGAVRAVSGYFFDPMPYHPYASGLDGTRRKNATRETTAPLKSWLNEHRKNPYPTKAE
KIMLALLTKMTLTQ

Trichinella spiralis
>irx_Trispi
FSSIFRFANPLDLNAARRKNATRETTAALKSWLYEHRKNPYPTKGEKIMLAIITKMTLTQVS
TWFANARRRLKKENKMT

Meloidogyne incognita
>irx_Melinc
MSHPPPPFSLPPHPPHPSAFPFPLDPTTLAAFQPFMVQVQTEHRRKNATREVTAPLKHWLNM
HRKNPYPTKAEKTLLAVVSTWFANARRRLKKENKMEWSPRERPEDDCDEGDGEEVDVDTEEN
HHSQNHQQQQGMSDFLMLMSGRHQQQQNLLAQQQWAMFNDQNKNGGIS

Strongylocentrotus purpuratus
>irx_Strpur
MTSYPQFGFPYPASSQLLMSAAAPATTCCESGRPVASDPHPGQAVCCPPLDSRAALLHSARV
SGLPTALYSSPYAAAAAEQGYVHFAADPSAFYSPLSSAYADLKGTPEGYAAALAAQQAACYP
YDPATYQYYGDRYGMDLNGARRKNATRETTSTLKAWLYEHRKNPYPTKGEKIMLAIITKMTL
TQVSTWFANARRRLKKENKMTWSPRNRCGDGTSEDKDDCDSVGDDDDDARDIDVDVDGDDLD
SKLDGESRLLGKDMDDDHHRDHRDDEDIRMRLHESARLGLVMRDSDVMADRMDSGPKDSEHG
ALRDDYRRRSGELLAEHHRLSGLNGLDPLRLNTSRDSNPRDNSPDSLQLSRPSSPVRVCGSA
SPSHDRATPIHDDVNDQDKSSNSVPQNKPKIWSLANTATSSTPAERSRTLFPSGLNGRHPAI
AFASPYDSPMSSLRHWVNGQFHGIPLPPGFPLHHSQLALAAGMAHHQFSHPQGTQGAPTSVV
SSAQAPLLATTPHHARYTMSKELASHQSGITASILASIQHQRESESRQHSPSHSSEEMPMRT
AFKPVQKRPILVSERPSEAAGSPESHREPHREPIRRPIGLSIERLTC

Trichoplax adhaerens
>irx_Triadh
MSYSQFACSFPPQVLMHGHTHSPNHFCSSVPQMTGSTNVCQCQLEKLYMQQKLSPTPYQSMM
HSHQCTANGFPSAISNNSDKPMIYPSMNSPVQGDMKCGNSSIDACYQWYMSQAGKYPVGHVD
GISRFNPIDLNNARRKNATRETTSILKAWLYEHRKNPYPTKGEKIMLAIMTKMTLTQVSTWF
ANARRRLKKENKMTWSPRNRTGEGRKNSEDDSQDEDLNDDDDDDHTRSDGLSTCADSPASSV
MDDKSDCGRHSPELKLAQEIARRQEKPAIWSPITACSKPSVPCTTTTTLPSSNTNMDSPVKS
LRKYVDGVFHLKENEVEINVETKVEPASPESPKEKVSVSVEMKSEKEEAVQEKSESQYREFD
AALALTTLSSSRQS*

MKX PROTEINS

Drosophila melanogaster
>MKX_dromel
MDKSTRPKRNRRHSRRAWPPEEELHPASRATKRLFTPDIKRMLKDWLIRRRENPYPSREEKK
QLAAETGLTYTQICNWFANWRRKLKNSEREKAKKSWGHLIKNYNHNARGNVEQFSISSEDSI
WEEEMHSCPAEDDEGDDNEEFSSHSTGSDGNANNEPSSPYKPILFVGSAGLTERISAQTGGK
TKYKHQMMEKYMRDSSTAKTTSQQGTQLNKWLESAAKFTPDRNNYHIEWNMTRQKASSNSSC
GQAIFTTDVSGAGRGGRWMLHHKDELEAAEALANLAFNCRQRWHHI

Culex pipiens quinquefasciatus
>MKX_Culpipqui
MEKPSRPVRNRKRSRKAWPPGDTHRPIKRLFTPEIKRMLKDWLVRRRENPYPNREEKKQLAC
ETGLTYTQICNWFANWRRKLKNSGHDPVKKTWGNLIKNYNDNAKGNVEQFSICSSDSIWGEY
ITSTTGECTPVDFNANTSSIIEASFPNAYRAENGTYYLTPSTTVSPAVNYRSTNKYKSSIME
KYLPQGPSGLSCELRGPPSLSKWLESTAKFTPSKNNYIDWDQPG

Aedes aegypti
>MKX_Aedaeg
MEKSSRPVRNRKRSRKVWPPGDIHRPIKRLFTPEIKRMLKDWLVRRRENPYPNREEKKQLAY
ETGLTYTQICNWFANWRRKLKNSGHDPIKKTWGNLIKNYNTNAKGNVEQFSICSEDSIWGEN
GEDSQGSRSPPSGNFSKRSYPTYINGNHSSNNNNNSKAFVNYMKYMERRSSDTSEDDTPGTY
PGNMFIDHCYTPNNYNAVFGLPVQPQCFQVIID

Anopheles gambiae
>MKX_Anogam

MDKPSRPVRNRKRSRKAWPPGDSHRPVKRLFTPEIKRMLKDWLVRRRENPYPNREEKKLLAV
ETGLTYTQICNWFANWRRKLKNSGNDPIRKTWGNLIKNYNTNARGNVEQFSICSNDSIWGEN
GEESQDSRTSSSGEHPYTNRHRHHHHHRYHLGHQRQGQKYESNDNPGNPLPYHVYAEAGDCA
GDNNNSASCRNNNLDCRPFAEYMRVPEYGRIEECPSNEGLRDSYPPT

Bombyx mori
>MKX_Bommor
SFEPMTLIQRSCDTKVDAMDRLTNKENNEKPARPVRNRRYASFKCFRHSFVAGHRPQKRLFT
PEIKRYLKDWLVRRRDNPYPNREEKKYLSRETGLTYIQICNWFANWRRKLKNANGDRHHLTW
GHLIRTYNDRAQGNVEQFSICSDDSIWSEPELCSPDQNSLENEVILTDSPSSGLYEKETEAA
LKDRCDNLNNNSYSFDHDSPQKNPKAVTSPVLLSKWLESAARFQPSEINYSWWADEKRRRPE
AKAQRVALNNMIRHDRDEVEAA

Tribolium castaneum
>MKX_Tricas
MVKYPQDRPDASTLLKSITHNSDADYVRELECKLAERDEEILRLKELLKSAEKMDTRIAEVN
RERSRRPVRNKRYTRRGYGELDKRPPKRLFTPEIKRFLKDWLVRRRENPYPNRDEKKNLALQ
TGLTYIQVCNWFANWRRKLKNAGKEPQRKTWGDLIKSYNSQVQGNVEHFSICSDDSIWEEPD
SDESPPDHSYTTFEHDNNNRYFANSPEREIENPFSQAKYKNHIMEKYLRDCQKPDDDVKPSM
ITKWLESAVNFTPSENNYVHWTFSKNQKKKKKEVASQVCHGREELDAAEALTRLANARAFKA

Pediculus humanus corporis
>MKX_Pedhumcor
FFFFFWIFKMAHNIESDKGDLNKINKFNGRILRNRQYKRPLLDPRPTKRLFTPEIKRHLKSW
LVRRRENPYPNREEKIELAAQTGLTYIQICNWFANWRRKLKNSGKNINKFTWSNLIKSYNDQ
AMGNVEHLSISSNDSIWDETDIDKSDKNQIKRYLPDNDYPDDCPTFFRVGVKNNINNCNLSC
FSKPEQTNKSDYLPQCFQISSTSDFLLKPPKINDNSNKKTNYLKKSRQLNKKFNDTTKKHFN
KPVRDKNWNYKKMINDVNTGTDQKNILRTNNKCRLGNNNSSLLTKWKKSAALFQPNKKKYST
VEFIQKKSNGFLIKFDHFHGREELDAAEALTFLSRASSNNL

Strongylocentrotus purpuratus
>MKX_Strpur
MAEKVRHKRQTMKSMARPLKQWLYHHKDYPYPNRKEKLLLSMESTMTLVQVSNWFANARRRL
KNTVHDPAMSWEKRIHKYNKHVTGNAERLSISSVESSGGDDFDDEEEFNGFESDDSSTVNYA
QDVTTSTNELQGANHNVHNATHPGICSPHPLSSVPHIASHSDHQGQSLPTHKYKHSIMQRYL
NDTYNHAQQRQAEASTVAASMDSSHTLLPEENSLNLPNYTDRTAGRLTGQEWSKFRASGSVN
SHEYERMSTSPETTMSSSIGSYRQRHCSEDYSLSSSSNDWTSFLKDTKSTDFKNEEDEETYW
QEIVAAVALTNMAR

Xenopus tropicalis
>MKX_Xentro
MNAIVFNKLSGQVLFEESPNDRERGVRPYLGVMESPNADVGLQDGISIKDNASLRHRRPGSS
RGASGKVRHKRQALQDMARPLKQWLYKHRDNPYPTKTEKILLALGSQMTLVQVSNWFANARR
RLKNTVRQPDLSWALRIKLYNKYVQGNAERLSVSSEDSCSEDGENAPRSHHNEEAYDKPIHH
TVITKESSVIKSGVRAESSASEDYVSPPKYKSSLLHRYLNDSLRHVMASNAAMREKTRQRNH
SGSFSSNEFEEELVSPSSSETEGNFVYRTAQMCFHFHKKSSAVNKEGASKDETYWREINAAM
ALTNLAQGKDKLQGTTSCIIQKSSHISEVKTVKVALLHKY

Tetraodon nigroviridis
>MKX_Tetnig
MNAIVFNELSGQVLFEEKANEVEMSSRNYLDVIDGQHPDLLSSKQTMRESQAMRHRRSGGRP

SGGKVRHKRQALQDMARPLKQWLYKHRDNPYPTKTEKILLALGSQMTLVQVSNWFANARRRL
KNTVRQPDLSWALRIKLYNKYVQGNAERLSISSDDSCSEDGDNPQMAQSSSEELNKPMYQSV
IKKEGSAMVAMGAGLRSAAEATALGEDYVSPPKYKSSLLHRYLNDSLRHVMVANAVMEARKR
NHSGSFSSNEYDDELLSPSSSEAEANFVYRAEQHPSIKNSPACCPALFAPFLLSLAPSPRWD
VKVVQKEKMKGRDETYWREINAAMALTNLAQSKDSTSGTTSCIIQKSSHIAEIKTVKVPLMQ
KY

Mus musculus
>MKX_Musmus
MNTIVFNKLGGAVLFEDRGTPDRERGSRTFSGFLDNPHTGPEVGIPDGPPLKDNLSLRHRRT
GARQNGGKVRHKRQALQDMARPLKQWLYKHRDNPYPTKTEKILLALGSQMTLVQVSNWFANA
RRRLKNTVRQPDLSWALRIKLYNKYVQGNAERLSVSSDGDSCSEDGENPPRNHMNEEGYSTP
AHHTVIKGESSAIKAGGRPESRAAEDYVSPPKYKSSLLNRYLNDSLRHVMATSTAMMGKTRR
RNHSGSFSSNEFEEELVSPSSSETEGTFVYRTDTPDIGSTKGDSAANRRGPSKDDTYWKEIN
AAMALTNLAQGKDEVQGTTTSCIIQKSSHIAEVKTVKLPLVQRF

Homo sapiens
>MKX_Homsap
MNTIVFNKLSSAVLFEDGGASERERGGRPYSGVLDSPHARPEVGIPDGPPLKDNLGLRHRRT
GARQNGGKVRHKRQALQDMARPLKQWLYKHRDNPYPTKTEKILLALGSQMTLVQVSNWFANA
RRRLKNTVRQPDLSWALRIKLYNKYVQGNAERLSVSSDDSCSEDGENPPRTHMNEGGYNTPV
HHPVIKSENSVIKAGVRPESRASEDYVAPPKYKSSLLNRYLNDSLRHVMATNTTMMGKTRQR
NHSGSFSSNEFEEELVSPSSSETEGNFVYRTDTLENGSNKGESAANRKGPSKDDTYWKEINA
AMALTNLAQGKDKLQGTTSCIIQKSSHIAEVKTVKVPLVQQF

Gallus gallus
>MKX_Galgal
MNTIVFSKLSGQVLFEEDAKERERGGRPYVGVVEGPHHAEVLLPDSPSIKESLSLRNRRTGA
RQSGGKVRHKRQALQDMARPLKQWLYKHRDNPYPTKTEKILLALGSQMTLVQVSNWFANARR
RLKNTVRQPDLSWALRIKLYNKYVQGNAERLSVSSDDSCSEDGENPPRNHMNEGGYNKPVHH
TVIKTESSVIKTGVRPETSANEDYVSPPKYKNSLLNRYLNDSLRHVMATNAAMMEKTRQRNH
SGSFSSNEFEEELVSPSSSETEGNFVYRTETLENGPNKCESAANRKGTSKDETYWKEINAAM
ALTNLAQGKDKLQGTTSCIIQKSSHISEVKTVKVPLVQQF

Brachydanio rerio
>MKX2_Brarer
MNTAVFSKFDQLRFEESGQNVERSSRSYLELIDGQRSDLLTSHKVMDGNDALKHRRNGGGKV
RHKRQALQDMARPLKQWLYKHRDNPYPTKTEKILLALGSHMTLVQVSNWFANARRRLKNTVR
QPDLSWALRIKLYNKYVQGNAERLSISSDDTTSDDGENNLQMQASESDYNRAVHKSVIQDSR
VKVGRNADPSVCDDYVSPPKYKNSLLNRYLNDSLRHVMASDKVMDSRKRNHSGSFSSNEYED
DFLSPSSEAEANFTYHTVTLDCGTRQSESGARKGGNNETYWKELHAAMALTNLAQVKESSAA
GTTSCIIQKSSHIAEIKTVKVPIQPRH

Brachydanio rerio
>MKX1_Brarer
MNTIVFNKLSSQVLFEEKANEVERSSRNYLEVIDGQHPDLLSSSPVLKDSSAIRHRRSGSRQ
NPGKVRHKRQALQDMARPLKQWLYKHRDNPYPTKTEKILLALGSQMTLVQVSNWFANARRRL
KNTVRQPDLSWALRIKLYNKYVQGNAERLSVSSDDSCSDDADPATRAQVGEFSKPMYQSVIK
KEGGGMGAGLRAASDASLADDYVSPPKYKSSLLHRYLNDSLRHVMGANGVMDARKRNHSGSF
SSNEYDEDLLSPSSSEAEANFIYRTETVEHGSNKCDSLQKEKGQRKDETYWREINAAMALTN
LAQPKGTATAGTTTSCIIQKSSHIAEIKTVKVPLLHKY

Lottia gigantea
>MKX_Lotgi
MRRLFPEKIHHKRQTLQDMARPLKRWLYENRDNPYPTKEQKHSLAVNSHMSLVQISNWFANA
RRRLKNVVKKPELSWSERVELYNQYVEGDAELFSISSEDEDFDEDEDVQNDDETFQNENTLV
ISHTDLNSDSLVPSRTTTPTFDQRSTVFASTTSLKHKQTILQRYFNDNAYIDAMGFHPPSEG
DPQRVINVAELVWNRRDRHQSGSLGSQDYEEMSTSSASSRIQHRILDDFPEEQELPTYRITQ
HHSFHDDSDMRQKEMDAVFALTSLANSHRSKLQENVTPERSNFRM

Capitella Sp I
>MKX_CapspI
MSDLDKLQALMSEYLRNGVDAQQLYQYYSQHVMYHHLQQAREKGELKEEVPFQPPITELMLP
KVPSEAKEFESVGADFDNKSSVDRAEDIEVLDSAFAEWSVNLSDSVCGKCSERKDDESYMKR
RRRPVVSLDRVRHKRQTLQDMARPLKHWLYKHRDNPYPTKSEKMTLANVSHMTLTQVSNWFA
NARRRLKNTVRDPELNWESRIKMYNSCVEGNQELLSIDSDDDEEEMKAPPQPTEAEAVASEE
EAATPLDLSDHTVPGEAIPSPAPSPPMHTDEAPHPPKYKQCILQRYLTDSYQHAAQMQNEFP
RQRNASGSLSSHDYEEISTSFAGSPLREHQFDEQQEMAEELPAKVENASRKLSPAESRYSAF
DKELLGAYLSVRHFQPIIERRVCTLMTDHRPLALAWNKQSDPWSARQQRHLSAIAHVCHRSG
ASNTGADCLSRALTPQVSLTSNLPEAVLDVQRSSDDIIAVAEGRSHNPLDNVSRSRHRLCSP
RRQPRLLPHRQRLQSLPLPDDAIPX

Helobdella robusta
>MKX_Helrob
MEDKRNNFIQQYQQSQQQPQTYSRDQPMSNKQQQQQQQFDSKLYIRHNYNRKRHASKRRSS
SSSASTASETGERDSLKRRRFTKQNSTGQSQQPENGKQESENCRDTLRLTDRWSNDESVPCC
LKNKLEMTNNGNDGDKDENEINETNSKRRKENCDTAKQTTPNQKEIKNGRPTAPTENIANIK
EEPLDDYSNSEESQRYQSSPGRQIKPQPQHSLPTFKADFSPDYRPVRPIIPTAPMFPFNPNL
PFHLNYSFFSDYPFPASHPYFLQIMRPSTTPLPNFSLPSSSSPPSASATSPASSSAKCTPLF
SSSTSLTSFSEQQQQKPTTLSSLSTPSSSSSSSSTLVNPPVTPNNTAKLRAWLTDHMDHPYP
TKGDKIMLAVLCKMTLTQVSTWFANARRRMKKGGDGSDDLEEERKDSSSLTLSPPSTFSSSS
LSPLTSSFSISFSSTSTPSTSASTVSSYPDSISHASAFLPQIPSSNCFPRSFDQFSRPCYFS
NIYNPYFTRLPGFNPNLHANHFIDYKPQTQQVPSQNSTNNSVRPPWPAKKRNKVWSIKDILG
SS

Petromyzon marinus
>MKX_Petmar
MGEEEKRMLAAPSSLEGLPTAKRVALWLSSAGSLWAEEQKSAEAQPRLSHARARRAHQTPGH
GVVGIKHHGKAENTVSAVTGAHTNVCDRLGSERIRASLPLSPRPGPAMNVVDVFRHPGPKEV
RLEGRQLSSAHQGALAAHGQLLQLYPAGQSAQRCSGAATSASHLISSPSRMTPGDIPPGPFL
LLAVPTRQCIFKRGGVLNLEAHQLVKHQTDGIVACDRHYGRRRSGALRVRHKRQALQDMARP
LKHWLYKHRHNPYPTKTEKILLALGSHMTLVQ

Branchiostoma floridae
>MKX_Braflo
MVSSAPDGGKKWSRQEMCGTSGGIDHGGDRVYVNELRVSRKSPASVYLWVLQQTRGLRICGS
RPRGSVVRPETERCAGICVLPVVGERERVSAGWEKVHNKLENMVDCTTENELTIVSDSSSEF
SEDDLEYGGGGADGVLKVSSMTSKDLEERDRSLRRRSSRRRNSDRVRHKRQVMQDMARPLKQ
WLIKHRDNPYPTKTEKILLALTSQMTLVQVSNWFANARRRLKNTVRDPDLSWGMRIKLYNKH
VQGNAELLSISSGDDSDEDDFGDSDYGDHDGGSKGNLIIPHFKGDRDENSNNSSLPPSPTED
LDHRYAIPRYPDSGEEPSSPTKYKHSILQRYLNDSFHSTARRNDKSGSLSSQDYEDLSESAR
SFRSSFGSDGSVRLSPTSEGEPRSSPVFEDEELHWKEIDAAMALTSLARSRGCP*
