# Supplementary material for: Evolutionary history of the iroquois/Irx genes in metazoans
Source: BMC Evol Biol. 2009 Apr 15;9:74. doi: 10.1186/1471-2148-9-74 (PMC2674049; doi:10.1186/1471-2148-9-74)
Supplement: Additional file 2 — Multiple alignments of the conserved domains of Irx and Mkx proteins. These alignments only show the conserved domains of the Mkx and Irx proteins, as defined in [5]. Some of the newly-identified sequences are clearly incomplete, due to gap in the genome sequences and/or difficulties to predict compmete open reading frame from the genomic sequences. [file 1471-2148-9-74-S2.pdf]

## TALE-class Homeodomain

```
irx2_Aedaeg      SYREAYEYGDSNDWDRGSNNTARRKNATRESTATLKAWLNEHKKNPYPTKGEKIMLAIITKMTLTQVSTW
irx6_Galgal      --------------------GTRRKNATRETTSTLKTWLYEHRKNPYPTKGEKIMLAIITKMTLTQVSTW
irx-c_Petmar     -----------------VADPAFRKNATRDATATLKAWLNEHRKNPYPTKGEKIMLAIITRMTLTQVSTW
irx2_Schmed      DNVSNLIYKNIYVYPPDFPNAPRRKNATRETTSILKAWLNQHKKNPYPTKGEKIMLALITKMTLTQVSTW
irx3_Schmed      -----------------MNSHPRRKNATRETTSILKAWLNEHKKNPYPTKGEKIMLAVITKMTLTQVSTW
irx_Caeele       GPMPMFFSDAHRLYHPYGLDGIKKRNATREATAPLKDWLHSHRKNPYPSKADKVMLAVGTGMTLTQVSTW
irx1_Capsp1      WPYDPSNMTGYPYGASYGGLDVRRKNATRESTSTLKAWLYEHIKNPYPTKGEKIMLAIITKMTLTQVSTW
irx-b_Petmar     ----------------DSGGRLGSKAATRESTSTLKAWLSEHRKNPYPTKGEKIMLALVTKMTLTQVSTW
irx1_Schmed      DNSAWANPYGYIGNPTLDLNSARRKNATRESTSTLKAWLQEHIKNPYPTKGEKIMLAIITKMTLTQVSTW
irx1_Musmus      FAAHTTPAYYPYG-QFQYGDPGRPKNATRESTSTLKAWLNEHRKNPYPTKGEKIMLAIITKMTLTQVSTW
irx1_Homsap      FAAHTAPAYYPYG-QFQYGDPGRPKNATRESTSTLKAWLNEHRKNPYPTKGEKIMLAIITKMTLTQVSTW
irx1_Galgal      FAAHTAPGYYPYG-QFQYGDPGRPKNATRESTSTLKAWLNEHRKNPYPTKGEKIMLAIITKMTLTQVSTW
irx1_Xentro      FAAHTTPGYYPYG-QFQYGDPGRPKNATRESTSTLKAWLNEHRKNPYPTKGEKIMLAIITKMTLTQVSTW
irx1a_BRARER     FAAHTSPAFYPYG-QFQYGDPARPKNATRESTSTLKAWLNEHRKNPYPTKGEKIMLAIITKMTLTQVSTW
irx1b_BRARER     FAVHAAPGFYPYG-QYQYGDPARAKSATRETTSTLKAWLQEHKKNPYPTKGEKIMLAIITKMTLTQVSTW
CAF99496_Tetnig  LPVHAAQGFYPYG-QYPYGDPSRAKAATRETTSTLKAWLQEHQKNPYPTKGEKIMLAIITRMTLTQVSTW
irx3_Musmus      AFPHPHPAFYPYG-QYQFGDPSRPKNATRESTSTLKAWLNEHRKNPYPTKGEKIMLAIITKMTLTQVSTW
irx3_Homsap      AFPHPHPAFYPYG-QYQFGDPSRPKNATRESTSTLKAWLNEHRKNPYPTKGEKIMLAIITKMTLTQVSTW
irx3a_BRARER     -FAHHHPAFYPYG-QYQFGDPSRPKNATRESTSTLKAWLSEHRKNPYPTKGEKIMLAIITKMTLTQVSTW
CAG14759_Tetnig  -FAHHHPAFYPYG-QYQFGDPSRPKNATRESTSTLKAWLSEHRKNPYPTKGEKIMLAIITKMTLTQVSTW
irx3_Xentro      -FSHPHAAFYPYG-QYQFGDPSRPKNATRESTSTLKAWLNEHRKNPYPTKGEKIMLAIITKMTLTQVSTW
irx3b_BRARER     ---FPHAAFYPYGHQYQFGDPSRPKNATRESTSTLKAWLSEHRKNPYPTKGEKIMLAIITKMTLTQVSTW
irx-a_Petmar     -FPYPTAAYYSPYGGFQYTDPSRPKNATRESTATLKAWLNEHRKNPYPTKGEKIMLAIITKMTLTQVSTW
irx7_BRARER      ---RGGPYYTPYRPIPADDPSRVTKVATRESTSALKAWLSEHLKNPYPTKGEKIMLAIVTKMSLTQVSTW
CAG03859_Tetnig  ---RGAAFFPPYRHGAVEDPGRVAKVATRESTGALKAWLNEHLKNPYPTKGEKIMLAIITKMSLTQVSTW
irx2_Acypis      GYYHYDSTLAAYGYSAGYDLAARRKNATRESTATLKSWLNEHKKNPYPTKGEKIMLAIITKMTLTQVSTW
irx2_Pedhumcor   ------------XYSAGYDLAARRKNATRESTATLKAWLNEHKKNPYPTKGEKIMLAIITKMTLTQVSTW
caup_Dromel      GYYSYDPTLAAYGYGPNYDLAARRKNATRESTATLKAWLSEHKKNPYPTKGEKIMLAIITKMTLTQVSTW
irx_Calvic       GYYSYDPTFAYG-YGASYDLASRRKNATRESTATLKAWLNEHKKNPYPTKGEKIMLAIITKMTLTQVSTW
ara_Dromel       GYYSYDPMSAYGGYGASYDLAARRKNATRESTATLKAWLNEHKKNPYPTKGEKIMLAIITKMTLTQVSTW
irx2_Tricas      GYYPYDPALAAYGYGAGYDLAARRKNATRESTATLKAWLNEHKKNPYPTKGEKIMLAIITKMTLTQVSTW
irx2_Culpipqui   ----------QTTTPATKSETARRKNATRESTATLKAWLNEHKKNPYPTKGEKIMLAIITKMTLTQVSTW
irx2_Helera      GYYPYDPTLAAYGYGAGYDLAARRKNATTESTATLKAWLNEHKKNPYPTKGEKIMLAIITKMTLTQVSTW
irx_Spofru       GYYPYDPTLAAYGYGAGYDLAARRKNATRESTATLKAWLNEHKKNPYPTKGEKIMLAIITKMTLTQVSTW
irx2_Bommor      GYYPYDPTLAAYGYGAGYDLAARRKNATRESTATLKAWLNEHKKNPYPTKGEKIMLAIITKMTLTQVSTW
irx2_Anogam      GYYPYDPTLAAYGYGAGYDLAARRKNATRESTATLKAWLNEHKKNPYPTKGEKIMLAIITKMTLTQVSTW
irx2_Nasvit      GYYPYDPTLAAYGYGAGYDLAARRKNATRESTATLKAWLNEHKKNPYPTKGEKIMLAIITKMTLTQVSTW
irx2_Apimel      GYYPYDPTLAAYGYGAGYDLAARRKNATRESTATLKAWLNEHKKNPYPTKGEKIMLAIITKMTLTQVSTW
irx2_Dappul      GYYPYDPALAAYGYGASYDLAARRKNATRESTATLKAWLSEHKKNPYPTKGEKIMLAIITKMTLTQVSTW
irx3_Capsp1      YFPAYELAGFPYHNGFALDPNARRKNATRESTTTLKAWLNEHRKNPYPTKGEKIMLAIITKMTLTQVSTW
irx2_Capsp1      DIHGSYQYLSQAYASLDP----RRKNATKESTNTLKAWLYEHKKNPYPTKGEKIMLAILTRMTLTQVSTW
irx1_Lotgig      DTPMSSFPYSNAYGSVDLNSATRRKNATRENTTTLKAWLYEHKKNPYPTKGEKIMLAIITKMTLTQVSTW
irx1_Aedaeg      HPYDAAFG-YPFNSYGMDLNGARRKNATRETTSTLKAWLNEHKKNPYPTKGEKIMLAIITKMTLTQVSTW
irx1_Culpipqui   -------------SYGMDLNGARRKNATRETTSTLKAWLNEHKKNPYPTKGEKIMLAIITKMTLTQVSTW
irx1_Anogam      HPYDAAFG-YPFNSYGMDLNGARRKNATRETTSTLKAWLNEHKKNPYPTKGEKIMLAIITKMTLTQVSTW
irx1_Bommor      ----------------MDLNGARRKNATRETTSTLKAWLNEHKKNPYPTKGEKIMLAIITKMTLTQVSTW
irx1_Apimel      HPYDAAFASYPFNGYGMDLNGARRKNATRETTSTLKAWLNDHKKNPYPTKGEKIMLAIITKMTLTQVSTW
irx1_Nasvit      HPYDAAFASYPFNGYGMDLNGARRKNATRETTSTLKAWLNEHKKNPYPTKGEKIMLAIITKMTLTQVSTW
irx1_Helera      HPYDAAFAGYPFNGYGMDLNGARRKNATRETTSTLKAWLNEHKKNPYPTKGEKIMLAIITKMTLTQVSTW
irx1_Tricas      HPYDTAFAGYPFNGYGMDLNGARRKNATRETTSTLKAWLNEHKKNPYPTKGEKIMLAIITKMTLTQVSTW
irx1_Acypis      HPYDAAFG-YPFNGYGMDLNGARRKNATRETTSTLKAWLNEHKKNPYPTKGEKIMLAIITKMTLTQVSTW
irx1_Pedhumcor   ----------------MDLNGARRKNATRETTSTLKAWLNEHKKNPYPTKGEKIMLAIITKMTLTQVSTW
mirr_Dromel      HPYDAAFAGYPFNSYGMDLNGARRKNATRETTSTLKAWLNEHKKNPYPTKGEKIMLAIITKMTLTQVSTW
irx1_Dappul      YPYDPAMAAYPFGGYGMDLNGSRRKNATRETTATLKAWLSEHKKNPYPTKGEKIMLAIITKMTLTQVSTW
irx4_Lotgig      HPYNTFYAGLDLNSA------ARRKAATRETTSPLKAWLSEHRKNPYPTKAEKIMLAIITKMTLTQVSTW
irx_Mytcal       HPYASFYGGLDLNNA------ARRKNATRETTSALKAWLYEHRKNPYPTKGEKIMLAIITKMTLTQVSTW
irx2_Lotgig      YPYGPGYGGLDLN--------ARRKNATRESTTTLKAWLNEHIKNPYPTKGEKIMLAIITKMTLTQVSTW
irx3_Lotgig      YPYGAGFGGLDLN--------SRRKNATRENTTTLKAWLYEHRKNPYPTKGEKIMLAIITKMTLTQVSTW
irx_Sackov       -YDPAYQYYGDRYGGVDINGAARRKNATRETTSTLKAWLYEHRKNPYPTKGEKIMLAIITKMTLTQVSTW
irx_Strpur       YDPATYQYYGDRYG-MDLN-GARRKNATRETTSTLKAWLYEHRKNPYPTKGEKIMLAIITKMTLTQVSTW
irx4_Musmus      YEPALSQYPYDRYG--TVDSGTRRKNATRETTSTLKAWLQEHRKNPYPTKGEKIMLAIITKMTLTQVSTW
irx4_Homsap      YEPALGQYPYDRYG--TMDSGTRRKNATRETTSTLKAWLQEHRKNPYPTKGEKIMLAIITKMTLTQVSTW
irx4_Galgal      YEHSLSQYQYDRYG--AMDGGTRRKNATRETTSTLKAWLQEHRKNPYPTKGEKIMLAIITKMTLTQVSTW
irx4_Xentro      YEHTLSQYQYDRYG--TMDGSTRRKNATRETTSTLKAWLQEHRKNPYPTKGEKIMLAIITKMTLTQVSTW
irx4a_BRARER     YDPTLGQYQYDRYG--SMEGGTRRKNATRETTSTLKAWLQEHRKNPYPTKGEKIMLAIITKMTLTQVSTW
irx4a_Tetnig     YDPTLGQYQYDRYG--SMDGGTRRKNATRETTSTLKAWLQEHRKNPYPTKGEKIMLAIITKMTLTQVSTW
irx4b_BRARER     YDYPIGQYSYDRYGYGSVDVGTRRKNATRETTSTLKAWLQEHKKNPYPTKGEKIMLAIITKMTLTQVSTW
irx4b_Tetnig     YEYPFGQYPYERYSYSCADGASRRKNATRETTSTLKAWLQEHQKNPYPTKGEKIMLAIITRMTLTQVSTW
irx6_Musmus      YETTLGQYQYDRYGGVELSSAGRRKNATRESTSALKAWLHEHRKNPYPTKGEKIMLAIITKMTLTQVSTW
irx6_Homsap      YERTLGQYQYERYGAVELSGAGRRKNATRETTSTLKAWLNEHRKNPYPTKGEKIMLAIITKMTLTQVSTW
irx6a_BRARER     YDHSLGQYQYDRYGTVDFNGSTRRKNATRETTSTLKTWLYEHRKNPYPTKGEKIMLAIITKMTLTQVSTW
Irx6a_Tetnig     YDHSLGQYQYDRYGTVDFNSTARRKNATRETTSTLKTWLYEHRKNPYPTKGEKIMLAIITKMTLTQVSTW
irx6_Xentro      YEHSLGQYQYDRFGAVDISCSSRRKNATRENTSTLKTWLYEHRKNPYPTKGEKIMLAIITKMTLTQVSTW
irxA_Braflo      -----AGGAPYPYGSYAAMDGARRKNATRETTATLKAWLMEHRKNPYPTKGEKIMLAIITKMTLTQVSTW
irxB_Braflo      ---LAAGAQYHPYGGYGPMDGTPRKNATRDATSTLKAWLNEHKKNPYPTKGEKIMLAIITKMTLTQVSTW
irx-d_Petmar     --------------------VTRRKNATRDATATLKAWLNEHRKNPYPTKGEKIMLAIITRMTLTQISTW
irx2_Musmus      -----------AAYPYQLNDPAYRKNATRDATATLKAWLNEHRKNPYPTKGEKIMLAIITKMTLTQVSTW
irx2_Homsap      -----------AAYPYQLNDPAYRKNATRDATATLKAWLNEHRKNPYPTKGEKIMLAIITKMTLTQVSTW
irx2_Galgal      -----------PAYPYQLNDPAYRKNATRDATATLKAWLQEHRKNPYPTKGEKIMLAIITKMTLTQVSTW
irx2_Xentro      -----------AAYPYQLNDPAYRKNATRDATATLKAWLQEHRKNPYPTKGEKIMLAIITKMTLTQVSTW
irx2a_BRARER     -----------PGYPYQLNDPAYRKNATRDATATLKAWLQEHRKNPYPTKGEKIMLAIITKMTLTQVSTW
irx2_Tetnig      -----------PGYPYQLNDPAYRKNATRDATATLKAWLQEHRKNPYPTKGEKIMLAIITKMTLTQVSTW
irx5_Musmus      -----------PLGSYPYGDPAYRKNATRDATATLKAWLNEHRKNPYPTKGEKIMLAIITKMTLTQVSTW
irx5_Homsap      -----------PLGSYPYGDPAYRKNATRDATATLKAWLNEHRKNPYPTKGEKIMLAIITKMTLTQVSTW
irx5_Galgal      -----------PLGSYPYGDPAYRKNATRDATATLKAWLNEHRKNPYPSKAP------------------
irx5_Xentro      -----------PLGTYPYGDPAYRKNATRDATATLKAWLNEHRKNPYPTKGEKIMLAIITKMTLTQVSTW
irx5a_BRARER     -----------PLGSYPYGDPAYRKNATRDATATLKAWLNEHRKNPYPTKGEKIMLAIITKMTLTQVSTW
irx5a_Tetnig     -----------PLGTYPYGDPAYRKNATRDATATLKAWLNEHRKNPYPTKGEKIMLAIITKMTLTQVSTW
irx5b_BRARER     -----------PLGPYPFGDPAYRKNATRDATATLKAWLNEHRKNPYPTKGEKIMLAIITKMTLTQVSTW
irx5b_Tetnig     -----------PLGPYPFGDPTYRKNATRDATATLKAWLSEHRKNPYPTKGEKIMLAIITKMTLTQVSTW
irxNemvec        THPACTRFAERFSPIDLAAAGARRKNATRETTSTLKAWLFEHRKNPYPTKGEKIMLAILTKMTLTQVSTW
irx_Triadh       YPVGHVDGISRFNPIDLN--NARRKNATRETTSILKAWLYEHRKNPYPTKGEKIMLAIMTKMTLTQVSTW
irx4_Schmed      NLNPITLAHLHTINHADVNQIQVKKNATRENSSTLKAWLQEHKDNPYPSKGEKILLAILTQMSLTQVSTW
irx_Hydmag       --YNKYNNESCSILDYALLASARRKNATKETTSVLKSWLNEHRKNPYPSKSEKVMLAIMTKMTLTQVSTW
irxa_Ampque      ----LATGGSGSSGSLSGYSAAGSITRRMRNTAVLVKWIEDHQSNPYPTKAEKQYLAYYSGMNMTQLSTW
irxe_Ampque      -----------------------------RNTYVLEKWMQEHMTRLYPTAEEKQCLAHFSRMNTKQNIAW
irx-a_Subdom     ----HTGRAVVNGQSPSPQRSTCSGPRKPRNPAVLVLWIEEHSANPYPTKAEKNFLAHYAGMTTRQLNDW
irxc_Ampque      -----QGSHSVADGSKASQEKGSSSPGSWRNTDVLALWITEHLQLPYPGKVEKQYLCFYSNMSMKQVSTY
irxd_Ampque      -------------------EKGSSSPGSWRNTDVFALWITEHQQFPYPSKVEKQYLCYYSNMSMKQVSTY
irxb_Ampque      -----------SMEGLEGSVQLASSRRRRRDATHLIEWLDLHQGNPYPTRVEKEQLVVISGMNFKQLNDW
```

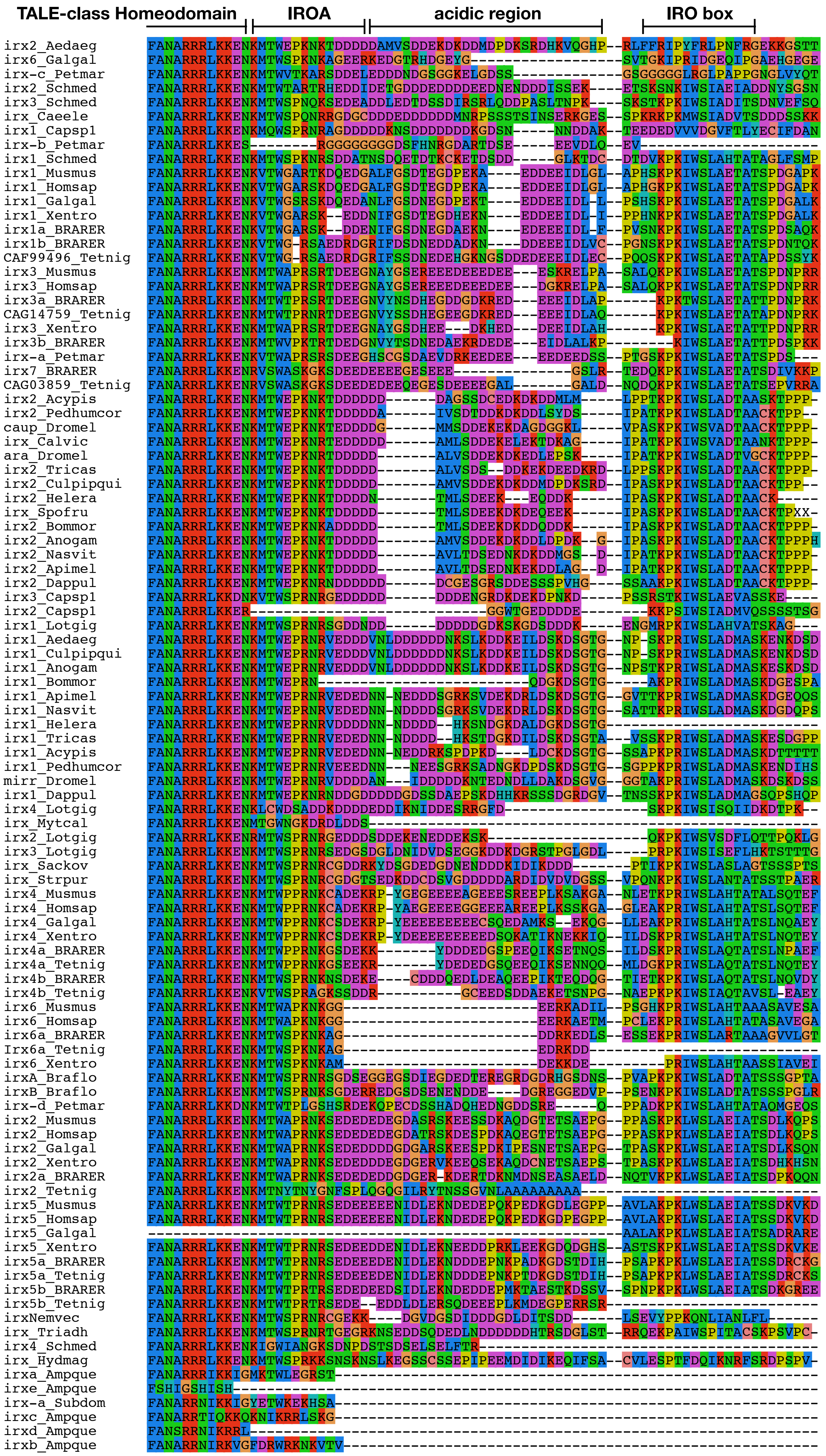

TALE-class Homeodomain
IROA
acidic region
IRO box
irx2_Aedaeg
irx6_Galgal
irx-c_Petmar
irx2_Schmed
irx3_Schmed
irx_Caeele
irx1_Capsp1
irx-b_Petmar
irx1_Schmed
irx1_Musmus
irx1_Homsap
irx1_Galgal
irx1_Xentro
irx1a_BRARER
irx1b_BRARER
CAF99496_Tetnig
irx3_Musmus
irx3_Homsap
irx3a_BRARER
CAG14759_Tetnig
irx3_Xentro
irx3b_BRARER
irx-a_Petmar
irx7_BRARER
CAG03859_Tetnig
irx2_Acypis
irx2_Pedhumcor
caup_Dromel
irx_Calvic
ara_Dromel
irx2_Tricas
irx2_Culpipqui
irx2_Helera
irx_Spofru
irx2_Bommor
irx2_Anogam
irx2_Nasvit
irx2_Apimel
irx2_Dappul
irx3_Capsp1
irx2_Capsp1
irx1_Lotgig
irx1_Aedaeg
irx1_Culpipqui
irx1_Anogam
irx1_Bommor
irx1_Apimel
irx1_Nasvit
irx1_Helera
irx1_Tricas
irx1_Acypis
irx1_Pedhumcor
mirr_Dromel
irx1_Dappul
irx4_Lotgig
irx_Mytcal
irx2_Lotgig
irx3_Lotgig
irx_Sackov
irx_Strpur
irx4_Musmus
irx4_Homsap
irx4_Galgal
irx4_Xentro
irx4a_BRARER
irx4a_Tetnig
irx4b_BRARER
irx4b_Tetnig
irx6_Musmus
irx6_Homsap
irx6a_BRARER
Irx6a_Tetnig
irx6_Xentro
irxA_Braflo
irxB_Braflo
irx-d_Petmar
irx2_Musmus
irx2_Homsap
irx2_Galgal
irx2_Xentro
irx2a_BRARER
irx2_Tetnig
irx5_Musmus
irx5_Homsap
irx5_Galgal
irx5_Xentro
irx5a_BRARER
irx5a_Tetnig
irx5b_BRARER
irx5b_Tetnig
irxNemvec
irx_Triadh
irx4_Schmed
irx_Hydmag
irxa_Ampque
irxe_Ampque
irx-a_Subdom
irxc_Ampque
irxd_Ampque
irxb_Ampque
