# Supplementary figures and images for: Evolutionary history of the iroquois/Irx genes in metazoans
Source: BMC Evol Biol. 2009 Apr 15;9:74. doi: 10.1186/1471-2148-9-74 (PMC2674049; doi:10.1186/1471-2148-9-74)

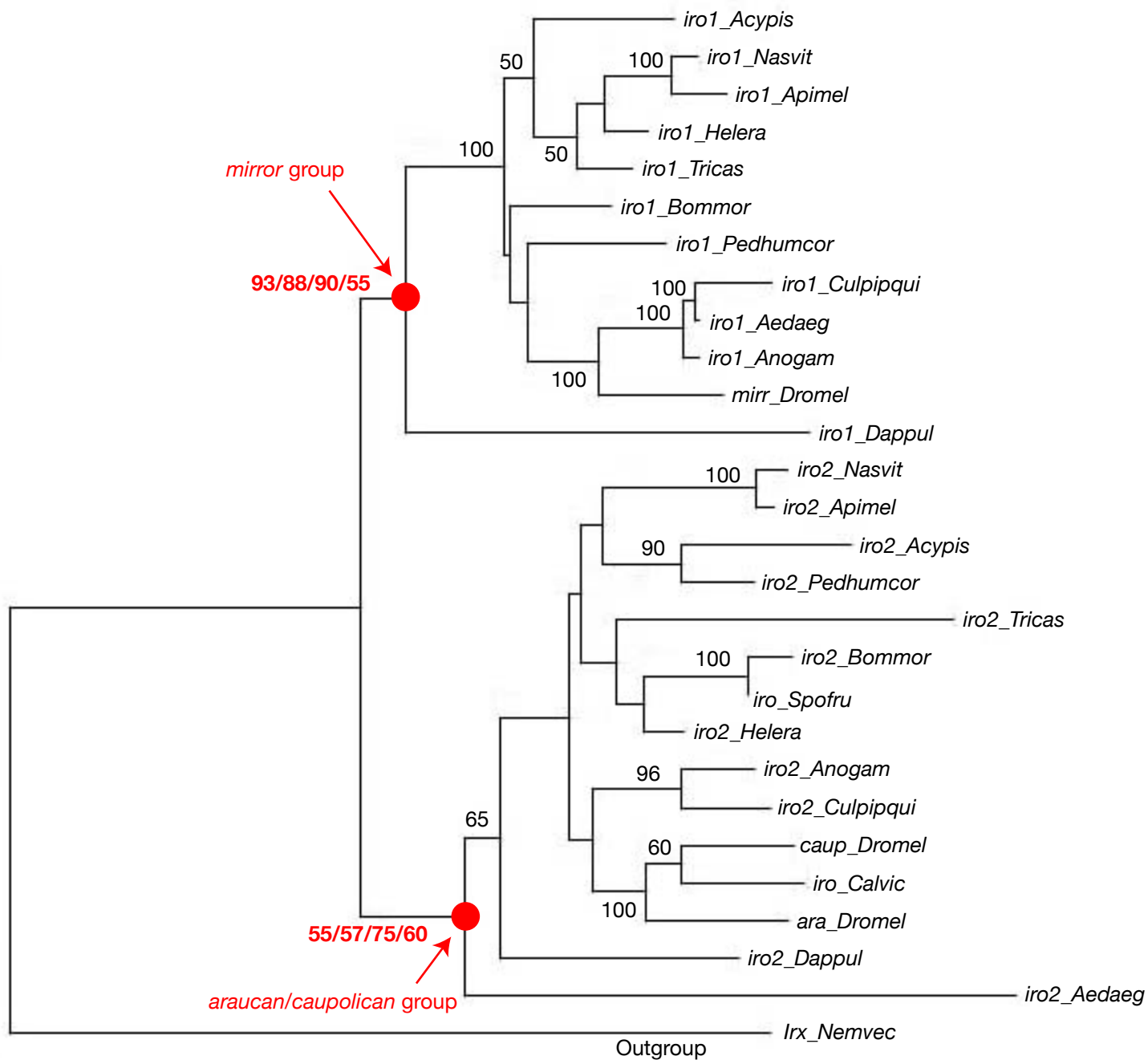

Supplement: Additional file 3 — Phylogenetic analysis of Irx genes in arthropods. The represented tree is a maximum-likelihood tree, based on an alignment of the full-lenght protein sequences and which has been rooted using the Irx gene from Nematostella as outgroup (this should be considered as arbitrary rooting). The most important monophyletic groups are indicated in red and the associated numbers are their statistical support values obtained with different methods of phylogenetic reconstruction, as described in Figure 2. Statistical support in the maximum-likelihood analysis for some other internal branches is indicated in black. [file 1471-2148-9-74-S3.pdf]
