# Supplementary material for: Evolutionary history of the iroquois/Irx genes in metazoans
Source: BMC Evol Biol. 2009 Apr 15;9:74. doi: 10.1186/1471-2148-9-74 (PMC2674049; doi:10.1186/1471-2148-9-74)
Supplement: Additional file 4 — Summary of the study of the genomic organization of the Irx genes and their physical association with CG10632 genes. This table contains the genomic data that have allowed to construct Figure 6. [file 1471-2148-9-74-S4.pdf]

Drosophila melanogaster	***CG4069***							Size = 206144 bp
Chromosome 3L	5' position	3' position	Gene size (nt)	CDS Size (nt)	protein Size (aa)	Orientation	Total number of exons	
	12,528,524	12,530,410	1706	1530	509	Reverse	4	
	Gap : 199 bp							
	CG10632							
	5' position	3' position	Gene size (nt)	CDS Size (nt)	protein Size (aa)	Orientation	Total number of exons	
	12,530,609	12,565,622	35014	1884	627	Reverse	8	
	Gap : 8152 bp							
	araucan							
	5' position	3' position	Gene size (nt)	CDS Size (nt)	protein Size (aa)	Orientation	Total number of exons	
	12,573,774	12,589,118	15345	2154	717	Forward	5	
	Gap : 14108 bp							
	caupolican							
	5' position	3' position	Gene size (nt)	CDS Size (nt)	protein Size (aa)	Orientation	Total number of exons	
	12,603,226	12,614,908	11683	2082	693	Forward	5	
	Gap : 6128 bp							
	CG32111							
	5' position	3' position	Gene size (nt)	CDS Size (nt)	protein Size (aa)	Orientation	Total number of exons	
	12,621,036	12,641,380	20345	513	170	Forward	7	
	Gap : 45468 bp							
	mirror							
	5' position	3' position	Gene size (nt)	CDS Size (nt)	protein Size (aa)	Orientation	Total number of exons	
	12,686,848	12,703,243	16396	1926	641	Forward	5	
	Gap : 24806 bp							
	CG10975-PTP69D							
	5' position	3' position	Gene size (nt)	CDS Size (nt)	protein Size (aa)	Orientation	Total number of exons	
	12,728,049	12,734,668	6620	4389	1462	Forward	12	

Anopheles gambiae	***CG4069***							Size = 472591 bp
Chromosome 2L	5' position	3' position	Gene size (nt)	CDS Size (nt)	protein Size (aa)	Orientation	Total number of exons	
	7,816,277	7,818,115	1838	1672	533	Reverse	3	
	Gap : 2615 bp							
	CG10632							
	5' position	3' position	Gene size (nt)	CDS Size (nt)	protein Size (aa)	Orientation	Total number of exons	
	7,820,730	7,916,840	96111	1965	654	Reverse	10	
	Gap : 56641 bp							
	araucan/caupolican							
	5' position	3' position	Gene size (nt)	CDS Size (nt)	protein Size (aa)	Orientation	Total number of exons	
	7,973,481	8,020,949	47469	2349	782	Forward	6	
	Gap : 57339 bp							
	mirror							
	5' position	3' position	Gene size (nt)	CDS Size (nt)	protein Size (aa)	Orientation	Total number of exons	
	8,078,288	8,136,322	58035	1728	575	Forward	5	
	Gap : 150350 bp							
	CG5288							
	5' position	3' position	Gene size (nt)	CDS Size (nt)	protein Size (aa)	Orientation	Total number of exons	
	8,286,672	8,288,868	2197	1769	477	Forward	7	

Nasonia vitripennis	***similar to Juvenile hormone-inducible protein***							Size = 422674 bp
Chromosome Unknown	5' position	3' position	Gene size (nt)	CDS Size (nt)	protein Size (aa)	Orientation	Total number of exons	
Scaffold 12	2,251,036	2,301,061	50026	1197	398	Reverse	3	
	Gap : 114367 bp							
	mirror							
	5' position	3' position	Gene size (nt)	CDS Size (nt)	protein Size (aa)	Orientation	Total number of exons	
	2,415,428	2,442,094	26666	1953	650	Reverse	5	
	Gap : 54098 bp							
	araucan/caupolican							
	5' position	3' position	Gene size (nt)	CDS Size (nt)	protein Size (aa)	Orientation	Total number of exons	
	2,496,192	2,552,257	56066	2112	703	Reverse	7	
	Gap : 8427 bp							
	CG10632							
	5' position	3' position	Gene size (nt)	CDS Size (nt)	protein Size (aa)	Orientation	Total number of exons	
	2,560,684	2,669,901	109218	1782	594	Forward	11	
	Gap : 2766 bp							
	similar to mCG64768							
	5' position	3' position	Gene size (nt)	CDS Size (nt)	protein Size (aa)	Orientation	Total number of exons	
	2,672,667	2,673,710	1044	1044	347	Forward	1	

Tribolium castaneum	***CG31638***							

Linkage Group 3	5' position	3' position	Gene size (nt)	CDS Size (nt)	protein Size (aa)	Orientation	Total number of exons	Size = 442853 bp
Scaffold 12	1,629,351	1,636,576	7226	1557	518	Forward	6	
	Gap : 1051 bp							
	CG10632							
	5' position	3' position	Gene size (nt)	CDS Size (nt)	protein Size (aa)	Orientation	Total number of exons	
	1,637,627	1,718,132	80506	1395	464	Reverse	9	
	Gap : 322 bp							
	araucan/caupolican							
	5' position	3' position	Gene size (nt)	CDS Size (nt)	protein Size (aa)	Orientation	Total number of exons	
	1,718,454	1,845,814	127361	1939	710	Forward	9	
	Gap : 135 bp							
	mirror							
	5' position	3' position	Gene size (nt)	CDS Size (nt)	protein Size (aa)	Orientation	Total number of exons	
	1,845,949	1,879,353	33405	1377	458	Forward	5	
	Gap : 176682 bp							
	CG5288							
	5' position	3' position	Gene size (nt)	CDS Size (nt)	protein Size (aa)	Orientation	Total number of exons	
	2,056,035	2,072,204	16170	1044	459	Forward	7	
Daphnia pulex	***GNO_270333***							Size = 125347 bp
Scaffold 33	5' position	3' position	Gene size (nt)	CDS Size (nt)	protein Size (aa)	Orientation	Total number of exons	
	769,040	770,090	1855	1046	288	Reverse	4	
	Gap : 55246 bp							
	mirror							
	5' position	3' position	Gene size (nt)	CDS Size (nt)	protein Size (aa)	Orientation	Total number of exons	
	825,336	836,158	8150	2775	925	Reverse	5	
	Gap : 25759 bp							
	araucan/caupolican							
	5' position	3' position	Gene size (nt)	CDS Size (nt)	protein Size (aa)	Orientation	Total number of exons	
	861,917	871,863	9946	1939	784	Reverse	9	
	Gap : 4856 bp							
	CG10632							
	5' position	3' position	Gene size (nt)	CDS Size (nt)	protein Size (aa)	Orientation	Total number of exons	
	876,719	893,462	16744	1713	570	Forward	11	
	Gap : 489 bp							
	Pumpless							
	5' position	3' position	Gene size (nt)	CDS Size (nt)	protein Size (aa)	Orientation	Total number of exons	
	893,951	894,387	437	366	123	Reverse	2	
Pediculus humanus corporis	***Dynein, axonemal, heavy chain 5 [CG9492]***							Size = 268402 bp
Supercontig 1103172108298	5' position	3' position	Gene size (nt)	CDS Size (nt)	protein Size (aa)	Orientation	Total number of exons	
	166,574	184,606	18032	10980	3660	Reverse	39	
	Gap : 5376 bp							
	CG10632							
	5' position	3' position	Gene size (nt)	CDS Size (nt)	protein Size (aa)	Orientation	Total number of exons	
	189,982	233,138	43210	1713	500	Reverse	8	
	Gap : 4207 bp							
	araucan/caupolican							
	5' position	3' position	Gene size (nt)	CDS Size (nt)	protein Size (aa)	Orientation	Total number of exons	
	237,345	286,802	49458			Forward		
	Gap : 51009 bp							
	mirror							
	5' position	3' position	Gene size (nt)	CDS Size (nt)	protein Size (aa)	Orientation	Total number of exons	
	337,811	355,532	17721	1575	525	Forward	5	
	Gap : 79073 bp							
	Thioredoxin like protein							
	5' position	3' position	Gene size (nt)	CDS Size (nt)	protein Size (aa)	Orientation	Total number of exons	
	434,605	434,976	372	372	124	Forward	1	
Caenorhabditis elegans	***C41G7.7***							Size = 40797 bp
Chromosome I	5' position	3' position	Gene size (nt)	CDS Size (nt)	protein Size (aa)	Orientation	Total number of exons	
	9,533,571	9,536,262	2692	471	156	Reverse	6	
	Gap : 194 bp							
	Irx (C36F7.1)							
	5' position	3' position	Gene size (nt)	CDS Size (nt)	protein Size (aa)	Orientation	Total number of exons	
	9,536,456	9,540,959	4504	1134	377	Reverse	5	
	Gap : 3780 bp							
	CG10632 (C36F7.2)							
	5' position	3' position	Gene size (nt)	CDS Size (nt)	protein Size (aa)	Orientation	Total number of exons	
	9,544,739	9,558,087	13349	897	298	Forward	8	

	Gap : 7298 bp							
	similar to CG32791 (C36F7.4b)							
	5' position	3' position	Gene size (nt)	CDS Size (nt)	protein Size (aa)	Orientation	Total number of exons	
	9,565,385	9,574,368	8983	843	280	Reverse	10	
Trichoplax adhaerens	***Myosin3 MHCA***							Size = 69611 bp
Scaffold 4	5' position	3' position	Gene size (nt)	CDS Size (nt)	protein Size (aa)	Orientation	Total number of exons	
	5,176,906	5,187,217	10312	4344	1448	Forward		
	Gap : 7933 bp							
	Carbonic anhydrase related protein VIII							
	5' position	3' position	Gene size (nt)	CDS Size (nt)	protein Size (aa)	Orientation	Total number of exons	
	5,195,150	5,198,703	3553			Forward		
	Gap : 28043 bp							
	Irx							
	5' position	3' position	Gene size (nt)	CDS Size (nt)	protein Size (aa)	Orientation	Total number of exons	
	5,226,746	5,228,948	2202			Forward		
	Gap : 2934 bp							
	Perocisomal LON							
	5' position	3' position	Gene size (nt)	CDS Size (nt)	protein Size (aa)	Orientation	Total number of exons	
	5,231,882	5,246,517	14635			Forward		
Capitella spI	***phosphorylated CTD interacting factor 1***							Size = 98983 bp
Scaffold 402	5' position	3' position	Gene size (nt)	CDS Size (nt)	protein Size (aa)	Orientation	Total number of exons	
	81,537	84,783	3247	1587	1950	Reverse	10	
	Gap : 9009bp							
	Irx-3							
	5' position	3' position	Gene size (nt)	CDS Size (nt)	protein Size (aa)	Orientation	Total number of exons	
	94,772	102,257	7491	1434	477	Reverse	6	
	Gap : 6096 bp							
	CG10632							
	5' position	3' position	Gene size (nt)	CDS Size (nt)	protein Size (aa)	Orientation	Total number of exons	
	108,353	145,607	37255	1587	527	Forward	11	
	Gap : 9020 bp							
	Irx-1							
	5' position	3' position	Gene size (nt)	CDS Size (nt)	protein Size (aa)	Orientation	Total number of exons	
	154627	159328	4701			Reverse		
	Gap : 9425 bp							
	Irx-2							
	5' position	3' position	Gene size (nt)	CDS Size (nt)	protein Size (aa)	Orientation	Total number of exons	
	168,753	172,225	3472	1080	360	Forward	5	
	Gap : 4897 bp							
	coiled-coil domain containing 90A							
	5' position	3' position	Gene size (nt)	CDS Size (nt)	protein Size (aa)	Orientation	Total number of exons	
	177,122	180,520	3398	780	260	Reverse	7	
Lottia gigantea	***CG10632***							
Scaffold 838	5' position	3' position	Gene size (nt)	CDS Size (nt)	protein Size (aa)	Orientation	Total number of exons	
	1,547	11,895	10348	1425	474	Forward	12	
Lottia gigantea	***Fantom***							Size = 578630 bp
Scaffold 9	5' position	3' position	Gene size (nt)	CDS Size (nt)	protein Size (aa)	Orientation	Total number of exons	
	1,194,379	1,212,782	18404	4032	1344	Reverse	35	
	Gap : 29570 bp							
	Irx-4							
	5' position	3' position	Gene size (nt)	CDS Size (nt)	protein Size (aa)	Orientation	Total number of exons	
	1,242,352	1,247,808	5457	1299	433	Forward	5	
	Gap : 47575 bp							
	Irx-2							
	5' position	3' position	Gene size (nt)	CDS Size (nt)	protein Size (aa)	Orientation	Total number of exons	
	1,295,383	1,303,744	8362	1293	430	Forward	6	
	Gap : 34139 bp							
	Irx-1							
	5' position	3' position	Gene size (nt)	CDS Size (nt)	protein Size (aa)	Orientation	Total number of exons	
	1,337,883	1,349,426	11544	1386	461	Reverse	5	
	Gap : 304880 bp							
	Irx-3							
	5' position	3' position	Gene size (nt)	CDS Size (nt)	protein Size (aa)	Orientation	Total number of exons	
	1,654,306	1,669,438	15133	1458	485	Reverse	6	
	Gap : 99740bp							
	Serotonin Receptor							

	5' position	3' position	Gene size (nt)	CDS Size (nt)	protein Size (aa)	Orientation	Total number of exons	
	1,769,178	1,773,009	3832	933	311	Forward	2	

Species	5' position	3' position	Gene size (nt)	CDS Size (nt)	protein Size (aa)	Orientation	Total number of exons	Size
Branchiostoma floridae<br>Scaffold 90 ou 632	***Myosin3 MHCA***							Size = 274216 bp
	5' position	3' position	Gene size (nt)	CDS Size (nt)	protein Size (aa)	Orientation	Total number of exons	
	1,661,617	1,679,697	18081	3669	1223	Reverse		
	Gap : 19281 bp							
	CG10632							
	5' position	3' position	Gene size (nt)	CDS Size (nt)	protein Size (aa)	Orientation	Total number of exons	
	1,698,978	1,753,608	54630	1509	502	Reverse	11	
	Gap : 14900 bp							
	Irx-C							
	5' position	3' position	Gene size (nt)	CDS Size (nt)	protein Size (aa)	Orientation	Total number of exons	
	1,768,508	1,772,293	3786	1461	486	Forward	7	
	Gap : 79295 bp							
	Irx-A							
	5' position	3' position	Gene size (nt)	CDS Size (nt)	protein Size (aa)	Orientation	Total number of exons	
	1,851,588	1,854,615	3028	1488	495	Forward	7	
	Gap : 36719 bp							
	Irx-B							
	5' position	3' position	Gene size (nt)	CDS Size (nt)	protein Size (aa)	Orientation	Total number of exons	
	1,891,334	1,895,774	4441	1485	494	Reverse	7	
	Gap : 32745 bp							
	Carbonic anhydrase related protein VIII							
	5' position	3' position	Gene size (nt)	CDS Size (nt)	protein Size (aa)	Orientation	Total number of exons	
	1,928,519	1,935,833	7314	846	282	Reverse	7	

Species	5' position	3' position	Gene size (nt)	CDS Size (nt)	protein Size (aa)	Orientation	Total number of exons	Size
Homo Sapiens<br>cluster IrxB<br>Chromosome 16	***FTO***							Size = 1,802,728 bp
	5' position	3' position	Gene size (nt)	CDS Size (nt)	protein Size (aa)	Orientation	Total number of exons	
	52,295,376	52,705,882	410507	1518	505	Forward	9	
	Gap : 168831 bp							
	Irx-3							
	5' position	3' position	Gene size (nt)	CDS Size (nt)	protein Size (aa)	Orientation	Total number of exons	
	52,874,713	52,877,879	3167	1506	501	Reverse	4	
	Gap : 644733 bp							
	Irx-5							
	5' position	3' position	Gene size (nt)	CDS Size (nt)	protein Size (aa)	Orientation	Total number of exons	
	53,522,612	53,525,898	3287	1452	483	Forward	3	
	Gap : 390074 bp							
	Irx-6							
	5' position	3' position	Gene size (nt)	CDS Size (nt)	protein Size (aa)	Orientation	Total number of exons	
	53,915,972	53,922,173	6202	1341	446	Forward	6	
	Gap : 148416 bp							
	MMP2 matrix metallopeptidase 2							
	5' position	3' position	Gene size (nt)	CDS Size (nt)	protein Size (aa)	Orientation	Total number of exons	
	54,070,589	54,098,104	27516	1983	660	Forward	12	

Species	5' position	3' position	Gene size (nt)	CDS Size (nt)	protein Size (aa)	Orientation	Total number of exons	Size
Homo Sapiens<br>cluster IrxA<br>Chromosome 5	***NDUFS6 NADH dehydrogenase***							Size = 3,518,903 bp
	5' position	3' position	Gene size (nt)	CDS Size (nt)	protein Size (aa)	Orientation	Total number of exons	
	1,854,509	1,869,165	14657	375	124	Forward	4	
	Gap : 61384bp							
	Irx-4							
	5' position	3' position	Gene size (nt)	CDS Size (nt)	protein Size (aa)	Orientation	Total number of exons	
	1,930,549	1,935,880	5332	1560	519	Reverse	5	
	Gap : 864000bp							
	Irx-2							
	5' position	3' position	Gene size (nt)	CDS Size (nt)	protein Size (aa)	Orientation	Total number of exons	
	2,799,880	2,804,769	4890	1416	471	Reverse	4	
	Gap : 844399bp							
	Irx-1							
	5' position	3' position	Gene size (nt)	CDS Size (nt)	protein Size (aa)	Orientation	Total number of exons	
	3,649,168	3,654,517	5350	1443	480	Forward	4	
	Gap : 644733 bp							
	ADAMTS16 ADAM metallopeptidase with thrombospondin type 1 motif, 16							
	5' position	3' position	Gene size (nt)	CDS Size (nt)	protein Size (aa)	Orientation	Total number of exons	
	5,193,443	5,373,412	179970	1443	480	Forward	4	
