# Supplementary material for: Evolutionary history of the iroquois/Irx genes in metazoans
Source: BMC Evol Biol. 2009 Apr 15;9:74. doi: 10.1186/1471-2148-9-74 (PMC2674049; doi:10.1186/1471-2148-9-74)
Supplement: Additional file 5 — Multiple alignments of sequences of the CG10632 proteins. This file contains the alignment of the whole sequence of the CG10632 proteins. [file 1471-2148-9-74-S5.pdf]

```
Nemvec-CG10632  -MSANEPLTLALVREFILKNGSRVRNHDLITHFRTQLNDPVNKGKVRADFKEYVHQLTTVQTID-GEKYFVLKKPKEAPAVLRKPPTRPHTTHHK---
Dappul-CG10632  -MAAPTEFTLESVRAFMLERGGKVTNHELVKHFKQFLTDPYTK-VARVQFKEYVNTLALIRQEE-SDKFLVLKPIFYPTEYLATDRVRDVDPSYHSVG
Tricas-CG10632  --MASSDLTLEQIYNYFKENGNVVKNRELVHHFKHYLTDPASKEDARVKFKKFVNTLAQTKTEG-DEKFLILKTRYQNSLDYPRPSQLALSPHSDPR-
Dromel-CG10632  -MELPKELSVAEIRNYMLANECKVTNHALVKHFKKFLTHPQSQNEARKRFKTYVTLLSTIKNEN-NQKFLILRKKYVNECPTDEVVERAVAAAS----
Dropse-CG10632  -MELPKELSIAEIRNYMLVNECKVTNHALVKHFKKFLTHPQSQNEARRRFKTYVTLLSTIKNEN-NQKFLILRKKYLNECPTDEVVERAVAAAN----
Pedhum-CG10632  -MSSSAELSFDAVKDFIILNGGKVRNHDLVKHFKKFLTKPQNR-DARNYFKEIVNEVAVIRTEG-DEKYLILKRRFRPPGLEECFSSSPSSLSLS---
Nasvit-CG10632  -MASPSELSLEEIRKYLLDNGGSARNQDLVKHFKKFLTDPESRVEARNRFKEYINTLATIKTEE-GEKYLVLKKKFRQQNLDLSSPTLSFSGFPT---
Apimel-CG10632  -MATPSELSLEEIRQYLLENGGTARNHDVVKHFKKFLTDPETRVEARNQFKEYVNTLATIKNEE-GEKYLVLKKKYRQLPIDLG-----ISDQIT---
Braflo-CG10632  ~~MADYEFSFDGVKQFIADRGGRVRNVELVTHFRQHLNDPARKAEARELFKDFVNNVAVIRQEE~GEKFLVLKKKYREPSAQVSRVRPLAVEELEYTG
CapspI-CG10632  --MGDSLFNQDIVRDFMISKGGKVRNHDLVSHFKSFLNDPHQKVANRQKFKTFVNTLSSVKLDESGEKVLVLKRKFRNDAPNDATSVPARPSPQ----
Lotgig-CG10632  --MAATCFTLEAVKEFMINNGGRVTNQQLVTHFKAFLNDPVSKVTHREKFKDYVNELATIKLEE-GSKILVLKKKHR---QTENAPVPAPRTSK----
Strpur-CG10632  MADAEEEFSADAVQKFLVSNGGKATNVEVVGHFSKYLNDPEFKVLNRQKFKDYINEVATVKSEK-GEKFLVLKNEFFYREWIEPKHVIIRSAQS----

Nemvec-CG10632  ------------------------------------PRPVSA~~~~~~MPVARHAKMFEEMAAARYPMTR----------------------------
Dappul-CG10632  LPLLPSQPQYVPPPAPHVQHQHYGQPPMDVAPAFRAPPPYRP~~~~~~PPEPAPFRRSSLSGSDLSHSPVNVYPPPQVLPYNN---------------
Tricas-CG10632  -----LALGIPLSPTPSDYSPAKS-------PAHRQPPPYR-~~~~~~PPPPVTSPTN--SLDNVSLSS-----------------------------
Dromel-CG10632  --------GIPEPSSPGGASLNFD-------SPMRQPPPYK-~~~~~~PPPMVTSPPA-VSIAKEHQENYQECV------------------------
Dropse-CG10632  --------NGPEPSSPGGGSITFEGG--SLASPMRQPPPYK-~~~~~~PPPMVTSPSAGVAVHKVQQDNYRECV------------------------
Pedhum-CG10632  ----------SNSKMPSESNTGNNN------LSVRQPPPYRA~~~~~~PPPPVTPTKSPQSSSPLTPVSP----------------------------
Nasvit-CG10632  ----------SPISTPNLSTPTS---------PLREPPPYR-~~~~~~PPPVAPLSPISNTSSPVRRYTQDPPVGLPLSLPVEPPVGLPIGIPQKPPI
Apimel-CG10632  ----------SPINTPDISTPLS---------PLRVPPPYR-~~~~~~PPPPAPLSPPTNNTNREESCSN---------FDRE---------------
Braflo-CG10632  ~~~~~~~~~~~~~~~EEPQNNYPYQ~~~~~~~PPEQPSSYLAFQEPPLPPPPALASPVRRDWDVPAPPAEQEVGGS~~~~~~~~~~~~~~~~~~~~~~
CapspI-CG10632  ------------LHRPKKQKMLDSF-------PLRAPSPEED~~~~~~KSAASPEQQPQEPQEQTDPVPR----------------------------
Lotgig-CG10632  ------------VDRAQSE-----------------------~~~~~~----PPGQPAKKNNESGQGLSK----------------------------
Strpur-CG10632  -------------QAPKPSWPPP------------APEQPME~~~~~~TEPVAPARRKKKSRDHSSAHQN----------------------------

Nemvec-CG10632  -------------SHSEDHAMNELPDKE------------------------NRPRELKALSDMVKSGSIDSMDSVGSTHSTPKMGRKISQP------
Dappul-CG10632  -------YQPPPPPAIPPLPSQKLPAPPPPPAVHHFVRQTSLPSDDFIPASAYLLPQQQPMPERSVSIQDFSSHHVQDLTSGGSPGPVVPPRRLKAQR
Tricas-CG10632  ------------IMSLQDVTPQAPPRRK---------QRSFDE------------------DSTPRQINNQE--------------------------
Dromel-CG10632  ---------DEFTAAIKRIEPARLERRT---------SVKSED--LDQQEKPEKTS----RSNSIDVIDNKENIPRFSFSSEASSASSTSIEKPEM--
Dropse-CG10632  ---------DEFTAAIQRIDPARLERQP---------ATKTEEDAATSQEQPPSVSGSVSRSNSVDETGNKENIPRFSFSSGASTDSS----------
Pedhum-CG10632  -------------TSIFPTSAISPSSPD---------KTNVED-------PPSVPVRKKSLTEKIKKEHHSD-------MSKSSSGDSSTGD------
Nasvit-CG10632  GLPIGVPYNEPVFEDNVSTSASRLPKRE---------MATSSPV-----ASPPVPPRRKSQ-DKIKLANKENQSTDEQTPKKGRSGSESIKE------
Apimel-CG10632  -------------EAAINARKNGVESVE---------AGFSTP-------SPPVPPRRKSQ-DKVKMENKEN-------VDRNRGGSEAVIK------
Braflo-CG10632  ~~~~~~~~~~~PAGWQPPYQQPDLVRRQPSFNDYNQQPPYDEPEQIRRQLSFNQPPNPYEHNDEPLPVNRQSSFKEPVSVARKPSFEKAITHNQFPPH
CapspI-CG10632  ------------IPQETPLNESLEIAAE----------EGQDEVLSGPSLLAEIQNQMAELVERADDPREDTSRENSPVKERNEGSSFVATP------
Lotgig-CG10632  ------------VHSDEQFDRNKERNFD----------TGAN------SLAESTSSVASSLSTGTVSADDDVNDSVISVKDRAKHLNKIDSE------
Strpur-CG10632  -------------ESSGNGRSSPKPKGG----------------------TSPRPTISAPIYTGPVDQHSVVN-------TRAGEKMTEELS------

Nemvec-CG10632  ------------GKRQGDSLERNSQALSEDVFEDPDIDGDDLMNIPT------------------IQ~~~~~~~~~~~~~~~~~~~~~~~~~~~~~~~
Dappul-CG10632  SDDKENHLVSFIFDEEIKISVKERTQRFNKMASEIDLVPKNGSSERR----DSKSKE----------~~~~~~~~~~~~~~~~~~~~~~~~~~~~~~~
Tricas-CG10632  ------------EEKQTPVSVKERLEKFNRMASMEDE--LSPRQAKS----AEK-------KKEKQL~~~~~~~~~~~~~~~~~~~~~~~~~~~~~~~
Dromel-CG10632  --ADPTAPAAVGDVAENPISVKEATRKFNRMASEEEAKIISPPAKKK----PEKQLIEEKDSPEVTL~~~~~~~~~~~~~~~~~~~~~~~~~~~~~~~
Dropse-CG10632  --AAEKVSSNEADTAENPMSVKEATRKFNRMASEEEAKIISPPAKKK----PEKQLIEEKDSPEVTM~~~~~~~~~~~~~~~~~~~~~~~~~~~~~~~
Pedhum-CG10632  --VELECER--------KISVKERMQKFNRMASESDLPKIPSTTTTTTTNNVKKKLDKEDDSSSMTS~~~~~~~~~~~~~~~~~~~~~~~~~~~~~~~
Nasvit-CG10632  --DDQATPTSNQLTPAEQLSVRERMQRFNRMASETDLPARPNASIPS----VKKRTDKQL-------~~~~~~~~~~~~~~~~~~~~~~~~~~~~~~~
Apimel-CG10632  --EDEAVATNPGST--EQLSFRERMQRFNRMASETDLQGRPNGTITP----TKKRSDKQ--------~~~~~~~~~~~~~~~~~~~~~~~~~~~~~~~
Braflo-CG10632  EPSPSPLDDAHSPTFKRPSPPAQSQNGGPRSELPRSDSSSRMETGSSRSDTVSIRSDTASISEKGEAEDDGYGSNVSSVAKRVHFAEYDEYLILHQDF
CapspI-CG10632  -----------EEEEVPGASGGSAEREEDPLAGVEEAAPPSPPSAPNELSSPPPDAVIEVVTPQTQK~~~~~~~~~~~~~~~~~~~~~~~~~~~~~~~
Lotgig-CG10632  -----------SDILLPNQFKKKSGRER---SGGEDDD-----------------------DTHFIS~~~~~~~~~~~~~~~~~~~~~~~~~~~~~~~
Strpur-CG10632  ----------------QIEGVRDARDKFEQESRLQTPTPPKSSSSPS--------------------~~~~~~~~~~~~~~~~~~~~~~~~~~~~~~~

Nemvec-CG10632  ~~~~~~~~~~~~~~~~~LDAIEKEWLLTTARGNRAHIMCLLEQEPLLAKRKDFTSGYTALHWAAKHGRDDVAMMVAKAGAD--VNSKTHGGYTPLHLA
Dappul-CG10632  ~~~~~~~~~~~~~~~~~MEPRAKEWMLATARGDYHAIARLLREEPRLARRRV-----TGLHWAAKHGNDDLIKLLAGTHK---SDVNARTGCTPLHLA
Tricas-CG10632  ~~~~~~~~~~~~~~~~~EPKKCMEWYVTASKGDIQELLKLAQDEPRLVNRKV-----TVLHWGAKHGNPKIIQMFAGKYK---VDVNGKTGYTPLHLA
Dromel-CG10632  ~~~~~~~~~~~~~~~~~AHPKAKEWIVSMAKANYQELAKMASEYPELVK-LQ-----TALHWAAKHGNEDVVKLIAGTYK---ADVNARTGYTPLHLA
Dropse-CG10632  ~~~~~~~~~~~~~~~~~AHPKAKEWIVSMAKANYQELAKLASEYPELVK-LQ-----TALHWAAKHGNEDVVKLIAGTYK---ADVNAKTGYTPLHLA
Pedhum-CG10632  ~~~~~~~~~~~~~~~~~IDPKTREWLVHAAKGEYQVLAKLAAECPKLVRTK------TALHWAAKHGNENLVKLIAGTYK---LNPNVRTGYTPLHLA
Nasvit-CG10632  ~~~~~~~~~~~~~~~~~MDGKSREWLVRAAQGDYQALAKLAAEEPRLARTK------TALHWGAKHGDENIVKLIAGTCKDWIKSVNETSGYTPLHIA
Apimel-CG10632  ~~~~~~~~~~~~~~~~~LDGKSREWLVRAAQGDYQALAKLAAEEPRLTRLKV-----TALHWAAKHGDENIVKLIAGTYKDYIKSVNETSGYTPLHIA
Braflo-CG10632  VPSSQLYYHHTHRPQLMLDVLEKEWMLKAAVGNVSALRRLLSQDPNLASKK~~~~~~TALHWGAKHGNQEIVALMADAGL~~~~DVNLRSGYTPLHLA
CapspI-CG10632  ~~~~~~~~~~~~~~~~~LSAADREWITAAAAADYHPMNKLLVSNPQLAKRK------TPLHWACKQGKTEVVKLICAYPE---VNVNARSGYTPLHLA
Lotgig-CG10632  ~~~~~~~~~~~~~~~~~YGAEEKEWMLTCCKADYHEMNRLLSKDSSLAKIR------TALHWASKHGKGEVIKLLVGKPG---VNINQRSGYTPLHLA
Strpur-CG10632  ~~~~~~~~~~~~~~~~~-SASATTTTTPATTATSATGDAEAAPQPDDIPPAP-----SALHWGAKHGKKEMIRLVVDRGI----DVNLRSGYTPLHVA

Nemvec-CG10632  AMHGNDRVIKVLVDDFKADINARDFSGRKPRQVAKDTLTIEAQRRLEHVLTLSDSSSESGYSSQGQQTPKYGSLNSIVTPSAALMGYYGGG-------
Dappul-CG10632  AMQGHADVVDLLVKAYGADSNMRDYSGKKPHQYLSRPDTVISIDTFR-VNPEKKRHLAKDL-----GFLRIGSLNVRVKKTTEAFNNLLSSAGG----
Tricas-CG10632  AQFGHRDIYDMLINEYHADSKIRDYSGRLPAYYLQSKDQKIRKDNIRKIKGRKKPTIDKDL-----GFLRIGSLNVRVKKTTEAFSNFLGVGSGS---
Dromel-CG10632  TQFGRDNIFELLWNVYKANRDIMDWSGNKPLDYSRQR-SSVSASTCSKIKARKKHAIEKDL-----GFLRIGSLNVRVKKTTEAFSNFLGVGNGSGVA
Dropse-CG10632  TQFGRDNIFELLWNVYKANRDIMDWSGNKPLDYSRQR-PSVSASTCSKIKARKKHTIEKDL-----GFLRIGSLNVRVKKTTEAFSNFLGVGNGSGVA
Pedhum-CG10632  MQYEHEEIFDLLVQVYAADQNLRDWSGKKPRQYRANMDTSVSADTFRKIKARKKY-VEKES-----GFLRIGSLNVRVKRTTEAFSNYF---------
Nasvit-CG10632  LQFDHENIFNLLVQVYGANQDIRDYSGKKPRQYSVSQKAAVSQDTMRKIKARKKHAIEKDL-----GFLRIGSLNVRVKRTTEAFNQFLGV-------
Apimel-CG10632  MQFDHENIFNLLVQVYAANQEIRDYSGKKARQYLVSQEAAVSQDTFRKIKARKKHAIEKDL-----GFLRIGSLNVRVKRTTEAFSQFLGV-------
Braflo-CG10632  AMYGHDHIVKLLIDAYDADVTIRDYSGRRPGQRLRPGSSTNVDEVV~~~~~~~~~~~~~~~~~~~~QRLVSDTLMSFPFISFCRGLR~~~~~~~~~~~
CapspI-CG10632  AMQGHEEVIEMLVQAYGADSNLRDYSGKKAKQYLRNSASTRAQQLLQNRRPSAASAGAAP--------FRRTGSYSSKIRSTGHFGIAAGT-------
Lotgig-CG10632  AMHGHEDIIELLVQNYKADSNIRDYSGKKAKQYLKNSASSRTQQMLLSRRLAAAGSVKGQSLDD--SFIRTTLRKSNRAKAISSLIQATQS-------
Strpur-CG10632  AMHNKEAIMQFLVSEYHADINKRDFSGRKPRHYLQSSSSNYIKQMMLGQSTAEVIQPHVLMS--------LAEPKQEEKRHSKIGS------------
```

```
Nemvec-CG10632   ---------------------------------------------------------------SSEAPLSVAQPPAESGDKHRDRTSSLGKFLHKKK-
Dappul-CG10632   --------------------------------------------------------------SGTFSTTKMYNKPWGSIDNLPIS--KMMPPPNKQG-
Tricas-CG10632   -------------------------------------------------------------VTALETTSEKVHKGWGSADNVNQE-ENINPGPKINT-
Dromel-CG10632   PTGYGNGGSAVAANRHHPRSHRAPHQRHHHHVGTTRSRHPNQRAAMSTPYATNGGLPSRASVPNSRNIYDGVHKSWGSADNIPHRSEDLMPPPKAVEY
Dropse-CG10632   PTGYGSGG-ATTANRHHQRSHQHPHQRHHHHVGTTRSRHPNQRVAT-TPYGTNSIPGSRASVPNNRNIYDVVHKSWGSADNITQRTEDLMPPPKSVE-
Pedhum-CG10632   ---------------------------------------------------------------LGGQSSDKLLKSWGSADNVHENDKKLMQPPKSGA-
Nasvit-CG10632   ------------------------------------------------------------TTKTSSNNSEKIHKTWGSADNLPAD--KMMPPPKYAQ-
Apimel-CG10632   ------------------------------------------------------------ATSSASSNHEKIHKSWGSADNVQLDQ-KMMPPPKYAP-
Braflo-CG10632   ~~~~~~~~~~~~~~~~~~~~~~~~~~~~~~~~~~~~~~~~~~~~~~~~~~~~~~~~~~~~~~~~~~~~~~~~~~~~~~~~~~~~~~~~~~~~~~~~~~
CapspI-CG10632   --------------------------------------------------------------AVRNSWAQLAQAERRGSS---QESLPSPKPSPKQSP
Lotgig-CG10632   --------------------------------------------------------------KVHRSKSTLLRSSWGSSSEHVDDDQPTPPGSSHSSP
Strpur-CG10632   ---------------------------------------------------------------LLRSSFRHSFRIWGSAENLEDKNHKRDRTPPVTPP

Nemvec-CG10632   ---KSKDHRQPMPSTPYP------------SNSSAGLLIGSPIELQRMDEERRENELDMK--------------------------RTQSDPNLLHDE
Dappul-CG10632   ----TVKKKRPKDWPQP-------------LISGPTN--LHHSNSFNGRPLQPTLIENK------------------------EEYENDSDSAYGFGN
Tricas-CG10632   ---VKKKSKRP-YSSG--------------MNSTP-------GTPRQMSRNFSSFHTN----------------------------DSDSDSAAGFDS
Dromel-CG10632   ISKRNKSSKRSSYASN--------------TTDSPRDSICSSNSSSNLNGGYSSMPTTPNQLRAPKGIAAS------------FAVDSDSDSACGFDS
Dropse-CG10632   -TKRNKSSKRSSYASN--------------ATNSPRDSICSSNSSSNLNGGYSSMPTTPNQVRAPKGMDHTS-----------FTADSDSDSACGFDS
Pedhum-CG10632   --IKKRKSKREVDYAH--------------SRSTPTTPVQHRSSTIS-------------------VGSVEN----------YEQQDSDSDTAAGFGA
Nasvit-CG10632   --IKKRRSRRATLISSQAVPITSSNSSSAQTASQPSTPTLLGKSATAGRRPASVSALTTGGLLAQTLSSGISTGSIQKDHRQQQQNDSDSDGACGFDS
Apimel-CG10632   --IKKRRSRRAQDFSS---------LREHQTASQPTTPLLQGKVSRSSHQQMHRRPTST-----TAVSSAAS--------RTQQSNDSDSDTACGFDS
Braflo-CG10632   ~~~~~~~~~~~~~~~~~~~~~~~~~~~~~~~~~~~~~~~~~~~~~~~~~~~~~~~~~~~~~~~~~~~~~~~~~~~~~~~~~~~~KAESDPDVTGGFA
CapspI-CG10632   QTWRKQSHS-GSQELTMP-----------PPRDLPVRHRRQSSRRDNKE-NHSDSELGAG-----------------------GLSGSETDLTVGMDS
Lotgig-CG10632   TTSRKGSFSDSSRDKTLM-----------PPPSAPTQRRNKSASRDNLQSTQSASAIDPR-----------------------SLSGARSGSDSALSS
Strpur-CG10632   PSPKNRPKTSHSVNHSQS------------FLMPPPSSYTVPNRGRKLRSTDKDRTENMR--------------------------KSESNPDMIVET

Nemvec-CG10632   V----------------
Dappul-CG10632   NWQSS------------
Tricas-CG10632   QWKH-------------
Dromel-CG10632   TWSVNCRGSSSSNPSQS
Dropse-CG10632   SWSVSHLAAVGNNPTQS
Pedhum-CG10632   QWQSKM-----------
Nasvit-CG10632   AWRGSA-----------
Apimel-CG10632   AWRGSAQL---------
Braflo-CG10632
CapspI-CG10632   AASYPNISVQKASYV--
Lotgig-CG10632   KNTYID-----------
Strpur-CG10632   TPTTFV-----------
```
